# Comparative Proteomics Reveals that Phosphorylation of β Carbonic Anhydrase 1 Might be Important for Adaptation to Drought Stress in *Brassica napus*

**DOI:** 10.1038/srep39024

**Published:** 2016-12-14

**Authors:** Limin Wang, Xiang Jin, Qingbin Li, Xuchu Wang, Zaiyun Li, Xiaoming Wu

**Affiliations:** 1 Oil Crops Research Institute of the Chinese Academy of Agricultural Sciences, Key Laboratory of Biology and Genetic Improvement of Oil Crops, Ministry of Agriculture, Wuhan 430062, China; 2 National Key Lab of Crop Genetic Improvement, National Center of Crop Molecular Breeding, National Center of Oil Crop Improvement, College of Plant Science and Technology, Huazhong Agricultural University, Wuhan 430070, China; 3 Institute of Tropical Biosciences and Biotechnology, Chinese Academy of Tropical Agricultural Sciences, Haikou, Hainan 571101, China; 4 State Key Laboratory of Agrobiotechnology, College of Biological Sciences, China Agricultural University, Beijing 100193, China

## Abstract

Little is known about the mechanism of drought tolerance in rapeseed (*Brassica napus* L.). In this study, different morphological and physiological responses to drought stress were studied in three rapeseed cultivars. For the cultivar 2AF009 with high drought tolerance, comparative proteomic analyses were conducted to determine the molecular mechanism behind. Approximately 138 differentially abundant proteins (DAPs) and 1232 phosphoproteins containing 4469 phosphopeptides were identified. Furthermore, 337 phosphoproteins containing 547 phosphorylation sites demonstrated significant changes. These drought-responsive DAPs and phosphoproteins were mainly involved in signal transduction, photosynthesis, and glutathione-ascorbate metabolism. Notably, 9 DAPs were also identified as drought-responsive phosphoproteins, especially beta carbonic anhydrase 1 (βCA1), which was represented by eight distinct protein spots with different abundant levels during drought stress. Tyr207 phosphorylated site of βCA1 was down-regulated at the phosphorylation level during drought stress, which was also located in the substrate-binding active region of three-dimensional (3D) structure. Moreover, drought stress inhibited CA activity. We concluded that Tyr207 was the most likely phosphorylation target affecting the enzyme activity, and phosphorylation of βCA1 might be important for the response to drought stress in rapeseed. The study provided a new clue for the drought tolerance mechanism in *B.napus*.

Drought is a major environmental factor in impairing growth, development, and productivity which can lead to a loss of grain yield in many agriculturally important crops^1^. Even worse, global climate change is predicted to lead to extreme temperatures and severe prolonged drought in some parts of the world, which will have a dramatic impact on crop growth and productivity^2^. Plant responses to drought stress is a complex mechanism, triggering a series of physiological, biochemical and molecular changes^3^,^4^. At physiological level, plant drought response and defense mechanisms involve in photosynthesis, respiration, water relations, turgor, and osmotic adjustment^5^. At biochemical level, accumulation of stress metabolites could preserve homeostasis, such as glutathione, betaine, proline, raffinose, and galactinol^6^. Defense- and detoxification-related enzymes can scavenge reactive oxygen species (ROS), including superoxide dismutase (SOD), ascorbate peroxidase (APX), catalase (CAT), glutathione reductase (GR), and peroxidase (POD)^7^. At molecular level, drought-inducible proteins can be divided into two main categories: functional proteins like chaperones, detoxification enzymes, mRNA-binding proteins, and osmotin; regulatory proteins like transcription factors, protein kinases, protein phosphatases, and signal-related proteins^8^,^9^. Therefore, better understanding of crop drought tolerance at many different levels is needed.

As the study of functional genomics has progressed, proteomic approach has become one of the best strategies for revealing dynamic changes in proteins in response to different stressors, which can provide useful information for understanding diverse plant stress response mechanisms^10^,^11^. The studies at the proteome level in plants under drought stress have been carried out in many crops, including rice^12^, maize^10^, wheat^11^, barley^9^, cotton^13^, chickpea^14^, faba bean^15^, and sugar beet^16^. Many proteomics studies on the effects of abiotic stresses are available for rapeseed, but only a few proteomic analyses under drought stress were conducted. Physiological and proteome analyses were performed to understand the crosstalk and specificity in the early response of plants to salinity and drought in *B.napus*^17^. A two-dimensional gel electrophoresis (2-DE) proteomic approach was used to study the drought stress responses of rapeseed roots of drought-sensitive and drought-tolerant genotypes, which provided limited information on drought tolerance due to technological limitations^18^. The effects of gradual drought stress conditions on the phenotype, physiological, proteome profiles, and post-translational modification (PTM) were investigated, which provided evidence for the role of PTMs in modulating rapeseed stress responses^6^.

Rapeseed is an important agricultural crop grown primarily for its edible oil and the seed meal remaining after oil extraction, which is used as a protein source for the livestock feed industry^18^. Rapeseed crops are mostly affected by drought, due to the fact that they are mainly grown in arid and semiarid areas^2^, which were particularly sensitive to drought during the early vegetative growth stage, as many processes, including photosynthesis, stomatal conductance, transpiration, chlorophyll fluorescence, protein synthesis, and metabolite accumulation, were negatively influenced under such conditions^19^,^20^,^21^. Rapeseed (*Brassica napus* L.; 2n = 4x = 38; AACC) has a large genome (approximately 101,040 gene sequences), which hinders genomic and proteomic research. In particular, the release of whole-genome sequences accelerates proteomic studies in rapeseed^6^. Here, we performed integrative morphological, physiological, proteomic, and phosphoproteomic analyses of the effects of drought stress conditions at early growth stage of *B. napus* seedlings. Our results provide new insights into the molecular mechanisms underlying drought stress responses in rapeseed.

## Results

### Comparison of different rapeseed cultivars in response to drought stress

**Figure 1:**
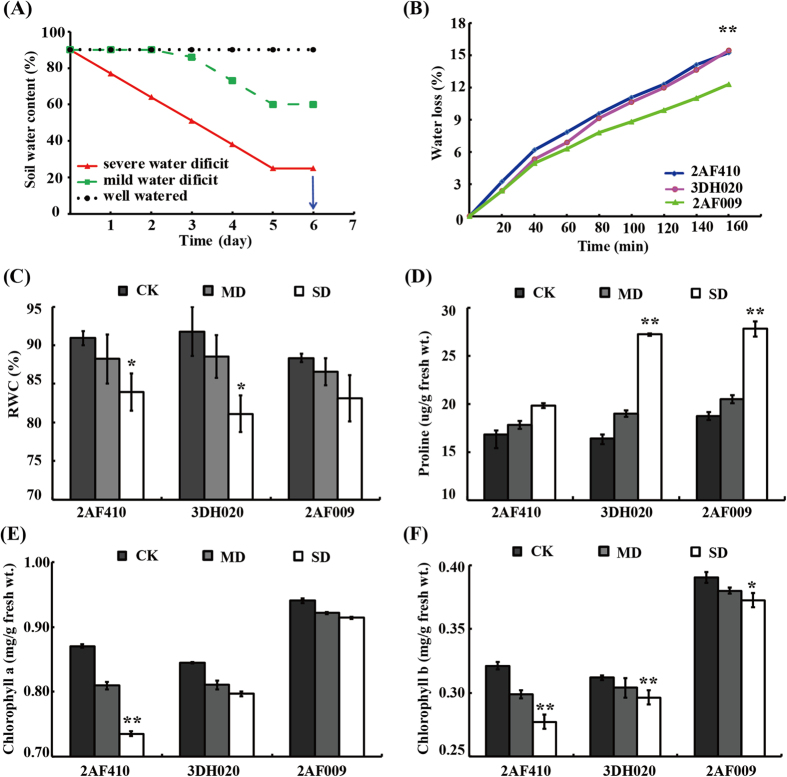
Measurement of physiological changes. (**A**) Illustration of drought treatments. For the control group, soil water content was kept about 90%. For mild and severe drought treatment, water was withheld at day 0 and day 2 respectively, and the progress of drought was monitored by soil moisture. Finally, leaves were collected at day 6. (**B**) Water loss rates in the excised leaves. Leaves were detached and exposed to cool white light at 22 °C, weighed to determine water loss rates (percent of fresh weight, FW%) at different time points. Asterisk indicates water loss rates of different cultivars are significantly different (*p-value < 0.05, **p-value < 0.01). Three independent biological replicates were performed for each time point (n = 3). (**C**) RWC of leaves was measured after drought treatment at day 6. (**D**) Proline accumulation analysis. (**E**) Chlorophyll *a* content analysis. (**F**) Chlorophyll *b* content analysis. Statistically significant differences relative to the control were calculated by independent Student’s *t* tests. Asterisk indicates control and drought-stressed samples are significantly different (*p-value < 0.05, **p-value < 0.01). The values are presented as means ± standard error (SE); n = 3 for all groups. The bars represent the SE.

The drought treatment method used to study the morphological and physiological response in *B. napus* is presented in Fig. 1A. Morphological patterns of young plants from three *B. napus* cultivars (2AF009, 3DH020, and 2AF410) under drought treatments are depicted in Supplemental Fig. S1. The leaves of different cultivars exhibited different response patterns to drought stress. Under mild stress condition, the leaves of 3DH020 and 2AF410 wilted and turned yellow, but those of 2AF009 remained green. Under severe drought stress, the leaves of 3DH020 and 2AF410 fell off near the bottom edge while those of 2AF009 continued to grow but wilted and turned yellow to some extent (Fig. S1). The result indicated that drought treatment caused noticeably stronger senescence of the lower leaves in 3DH020 and 2AF410 than 2AF009, suggesting that 2AF009 is more tolerant to drought.

Physiological changes among the three cultivars were determined (Fig.1). Water loss in the excised leaves from 2AF009, 3DH020 and 2AF410 was 12.25%, 15.42%, and 15.22%, respectively, at 160 min time point. By comparison, the leaf water loss in 3DH020 and 2AF410 was significantly higher compared to 2AF009 at the time point from 20 to 160 min after leaf detachment (Fig. 1B). The leaf relative water content (RWC) is considered to be the best integrated measure of plant water status^12^. In this experiment, the RWC was lower after drought treatment (Fig. 1C). Therefore, our results demonstrated that leaves from different cultivars had different responses to different degrees of drought stress. Under mild drought stress condition, RWC decreased slightly, by 2.0–3.0%. However, RWC in the leaves of 2AF410 and 3DH020 decreased 7.6% and 11.6%, respectively, while the RWC in 2AF009 only decreased 5.8% under severe drought stress condition (Fig. 1C), suggesting that 2AF009 had a greater ability to retain water. Proline is an important osmoprotective molecule under drought stress conditions^22^. Proline content was found to increase with the increased intensity of drought treatment in these cultivars. Maximum proline accumulation was observed in 2AF009 during drought stress (Fig. 1D). In addition, leaf chlorophyll content was different under different degrees of drought stress. Under well-watered condition, 2AF009 maintained a significantly higher content of chlorophyll *a* and chlorophyll *b* than 3DH020 and 2AF410 (Fig. 1E and 1F). 2 AF410 showed a dramatic decline in chlorophyll *a* and chlorophyll *b* content between the mild drought stress group (MD) and the severe drought stress group (SD) relative to the control groups (CK), but 2AF009 decreased slightly. The level of chlorophyll *b* was not significantly changed even under extreme drought stress. In all, 2AF009 exhibited lower water loss rates, a smaller decline in RWC, and a higher proline content, chlorophyll *a* and chlorophyll *b* content than other cultivars. It was concluded that 2AF009 was a potential drought-tolerant cultivar. Together, these results revealed the changes in the biological process of adaptation to drought stress in early growth stage of rapeseed.

### Identification and functional analysis of drought-responsive proteins

**Figure 2:**
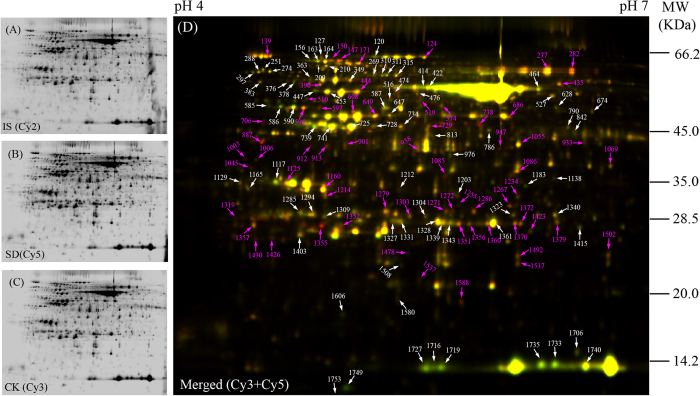
Representative 2D-DIGE image of rapeseed leaves proteins under drought treatment. Leaf proteins were labeled with different fluorescence dyes. The combined images of each sample with internal standard (IS, labeled with Cy2) are presented (**A**). Proteins of Cy5-labeled SD group samples (**C**) were compared with those of Cy3-labeled CK samples (**B**) by 2D-DIGE using 24-cm pH 4-7 IPG strips and 12.5% SDS-PAGE gel. The 138 DAPs were marked with arrows (red arrows, protein spots significantly up-regulated upon drought treatment; white arrows, down-regulated protein spots) presented in merged 2D-DIGE gel (**D**). All of the labeled protein spots were identified by MS/MS, and the detailed information were listed in Supplemental Table S1 and Fig. S2.

To reveal the drought-tolerant mechanisms of young rapeseed plant at the protein level, a comparative proteomics analysis combining two-dimensional difference gel electrophoresis (2D-DIGE) and MS was carried out on the highly drought-tolerant cultivar 2AF009 to determine the DAPs in leaves. Typical DIGE gels for each treatment and the combined images are presented (Fig. 2A–C). Approximately 4000 reproducible protein spots were detected and matched among all gels. The numbers of highly reproducible protein spots were determined to be 4300 ± 65, 4010 ± 85, and 4480 ± 50 in the leaves of plants from the drought treatment groups CK, MD, and SD, respectively (Fig. 2). DIGE can detect protein differences as low as 1.2-fold change^23^. So, only spots with good reproducibility and for which the changed abundance ratio of >1.2 or < 0.83 with P<0.05 were identified as DAPs in the subsequent studies. Finally, 138 DAPs were successfully identified via MALDI TOF/TOF MS, including 107 DAPs in the SD group and 85 DAPs in the MD group (Fig. 2; Fig. S2; Table S1). These DAPs represented 98 unique proteins. Most of the drought-responsive proteins changed in abundance rather than absence/presence in 2D-DIGE gels. To evaluate the quality of the protein identification by MALDI TOF/TOF MS, the theoretical and experimental ratios of molecular weight (M*r*) and isoelectric point (*p*I), respectively, were determined (Table S1). These ratios were presented as radar axis labels (the M*r* ratio for the radial value and the *p*I ratio for the annular value) in a radial chart (Fig. S3A). When the theoretical and experimental values of the identified proteins were the same, both the radial values and the annular values would be 1.0 and all these identified proteins would be located on the cyclical line 1.0 in radial chart. The closer a spot was to line 1.0, the greater the certainty that the identification made by means of MS/database searching would be the MS identification obtained. More than 90% of the identified protein spots were closely located on the cyclical line 1.0, indicating the high quality of the MS data (Fig. S3A). To survey the distribution of drought responsive proteins across the different degrees of drought stress, proteomic profiles were compared and a Venn diagram was constructed to show the dynamics of the number of DAPs at different degree drought-treatment points (Fig. S3B). Of the 138 protein spots, 54 protein spots were present in all groups, 31 were up or down regulated only in the MD group and 53 were present only in the SD group (Fig. S3B). The results indicated that the number of DAPs increases from mild to severe drought stresses.

Based on the COG (cluster of orthologous groups) functional catalogue, the 98 proteins identified were classified into 15 groups based on their main cellular functions (Table S1 and Fig. S3C). The largest group (29% of all the proteins) was involved in post-translational modification, protein turnover, and chaperones, followed by proteins involved in energy production and conversion (17%) and carbohydrate transport and metabolism (13%); a substantial portion of the proteins participated in inorganic ion transport (10%) or translation, ribosomal structure and biogenesis (3%). These results indicated that post-translational modification, energy conversion and carbohydrate metabolism might play important roles in rapeseed leaves in response to drought stress. The subcellular location of these proteins was also predicted by CELLO. Among them, the largest portion was located in the chloroplast, followed by cytoplasm. In addition, several proteins were located in the mitochondria, plasma membrane, extracellular space, endoplasmic reticulum (ER), and vacuole, respectively (Table S1 and Fig. S3D). These results suggested that a large number of DAPs were related to post-translational modifications, energy conversion and carbohydrate metabolism and were mainly located in the chloroplast and cytoplasm.

Among the 98 proteins, 20 unique DAPs including 35 protein spots (Table S2), with different isoelectric points and molecular weights were identified using experimental values from two to eight protein spots on the same gel. This phenomenon likely resulted from the presence of different protein isoforms, or post-translational modification. These proteins were mainly included ATP synthase (7 protein spots), ribulose bisphophate carboxylase (Rubisco, 10 protein spots), ribulose bisphophate carboxylase activase (RCA, 8 protein spots), phosphoglycerate kinase (2 protein spots), fructose bisphosphate aldolase (3 protein spots), triosephosphate isomerase (2 protein spots), trigger factor like protein (TIG, 2 protein spots), oxygen-evolving enhancer protein 1–1 (PSBO1, 2 protein spots), chaperonin 60 subunit (CPN60, 2 protein spots) and βCA1 (8 protein spots). At the biological level, they were mostly related to photosynthesis and carbon fixation showed phosphatase, kinase and phosphate-binding activity. We concluded that phosphorylation of proteins led to multiple isoforms might participate in drought stress regulation network. Among them, βCA1 (GI No. 297789439) was the largest group of isoforms, which was identified at eight spots (1327, 1331, 1328, 1339, 1343, 1360, 1361, 1370) with the same or very similar molecular weights, and thus was considered to have at least eight protein isoforms. The results suggested that the eight protein isoforms of βCA1 that might be caused by protein phosphorylation were important for the drought stress response.

### Comparing drought stress response at the protein and mRNA levels

**Figure 3:**
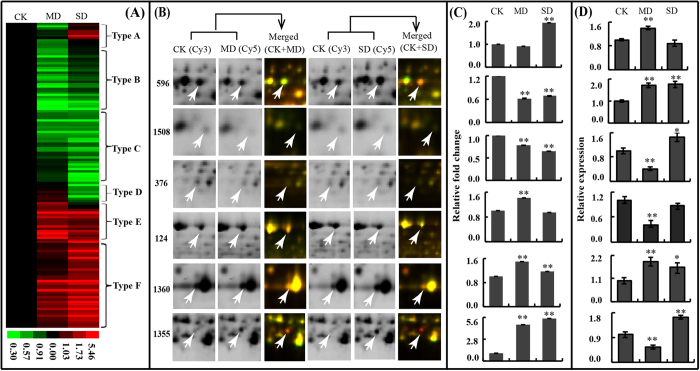
Clustering and expression profile of DAPs. (**A**) Hierarchical clustering of DEP spots under mild and severe drought treatment (MD and SD). Six dominant expression profile types are determined (type A to type F). (**B**) Enlarged areas of representative protein spots (arrow labeled) in 2D-DIGE gels of MD and SD, corresponding to the changed types A (spot 596, RCA), B (spot 1508, uncharacterized protein), C (spot 376, TIG), D (spot 124, TKL-1), E (spot 1360, βCA1), and type F (spot 1355, FER1), respectively. (**C**) The relative expression fold changes of protein spots in Figure 3B at protein level. (**D**) The relative transcription levels of proteins (spots 596, 1508, 376, 124, 1360 and 1355) that determined by qRT-PCR assay. The relative expression levels of CK was set to 1 in (**C**) and (**D**). Asterisk indicates control and drought-stressed samples are significantly different (*p-value < 0.05, **p-value < 0.01). The values are presented as means ± standard error (SE); n = 3 for all groups. The bars represent the SE.

To visualize the 138 DAPs changed patterns, hierarchical clustering was used to analyse the proteome dataset based on methods as described elsewhere^24^. The analysis formed six patterns (named type A-F hereafter) in response to drought stimulus (Fig. 3A; Table S1). To improve understandability of the six patterns, six proteins that represent the 6 clusters were selected to visualize the change in protein abundance in the corresponding gel area (Fig. 3B). Type A included 14 protein spots with a decreased relative abundance in the MD group but an increased relative abundance in the SD group under drought treatment. Type B and Type C included 62 protein spots that mainly exhibited a lower relative abundance under drought treatment. Type D included 8 protein spots exhibited increased relative abundance in the MD group but decreased relative abundance in the SD group under drought treatment. Types E and F included 54 protein spots that were mostly higher in relative abundance under drought treatment. To determine whether some of the drought-responsive proteins were regulated at posttranscriptional level, we performed quantitative RT-PCR analysis of RNA expression. Expression analyses of six proteins and the abundance of the cognate transcripts are highlighted in Fig. 3C and D. RCA (spot 596), representing type A, had reverse trends at the transcriptional and translational levels. An uncharacterized protein (spot 1508) in type B displayed different trends at the transcriptional and translational levels. TIG (spot 376) representing type C showed a similar trend under mild drought treatment and a reverse trend in severe drought treatment at the transcriptional and translational levels. Transketolase-1 (TKL-1, spot 124), representing type D, displayed a reverse trend under drought stress. βCA1 (spot 1360), representing type E, displayed a similar trend at the transcriptional and translational levels. Ferritin-1 (FER1, spot 1355), which was a typical example of type F, showed a reverse trend under mild drought treatment and a similar trend under severe drought treatment at the transcriptional and translational levels (Fig. 3; Table S1). These results suggested that drought-induced changes in the abundances of certain proteins were very different from the changes in their cognate transcripts, indicating the importance of post-translational modifications in controlling the final functions of these genes.

### Phosphoproteins identification and screening with phosphorylation level significantly changed

During the process of analysis and summing-up these results, we noticed that protein phosphorylation modifications might play an important role in controlling the final functions in a drought stress response. To analyse the phosphorylation modification of rapeseed in response to drought stress, phosphoproteomics analysis was carried out in rapeseed leaves under different drought treatments. In total, 4469 phosphorylated peptides corresponding to 1232 phosphoproteins were determined (Supplementary Data S1). These identifications were considered to be valid with 5.0% FDR at the protein threshold and 1.0% FDR at peptide threshold, as well as at least 2 confirmed peptides in one protein. To our knowledge, this represents the largest number of phosphopeptides identified to date in *B. napus* and a significant addition to its phosphoproteome.

**Figure 4:**
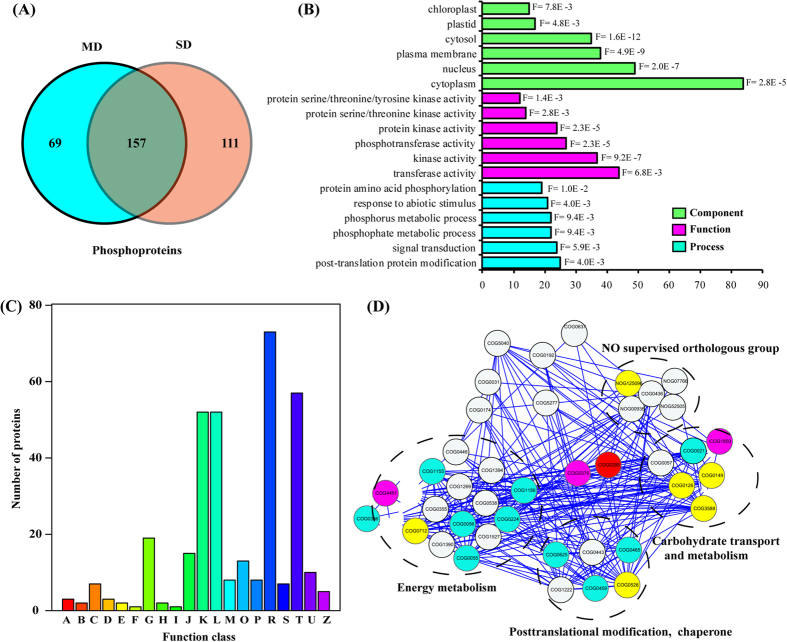
Functional analysis of phosphoproteins under drought treatment. (**A**) Venn diagram of phosphoproteins from CK, MD and SD samples. (**B**) GO enrichment analysis of PLSC phosphoproteins upon drought treatment. **(C)** PLSC phosphoproteins were functionally classified by COG. A, RNA processing and modification; B, Chromatin structure and dynamics; C, Energy production and conversion; D, Cell cycle control, cell division, chromosome partitioning; E, Amino acid transport and metabolism; F, Nucleotide transport and metabolism; G, Carbohydrate transport and metabolism; H, Coenzyme transport and metabolism; I, Lipid transport and metabolism; J, Translation, ribosomal structure and biogenesis; K, Transcription; L, Replication, recombination and repair; M, Cell wall/membrane/envelope biogenesis; O, Posttranslational modification, protein turnover, chaperones; P, Inorganic ion transport and metabolism; R, General function prediction only; S, Function unknown; T, Signal transduction mechanisms; U, Intracellular trafficking, secretion, and vesicular transport; Z, Cytoskeleton. (**D**) PPI network analysis of proteins determined as DAPs by 2D DIGE and detected as phosphoproteins by protein phosphorylation enrichment analysis. All the nodes represent the COGs of DAPs in the course of drought stress, nodes with yellow background color represent the COGs interaction with βCA1 directly, and blue background color represent the COGs of DAPs that were also identified as phosphoproteins. Nodes with purple background color represent the COGs both interaction with βCA1 directly and that were identified as phosphoproteins. Therefore, four major clusters of interacting proteins were constructed, which included energy metabolism, carbohydrate transport and metabolism, posttranslational modification and no supervised orthologous group.

A label-free quantification (LFQ) algorithm was used to compare phosphopeptides abundances between different samples^25^. On the basis of analyses of three biological replicates, only phosphopeptides with a log_2_ fold change (drought stress/CK LFQ intensity, log_2_FC) ≥ 1 or ≤ −1 (p < 0.05) were considered to be significantly altered at the phosphorylation level. In total, 337 phosphoproteins containing 547 phosphorylation sites with phosphorylation level significantly changed (PLSC) were screened out. Of the 547 phosphorylated sites, 295 including 107 down-regulated and 188 up-regulated phosphorylated sites were detected in the MD sample and 347 including 115 down-regulated and 232 up-regulated phosphorylated sites were detected in the SD sample (Supplementary Data S2). Of the 337 phosphoproteins, there were 69, and 111 sample-specific phosphoproteins identified from the MD, and SD samples, respectively. A total of 157 phosphoproteins were identified in both MD and SD groups (Fig. 4A).

### Function analysis of PLSC phosphoproteins

In total, 337 PLSC phosphoproteins were used for GO (gene ontology) annotation and enrichment analysis with AgriGO. The distribution bar charts for biological process, cellular component, and molecular function are shown in Fig. 4B. From the biological process perspective, post-translational protein modification (GO: 0043687, FDR: 0.004), signal transduction (GO: 0007165, FDR: 0.0059), phosphate metabolic process (GO: 0006796, FDR: 0.0094), phosphorus metabolic process (GO: 0006793, FDR: 0.0094), protein amino acid phosphorylation (GO: 0006468, FDR: 0.01), and response to abiotic stimulus (GO: 0009628, FDR: 0.004) were significantly enriched. From the cellular component perspective, cytosol (GO: 0005829, FDR: 1.60E-12), plasma membrane (GO: 0005886, FDR: 4.50E-9), nucleus (GO: 0005634, FDR: 2.0E-7), cytoplasm (GO: 0005737, FDR: 2.80E-5), plastid (GO: 0044435, FDR: 4.80E-3), and chloroplast (GO: 0009507, FDR: 7.80E-3) were highly significantly enriched. From the molecular function perspective, kinase activity (GO: 0016301, FDR: 9.20E-7), transferase activity (GO: 0016740, FDR: 0.0068), protein kinase activity (GO: 0004672, FDR: 2.30E-5), protein serine/threonine kinase activity (GO: 0004674, FDR: 0.0028), and protein serine/threonine/tyrosine kinase activity (GO: 0004712, FDR: 0.0014) were significantly enriched relative to background. Approximately, 337 PLSC phosphoproteins were classified into 20 groups based on COG (cluster of orthologous groups) functional catalogue. (Fig. 4C). The largest group (73 phosphoproteins) was involved in general function prediction (72 phosphoproteins), followed by signal transduction mechanisms (57 phosphoproteins) and transcription (52 phosphoproteins); a substantial portion of the proteins participated in replication, recombination and biogenesis (52 phosphoproteins). These results suggested that PLSC phosphoproteins were highly enriched in response to drought stress and signal transduction process through protein kinase activity and phosphotransferase activity. This indicated that the detailed functions of many drought-responsive PLSC phosphoproteins from rapeseed needed to be elucidated. Based on the above results, rapeseed evolved a series of phosphorylation cascades to respond and adapt to drought stress. A next critical step would combine the PLSC phosphoproteins with the DAPs of 2D-DIGE relationships that underlied the rapeseed response to drought stress regulatory networks.

DAPs of 2D-DIGE and phosphorylated DAPs were used to draw protein-protein interactions (PPI) network representing 62 COGs (Fig. 4D and Table S3). The confidence score was set at the highest level (≥0.90) to improve the creditability of the PPI network. Finally, a complex PPI network, including 40 nodes and 270 edges, was visualized with Cytoscape software. At the same time, three major clusters of interacting proteins were constructed, which included energy production and conversion, carbohydrate transport and metabolism, and posttranslational modification (Fig. 4D). The 13 COGs, including 22 DAPs, were identified as phosphorylation-modification. Among them, βCA1, the largest group of isoforms, was analyzed. COG4451, COG0376 and COG1850 also could interact with βCA1 (COG0288); these represent RBCS, large subunit of ribulose 1, 5 -bisphosphate carboxylase (RBCL), and catalase, respectively. βCA1 (COG0288) was further extracted as a potential interacting protein from the PPI network. Proteins interacting with COG0288 included COG1850, COG0149, COG0126, COG3588, COG4451, COG0712, COG0526, COG0376, and NOG125096, which are mainly involved in energy production and conversion, carbohydrate transport and metabolism. These results suggested that βCA1 could be a protein with many interacted target enzymes that play a role in response to drought in rapeseed seedlings.

### Identification of Phosphorylated DAPs

From these combined data, we examined the overlap in both PLSC phosphoproteins and DAPs from 2D-DIGE in response to drought stress. A total of 25 DAPs in 2D-DIGE, representing 9 unique DAPs were also identified as PLSC phosphoproteins (Table 1), and 7 of them were separately identified from two or more DAP spots. The 9 unique DAPs were mainly involved in RBCS-1B (3 protein spots), translational initiation factor 4A-1 (2 protein spots), RCA (4 protein spots), Phosphoglycerate kinase (2 protein spots), TKL-1 (2 protein spots), Ribulose bisphosphate carboxylase large chain (2 protein spots), and βCA1 (8 protein spots). βCA1 was identified from eight spots (1327, 1331, 1328, 1339, 1343, 1360, 1361, 1370) that showed the same molecular weight (36.1 KDa) but different isoelectric point. It was also a phosphoprotein with a down-regulated Tyr207 phosphorylated site (YGGVGAAIE**y**AVLHLK) at the phosphorylation level in the drought-treated sample. In previous studies, water deficit conditions caused a reduction in plant photosynthetic efficiency and stomatal closure, inhibited Rubisco activity, and disrupted energy balance and distribution^26^. These responses often result in a change in photosynthetic- and energy metabolism-associated protein accumulation, including Rubisco, RCA, TKL-1, and βCA1 (Table S1). Among these, βCA1 as a phosphorylated protein is involved in not only photosynthesis but also the stomatal regulation^27^. What’s more, in plant, it is involved in the rapid conversion of the major forms of Ci maintaining the supply of CO_2_ for rubisco activity and is an upstream regulator of CO_2_-controlled stomatal movements in guard cells^28^. Based on these results, we inferred that phosphorylation of βCA1 might be an important regulator of stomatal closure under drought stress. Protein phosphorylation modification is important in changing and regulating enzyme activity. Thus, a comprehensive analysis was performed to define the relationship of βCA1 phosphorylation and enzyme activity in response to drought stress that underlies these regulatory networks.

### Expression and activity analysis of βCA1 upon drought stress

**Figure 5:**
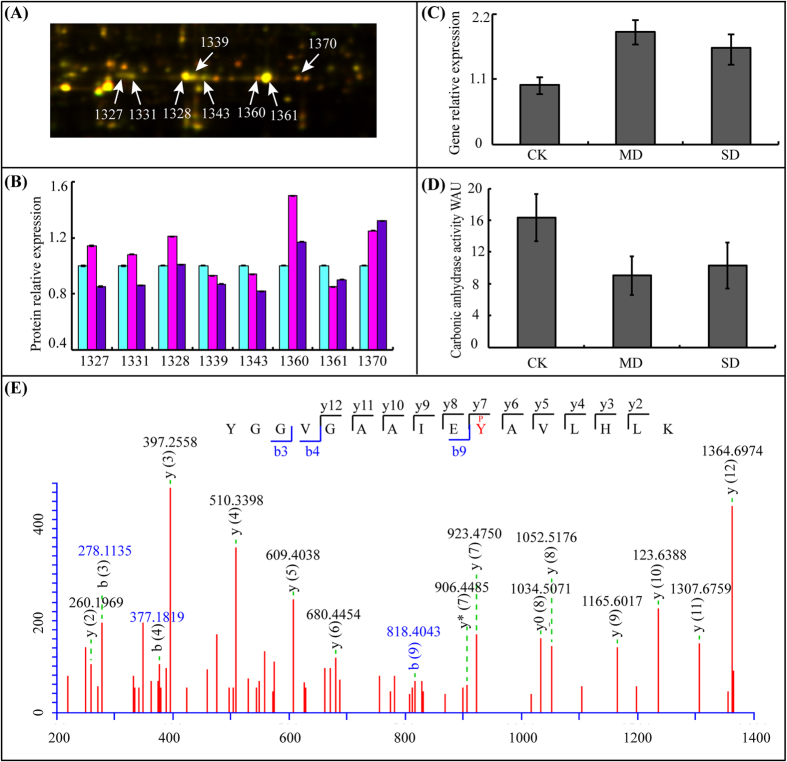
Expression pattern and activity analysis of βCA1. (**A**) The abundance profiles of beta carbonic anhydrase 1 (GI No. 297789439) was provided, and these protein isoforms were identified from eight different spots (1327, 1331, 1328, 1339, 1343, 1360, 1361, and 1370). (**B**) The relative abundance of the eight βCA1 at protein level. (**C**) Relative transcript levels of the βCA1 as determined by qRT-PCR under drought stress treatment. (**D**) Carbonic anhydrase activity assays. (**E**) Phosphopeptide MS/MS spectra map showed the phosphorylation sites at Tyr207. Asterisk indicates control and drought-stressed samples are significantly different (*p-value < 0.05, **p-value < 0.01). The values are presented as means ± standard error (SE); n = 3 for all groups. The bars represent the SE.

βCA1 (GI No. 297789439) was identified from eight different protein spots (1327, 1331, 1328, 1339, 1343, 1360, 1361, and 1370) with a very similar molecular weight but different isoelectric points (Fig. 5A; Table S2). These protein isoforms had different abundance levels under different drought conditions (Fig. 5B). At the gene expression level, βCA1 exhibited an increased relative abundance upon drought treatment (Fig. 5C). The iTRAQ results showed that βCA1 had no significant difference (unprovided data). These results demonstrated that protein levels of βCA1 did not necessarily correlate with gene levels. What is more, different isoforms showed different abundance levels under different drought conditions although total βCA1 protein accumulation had no significant difference change, which suggested that protein phosphorylation modifications of βCA1might be important to drought stress response.

The activity of carbonic anhydrase was further examined in rapeseed leaves upon drought stress. In contrast to down-regulated phosphorylated site of βCA1, CA activity decreased in drought-treated sample (Fig. 5D). These results demonstrated a strong correlation between protein activity and phosphorylation modification, which suggested that βCA1 might adjust the protein activity through phosphorylation modification ultimately coordinate specific metabolic processes to help plants adapt to drought stress.

Next, we identified the phosphorylation modification sites of the eight βCA1 protein spots from 2D gels. A total of 15 phosphorylation sites were determined in eight protein spots upon drought stress (Table S4). The results showed that almost all protein spots contained a phosphorylation modification site at Ser223. However, only protein spots 1328, 1339, and 1361 were in high abundance in 2D gel spots, and these had a phosphorylation modification site at Tyr207 (Fig. 5E). It was noteworthy that both CA activity and the protein abundance for spots 1328, 1339, and 1361 exhibited a decreasing trend upon severe drought stress. These results suggested that phosphorylation modification might play an important role not only in different protein isoforms but also in their enzymatic activities.

### Three-dimensional structure prediction of βCA1

**Figure 6:**
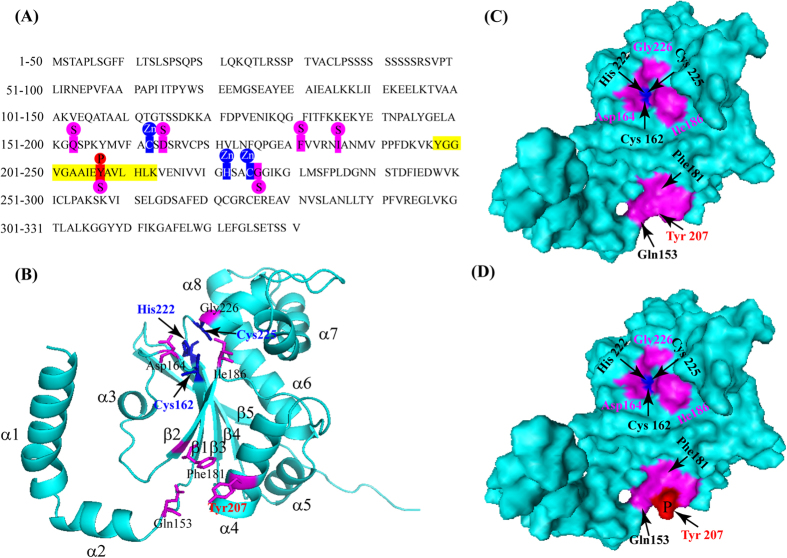
Sequence analysis and three-dimensional structure prediction of βCA1. (**A**) The linear sequence of protein βCA1 including 331 amino acids. The amino acid residues with yellow shadowed represent the phosphopeptide identified by LC-MS, while red ones represent the phosphorylated Tyr207 site. The zinc binding sites were marked with blue box and substrate binding sites were marked with pink background. (**B**) Cartoon diagram of βCA1 monomer. (**C**) Molecular surface of unphosphorylated βCA1 monomer. (**D**) Molecular surface of phosphorylated βCA1 monomer.

The 3D structure was constructed by homology modeling to further analyze the role of phosphorylation site in βCA1. The βCA1 sequence from rapeseed is shown (Fig. 6A). Predicted from sequence conservation, the structure is composed of eight α-helixes (α1-α8) and five β-sheets (β1-β5) (Fig. 6B). The zinc ligands are Cys162, His222, and Cys225, and the substrate-binding active sites are Asp164, Ile186, Gly226, Gln153, Phe181, and Tyr207 (Fig. 6C). In addition, Gln153, Phe181 and Tyr207 are very close in space, forming an active surface (Fig. 6C and D). Interestingly, we found that the phosphorylation site Tyr207 was located in the substrate-binding active centre (Fig. 6D). The results suggested that changes of phosphorylation/dephosphorylation at Tyr207 might affect the binding ability of βCA1 and the substrate. From this evidence, we propose that amino acid Tyr207 is one of the most likely phosphorylation target might affect enzyme activity.

### Integrative analysis of DAPs and phosphoproteins involved in drought regulation

To reveal the DAPs and phosphoprotein pathways, KEGG pathway analysis was conducted and the relevant proteins were used to map the pathways. For DAPs, carbon fixation and photosynthesis, glutathione metabolism, energy metabolism, and ascorbate metabolism were the main pathways (Fig. S4A). For drought-responsive PLSC phosphoproteins, glycolysis/gluconeogenesis, carbon fixation in photosynthetic organisms, pentose phosphate pathway, starch and sucrose metabolism were the main pathways (Fig. S4B). It was worth noting that the starch and sucrose metabolism was highlighted in drought-responsive PLSC phosphoproteins, which contained identified 11 enzymes including starch synthase, beta-amylase, alpha-trehalose-phosphate synthase, sucrose-phosphate synthase, sucrose synthase, phosphoglucomutase. These enzymes mediated signaling events by phosphorylation modification could play critical roles coordinate metabolic processes and physiological responses to help plants adapt to drought stress.

**Figure 7:**
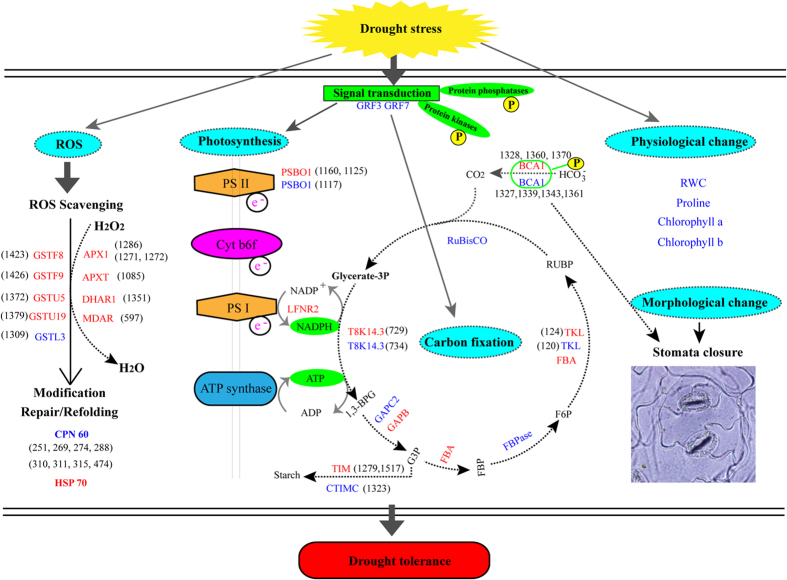
Schematic representation of βCA1 involved in different mechanism pathways for drought tolerance in *B. napus.* The identified DAPs were used to construct the schematic representation. Most differentially expressed proteins were integrated and are indicated in red (up-regulated under drought treatments) or blue (down-regulated), respectively. The abbreviations were provided in the Supplemental Table S1.

Based on the integrated data, a systematic drought tolerance complex network and defense pathway in rapeseed was developed (Fig. 7). Initially, the stress signal was triggered and transduced by multiple phosphorylation cascades, including calcium-dependent protein kinase (CDPK), mitogen-activated protein kinase (MAPK) cascades. Also, signaling events mediates multiple pathway including glycolysis, carbon fixation in photosynthetic organisms, pentose phosphate pathway, starch and sucrose metabolism to respond and adapt to drought stress. Then, these signals resulted in changes of phosphorylation level of various transcription and translation regulation factors that regulated drought stress-related gene expression. Under drought stress, photosynthesis and energy production were depressed. These related proteins, including PSBO1, ribulose bisphosphate carboxylase small chain 1B (RBCS-1B), ferredoxin-NADP reductase, leaf isozyme 2 (LFNR2), ATP synthase subunit alpha (ATPA), ATP synthase subunit beta (ATPB), and ATP synthase gamma chain 1, were significantly induced by drought stress. The redox balance was disrupted and caused glutathione- and ascorbate metabolism-related proteins to be up-regulated to scavenge ROS. These proteins were involving in L-ascorbate peroxidase 1 (APX1, spots 1271 and 1272), monodehydroascorbate reductase (MDAR, spot 597), L-ascorbate peroxidase T (APXT, spot 1085), glutathione S-transferase F9 (GSTF9, spot 1426), glutathione S-transferase U19 (GSTU19, spots 1352 and 1379), glutathione S-transferase F8 (GSTF8, spot1423), and glutathione S-transferase U5 (GSTU5, spot1372). Finally, the physiological and biochemical changes for the growth adaption upon drought stress involved the reduced RWC and chlorophyll content and the increased proline content.

## Discussion

### Rapeseed exhibited protective growth to adaptation to drought stress

Controlled growth is an adaptive response when the plant is subjected to drought stress^29^. In nature, for a plant to sacrifice a part of its structure constitutes an adaptive strategy to survive a stress episode^29^. In this research, rapeseed reduced elder leaves growth even sacrifice them to survive when subjected to severe drought stress (Fig. S1). In other words, rapeseed gave priority to ensure the physiological activities to continue normally when suffered drought stress, which suggested an adaptive response. Also, a series of physiological, biochemical, and molecular changes were triggered to maintain plant growth and development. In our results, the biochemical responses of rapeseed seedlings under drought stress were consistent with previous studies in other *Brassica* crops^6^, such as the accumulation of osmoprotectants, and lowered contents of RWC and chlorophyll. Rapeseed could use physiological modifications in a similar way to adapt to soil water deficits. The maintenance of a high plant water status during stress is an important defensive mechanism to retain enough water by minimizing water loss^15^. Stomatal closure under drought is also an avoidance strategy to save water and maintain turgor. Proline accumulation reduced the toxic effects and lowered the generation of free radicals formed by drought stress (Fig. 1).

### Phosphoproteins involved in signal transduction, starch and sucrose metabolism

Reversible modification of plant proteins by phosphorylation is a common signaling event that occurs in response to both abiotic and biotic stresses^30^. Protein kinase and protein phosphates play opposite functions in signal transduction. Many Protein kinase and protein phosphates were found to be PLSC phosphoproteins, involving in signal transduction metabolism in our study. Two phosphoproteins, CDPK8 (GSBRNA2T00081074001) and CDPK1 (GSBRNA2T00134334001) were identified as up-regulated PLSC phosphoproteins responding to drought stress in our study. CDPK can be activated to regulate kinase activity by phosphorylation. Ser40 and Thr65 of NtCDPK2 are phosphorylated response of hyperosmotic stress^31^. In our study, CDPK8 was phosphorylated at Thr39 and Ser37, and CDPK1 was phosphorylated at Ser90 and Ser568, respectively, indicating a relationship with stress response. Protein phosphatase 2 C, which are encoded by ABI gene involved in ABA signal transduction, can lead to the dephosphorylation of phosphoproteins. In our study, protein phosphatase 2 C 6 (GSBRNA2T00139037001) and protein phosphatase 2 C 10 (GSBRNA2T00110097001) had up-regulated phosphorylation under drought stress. In this study, other reported pathways of drought signaling include mitogen-activated protein kinase (MAPK or MPK) cascades, reactive oxygen species (ROS), BR-signaling kinase 3, serine/threonine-protein kinase were also identified as PLSC phosphoproteins, which were also used by rapeseed to trigger highly dynamic phosphorylation/dephosphorylation response to changing environment.

Sucrose phosphate synthase (SPS), and UDP-glucose 6-dehydrogenase were identified as PLSC phosphoproteins. SPS and UDP-glucose dehydrogenase are important enzymes in starch and sucrose metabolism pathway. SPS is a key regulatory enzyme in sucrose biosynthesis and has been linked to quantitative trait loci controlling plant growth, which is phosphorylated on multiple Ser residues^30^. UDP-glucose dehydrogenase plays an important role in cell wall pentose biosynthesis during the post-germinative growth of maize^32^. Here, SPS and UDP-glucose dehydrogenase were phosphorylated in response to drought stress, indicating that the enzymatic activity might be regulated by phosphorylation status and led to the altered growth. Taken together, these results suggested that drought stress induced a complex series of cellular responses, including phosphorylation of specific signaling components and metabolism pathway, which helped rapeseed adapt to drought stress.

### Proteins involved in photosynthesis and energy metabolism under drought stress

Photosynthesis, including photoreaction (light dependent) and dark reaction (carbon fixation), is usually depressed under drought stress in plants. Our results indicated that rapeseed could tolerate mild drought stress based on the measurement of chlorophyll and RWC content (Fig. 1). When the severe drought stress exceeded this boundary, the photosynthesis process was disturbed. In this study, we identified 31 2D-DIGE protein spots and 25 phosphoproteins involved in photosynthesis, and their abundance was significantly changed by drought treatment (Table S1, Supplemental Data 1). Rubisco is the key enzyme in the carbon fixation and mediates photosynthetic CO_2_ assimilation by catalyzing ribulose-1, 5-bisphosphate and CO_2_ to 3-phospho-glycerate^33^. The 10 Rubiscos (spots 422, 464, 1138, 1183, 1716, 1719, 1733, 1727, 1735, and 1740), also identified to undergo phosphorylation modification, were down-regulated under severe drought stress but showed no obvious changes under mild drought stress in 2D-DIGE, suggesting that the efficiency of CO_2_ fixation was inhibited under severe drought stress. Stomatal closure usually resulted in photosynthesis inhibition and strong reductions in the activity of Rubisco under most drought stress conditions^34^. In this study, our results revealed a negative effect of drought stress on Rubisco large and small subunits. Light-dependent reactions require four major protein complexes: PSII, cytochrome b6f complex, PSI, and ATP synthase. A total of 14 light reaction-related proteins were identified under drought stress including PSBO1, LFNR, chlorophyll *a/b* binding protein, and ATP synthase (Table S1). Among them, PSBO1 is also identified to undergo phosphorylation modification. In the present study, 3 PSBO1 proteins (spots 1117, 1125, and 1160) were identified as the PSII components. OEC catalyzes the oxidation of water to produce molecular oxygen, reduces plastoquinone, and generates a transmembrane proton gradient^35^. PSBO plays an important role in stabilizing the OEC^36^. In our study, 2 isoforms of PSBO1 proteins (spots 1125, 1160) were up-regulated under stress conditions to protect photosystem II against photodamage.

To provide energy for plant growth and in addition to cope with drought stress, large amounts of ATP are need. ATP synthase has been reported to be up-regulated in under stress conditions^9^,^35^,^37^. However, our proteomics results revealed some new patterns for ATP synthase. We noticed that 11 ATP synthase members were altered after drought treatment. Among them, 6 members (spots 147, 171, 349, 390, 435, 444, and 912) were up-regulated and 4 members (spots 363, 414, 447, and 453) were down-regulated. On the whole, the sum of ATP synthase increased under drought stress, suggesting that *B. napus* generated a higher demand for ATP to maintain homeostasis under the drought stress conditions to promote the ATP metabolic pathway and to protect *Brassica* plants from osmotic damage.

### Proteins involved in ROS scavenging were up-regulated upon drought stress

ROS are continuously produced as byproducts of various metabolic pathways, both under normal and stressful condition^38^. At physiologically low levels, ROS functions as signaling molecules in intracellular signaling and regulation, whereas excess ROS induce the oxidative modification of cellular macromolecules, inhibit protein function, and promote programmed cell death^39^. When plants are challenged by drought stress, ROS production is enhanced as a result of the inhibition of photosynthesis and predominance of photorespiration, triggering disturbances in normal cellular homeostasis, which are highly toxic^40^. Thus the balancing of production and detoxification of ROS is critical for maintaining cellular functions. Plants have evolved complex protective and regulatory mechanisms to prevent cellular injury by eliminating or reducing the cellular ROS levels under abiotic stress^41^. Usually, the transcription and translation of antioxidant enzymes involved in ROS scavenging are induced or up-regulated upon exposure to abiotic stress^42^. The results of our proteomics analysis revealed that the abundance levels of three antioxidative enzymes (APX1, APXT, and MDAR) significantly accumulated abundance under drought condition to regulate the ROS in the cell and protect cell against damage. Many glutathione metabolism-related proteins including GSTF9 (spot 1426), GSTU19 (spot 1379), GSTF8 (spot 1423), and GSTU5 (spot 1372) are involved in redox homeostasis. In our research, GSYU19, GSTF8, GSTU5, and GSTF9 were all up-regulated dramatically under drought stress and catalyzed the conjugation of glutathione to maintain cell redox homeostasis and protect organisms against oxidative stress. The results reflected the crucial roles of these proteins in ROS scavenging.

### Phosphorylation of βCΑ1 might be important for photosynthesis in response to drought stress

βCA1 was focused on because it involved in photosynthesis that is usually depressed under drought stress in plants. CA is a metalloenzyme located in the chloroplast stroma very close to Rubisco that catalyses the reversible reaction of bicarbonate to carbon dioxide and maintains the supply of CO_2_ for Rubisco activity^43^. In fact, there is a relatively constant ratio of CA to Rubisco transcript abundance and enzyme activity during plant development and changing environmental parameters^43^. In our study, CA activity decreased under drought stress (Fig. 5) and βCA1 showed a down-regulated phosphorylated site at Tyr207, which located in substrate-binding active region (Fig. 6). In addition, βCA and Rubisco showed similar expression patterns at both the transcriptional and translational levels (Fig. 3), which suggested that βCA could adjust its activity through phosphorylation modification ultimately coordinate photosynthesis processes to help plants adapt to drought stress.

The structure of the β−carbonic anhydrase from *Pisum sativum* was determined. The active site is located at the interface between two monomers, with Cys160, His220 and Cys223 binding the catalytic zinc ion and residues Asp162, Gly224, Gln151, Val184, Phe179 and Tyr205 interacting with the substrate analogue, acetic acid^44^. In our study, the three-dimensional structure analysis revealed that Cys162, His222 and Cys225 were zinc ligands (Fig.6). Additionally, Tyr207 was identified to have a phosphorylated site located in the Zn metal center (Fig. 6B and Table S5). In addition, Tyr207 was a bottleneck of the hydrophilic channel to gain access to the active site. Similar results were reported from *P. sativum* and *H. annuus*. This evidence led us to propose that phosphorylation of Tyr207 might be important for photosynthesis in response to drought stress. Protein phosphorylation greatly expand the diversity and function of proteome, resulting in intricate regulation of biological processes^6^. In this study, βCA1 was identified from eight protein spots, determined 15 phosphorylation sites upon drought stress (Table S4). The result suggested that phosphorylation of βCA1 could regulate photosynthesis process in response to drought stress. In addition, drought stress affected the CA activity, which globally affected CO_2_ concentration in chloroplast, which might result in the stomata in leaves closing in response to water deficit.

In summary, this study showed that photosynthesis was the most significant regulation pathway under drought stress. Phosphorylation of signal transduction, starch and sucrose metabolism helped rapeseed adapt to drought stress. The activity of βCA1 related to photosynthesis could be inhibited under drought stress. Protein phosphorylation of βCA1 might play an important role in regulating photosynthesis process in response to drought stress. The study provided a new clue to elucidate drought tolerance mechanism in *B.napus*.

## Materials and Methods

### Plant growth conditions and treatment

Three semi-winter cultivars of *B. napus* L. were used: 2AF009, 2AF410, 3DH020. Their seeds were collected from the National Mid-term Gene Bank for Oil Crops of China, germinated in petri dishes containing two layers of wet filter paper with deionized water and then incubated in a growth chamber under a long-day cycle (16-h-light/8-h-dark, 22 °C/20 °C) for 3 days. Following germination, seedlings were transferred to plastic pots containing a 1:1 mixture of vermiculite and nutrient soil moistened with distilled water. At the beginning of the experiment, pots were weighed to determine the soil water content before transferring seedlings.

Well grown 30-day-old seedlings were divided into three groups (the control groups, CK; mild drought stress group, MD; severe drought stress group, SD) for the drought treatments presented in Fig. 1A by controlling the available soil water capacity. Before drought treatment, the different cultivar seedlings and the pots were weighed to determine the soil water content and seedling weight. In the Excel worksheet file, a set of equations was used to calculate the soil water content in the cultivar pots, the required final water content, and the amount of water to be added. The pellets were weighed daily and were supplemented with the calculated amount of water to reach the required soil water content. The decrease in the soil water content was monitored and controlled at approximately 12% daily. When drought treatment was started by withholding water, we defined that as day 0 for drought stress. The treatment method as follows: (i) For the control group, plants were grown in well-watered conditions and the pots were provided with water daily to maintain the soil water content to approximately 90%. (ii) For mild drought stress, 30-day-old seedlings experienced a water deficit on day 3, 4, 5. Soil water content was changed from 90% to 60% and the soil water content was retained at 60% from day 5 to day 6. (iii) For severe drought stress, 30-day-old seedlings experienced a water deficit day 0–5. The soil water content was changed from 90% to 20% and the soil water content was retained at 20% from day 5 to day 6. Leaves used for the proteomics study were collected on the day 6 and immediately frozen in liquid nitrogen and stored at −80 °C for further study.

### Bioassays of plant seedlings under drought stress

Leaf water loss assay was carried out as described behind. Leaves were excised, weighed immediately, placed in clean plastic petri dishes, exposed to cold white light at 22 °C. Then, leaves were weighed at different time points to determine plant fresh weight at different time points. The percentage of fresh weight loss versus initial weight was used to represent water loss. RWC was measured according to the published method^10^. After samples were collected, leaves fresh weight (FW) was measured immediately, rehydrated in water for 24 h until fully turgid, then weighed leaves turgid weight (TW). After that, samples were dried in an oven at 60 °C for 48 h and reweighed dry weight (DW). The RWC was calculated by the following formula: RWC (%) = (FW-DW)/(TW-DW)*100. Proline content was determined as described elsewhere^45^. L-proline standards served as the control. Proline was calculated according to the following formula: proline (μg/g) = (C * V)/W. C represents proline concentration. V represents the total volume. W represents the leaves fresh weight. Photosynthetic pigments were estimated as described elsewhere^46^. The absorbance is determined at 663 nm and 645 nm for chlorophyll *a* and chlorophyll *b* measurement, respectively. Photosynthetic pigments were calculated according to the following formula: chlorophyll *a* (mg/g) = (12.7 * A663–2.69 * A645) * 12.5/W, chlorophyll *b* (mg/g) = (22.9 * A645–4.68 * A663) * 12.5/W. Three biological replicates were performed for all drought treatments.

To assess stomata in response to drought stress, the light-induced stomatal opening was analyzed after 2.5 h in the light. Leaf abaxial epidermis were peeled off using sharp forceps, transferred into a drop of floating solution on a glass slide, immediately observed under Olympus IX71 microscope (Olympus America, Melville, NY). Pictures were taken in random regions.

### Protein extraction, 2D-DIGE, and image analysis

Total leaf protein was extracted as described elsewhere^46^. Protein concentration was determined by the Bradford assay using a UV-160 spectrophotometer (Shimadzu, Kyoto, Japan) and bovine serum albumin as the protein standard^47^.

For 2D-DIGE, proteins were labeled with CyDye DIGE Fluor dye (GE Healthcare, Uppsala, Sweden) according to the manufacturer’s instructions. After adjusting protein samples pH to 8.5 using 10 mM Tris buffer, aliquot of 50 μg proteins were mixed with 250 pmol of CyDye, incubated on ice under dark condition for 45 min. The reaction was terminated by addition of 1 μL of 10 mM lysine on ice for 10 min. The Cy2-, Cy3-, and Cy5-labeled protein samples were mixed together. A total of 150 μg proteins were added into lysis buffer to adjust the final volume to 455 μL. Samples were loaded onto an IPG strips with linear PH gradient 4–7 and 24 cm length (GE Healthcare, Uppsala, Sweden). After hydrated, the strips were performed isoelectric focusing (IEF) and SDS-PAGE gels electrophoresis to separate proteins as described^48^.

The gels were scanned using a Typhoon Trio scanner (GE Healthcare, USA) at 100 mm (pixel size) resolution, and the DIGE images were analyzed using DeCyder V7.0 software as described in the user manual (GE Healthcare, USA). Differential in gel analysis module was used for protein spot detection. Biological variation analysis module was used to match protein spots present in three replicates. The threshold of 1.2 fold change was determined by the DeCyder V7.0 software based on our experiments. The significant change of each spot is different in DIGE images by DeCyder as described in the user manual (GE Healthcare, USA). For 2D-DIGE analysis, normalisation factors were determined based on total spot volume and the matched spot volumes were normalised across whole match set. Normalised spot volumes were compared between images in match set (if data was identical, all ratios = 1, for different data, ratios were > or <1). Difference thresholds were applied based on normalised volume of spot. Ratios exceeding threshold limits were highlighted as spot differences between samples. So, those protein spots showing a ratio of intensity of greater than 1.2 fold and a P-value of less than 0.05 were considered to be differentially abundant protein spots due to DIGE can detect protein differences as low as 1.2-fold change^23^.

### Protein identification *via* MALDI TOF/TOF MS

DAPs were excised manually and digested in-gel with bovine trypsin (Roche, Cat. 11418025001) as described elsewhere^49^. The digested peptides were mixed with α-cyano-4-hydroxycinnamic acid (CHCA) and analyzed by AB SCIEX MALDI TOF-TOF 5800 System (AB SCIEX, Foster City, CA, USA). *B.napus* genome sequences were downloaded from CoGe database (https://genomevolution.org/CoGe/). *B.napus* proteins sequences was released in August 2014 (101040 sequences, version 5.0). The local protein database was built using MASCOT containing 101040 protein sequences derived from the complete genome sequences of *B.napus*. MS peptides search the *B.napus* local database using ProteinPilot Software. When individual ions scores were higher than the threshold score (>62), proteins were considered as a confident identification or with an extensive homology (P < 0.05). At least 2 peptides matched the observed masses for an identification to be considered valid. The positive matches were BLASTP searched against the uniProt protein database for updated annotation and identification of homologous proteins^18^.

### Quantitative RT-PCR analysis

Total RNA was isolated from frozen *B.napus* leaves using TRIZOL reagent (Invitrogen, CA, USA). First-strand cDNA was synthesized using reverse transcriptase kit reagents (Thermo, Tokyo, Japan) according to the manufacturer’s instruction. Approximately 1 μg of RNA was used for the reverse transcription. Primer pairs used for quantitative real-time PCR (qRT-PCR) were provided in supplemental Table S5. The specificity of the primers were checked by observing the melting curve of the quantitative RT-PCR products and the specific band on the agarose gel. The actin gene was used as an internal reference for normalization as follows: sense, 5-CTGGAATTGCTGACCGTATGAG-3; antisense, 5-ATCTGTTGGAAAGTGCTGAGGG-3. Reaction was conducted on an Mx3500 P Real-Time PCR system (Stratagene, NSW, Australia) according to the manufacturer’s instruction. All data were analyzed using the MxPro software (Stratagene).

### Phosphopeptide identification and phosphorylation site determination

Extracted protein mixtures were directly reduced with DTT, subsequently alkylated with iodoacetamide, digested with trypsin. Phosphopeptides were enriched by Titansphere Phos-Tio Kit (GL Science, Tokyo, Japan) following the manufacturer’s instruction^50^. Enriched phosphopeptides were dried using a centrifugal vacuum concentrator. The samples were desalted using C18 Zip-tip (Thermo Pierce, Rockford) according to manufacturer’s instructions, redried using a centrifugal vacuum concentrator. Data was analyzed by nanospray ESI-MS performed on a Thermo Q-Exactive high resolution mass spectrometer (Thermo Scientific, Waltham, MA, USA). The 10 most intensive peptide signals from the full scan were selected for MS/MS scan.

Mascot coupled with Scaffold software was employed to determine probability of the phosphorylation site location. Mass spectra were searched against *B.napus* local database using Mascot 2.4 search engine. Search results were loaded to Scaffold 4.4.6 software (Portland, OR, USA) to validate MS/MS peptides and protein identifications. Identifications were accepted if they had a greater than 95% peptide probability and contained at least one identifiable phosphopeptides. Scaffold PTM (Version 2.2) was used to annotate phosphorylation site located in MS/MS spectra. Ascore was used to assess phosphorylation site probability. Only the phosphopeptides with Ascore localization probability minimum of 95% were considered as high probability.

### Label free quantitation

A label-free quantification (LFQ) algorithm was used to compare phosphopeptides abundances between different samples^25^. The SI_N_ was calculated by using MASCOT search results. Briefly, for each protein we calculated the spectral index (SI) which incorporated fragment ion intensity values with spectrum count and peptide number. SI was then normalized relative to the sum of SI of each identified protein in the experiment, and then further normalized by protein length. The fragment ion intensity of the spectrum assigned to a specific peptide was obtained from the MS_2_ section in MASCOT search results. The spectra for SIN were selected from the spectrum list with FDR <1%. On the basis of analyses of three biological replicated, only phosphopeptides with a log_2_ fold change (drought stress/CK LFQ intensity, log_2_FC) ≥ 1 or ≤ −1 (p < 0.05) were considered to be significantly altered at the phosphorylation level.

### Protein functional classifications and hierarchical clustering analysis

SOTA (Self-Organizing Tree Algorithm) hierarchical clustering of the expression profiles was performed using open source Cluster software 3.0^51^. GO annotation and enrichment analysis were performed using AgBase (http://www.agbase.msstate.edu) and AgriGO database (http://bioinfo.cau.edu.cn/agriGO/). Subcellular localization was predicted using CELLO V.2.5 (http://cello.life.nctu.edu.tw). Identified proteins were further analyzed using the STRING V10.0 database (http://string-db.org/) for PPI network analysis, to statistically determine the functions and pathways most strongly associated with the protein list^48^. KEGG (http://www.genome.jp/kegg/pathway) pathway analysis was performed to determine their molecular interaction and reaction networks^52^. 3D structure modeling was built using SWISS-MODEL (http://swissmodel.expasy.org/), visualized by PyMOL software (http://www.pymol.org). In addition, protein phosphorylation modification was visualized by Discovery Studio 2.5 software package (Accelrys, Inc.)^53^.

### Measurement of carbonic anhydrase activity

Approximately 0.5 g of rapeseed leaves were ground in liquid nitrogen and immediately resuspended in 3 mL of extraction buffer (10 mM barbital sodium, 50 mM β-mercaptoethanol, pH 8.3). The lysate was cleared by centrifugation at 15,400 g for 5 min at 4 °C. CA activity was measured by the pH method with some modifications. Approximately 500 μL of enzyme extracting solution was added to 5 mL of 20 mM barbital sodium (pH 8.3) maintained at 4 °C. Addition of 4.5 mL of ice-cold CO_2_-saturated water initiated the reaction and the time required for the pH change from 8.3–7.3 was measured.

### Statistical analysis

For physiological and biochemical data, at least three biological replicates were analysed for each drought treatment. The statistical significance (P values) in mean values was determined with unpaired two-tailed Student’s t-test (Microsoft Excel). The significance level of P < 0.05 was considered to indicate statistical significance. Statistical analysis was performed using SPSS software. For protein abundance analysis, one-way analysis of variance (ANOVA) was performed at a 5% significance level and differentially expressed proteins with an absolute ratio of at least 1.2-fold were selected. A principle component analysis (PCA) was run on the 4000 proteins matched on every gel and on the different spot maps for qualitative appreciation of the proteomic results. ANOVA and PCA were performed on Decyder Extended Data Analysis.

## Additional Information

**How to cite this article**: Wang, L. *et al*. Comparative Proteomics Reveals that Phosphorylation of β Carbonic Anhydrase 1 Might be Important for Adaptation to Drought Stress in *Brassica napus. Sci. Rep.* **6**, 39024; doi: 10.1038/srep39024 (2016).

**Publisher's note:** Springer Nature remains neutral with regard to jurisdictional claims in published maps and institutional affiliations.

## Supplementary Material

Supplementary Information

Supplementary Dataset 1

Supplementary Dataset 2

## Figures and Tables

**Table 1:** DAPs that were also identified as phosphoproteins with significantly changed phosphorylated sites.

DEP spot ID	Protein accession number	Description	Phosphorylated peptide and site
1588	GSBRNA2T00008050001	Unknown protein	SSSLVVSS**s**PKPVR
1727, 1735	GSBRNA2T00011400001	Ribulose bisphosphate carboxylase	QVQCISFIAYKPPSF**t**GA
1740		small chain 1B	
516, 519	GSBRNA2T00025997001	Translational inititation factor 4A-1	VHACVGG**t**SVR
464, 1138	GSBRNA2T00015407001	Ribulose bisphosphate carboxylase	LSGGDHVHAG**t**VVGK
		large chain	
1327,1328,1331,1339,1343,1360,1361,1370	gi|297789439	Beta carbonic anhydrase 1	YGGVGAAIE**y**AVLHLK
729,734	GSBRNA2T00097384001	Phosphoglycerate kinase	ELDYLVGAVS**s**PK
120,124	GSBRNA2T07253900001	Transketolase-1	ALPTYTPE**s**PGDATR
1294	GSBRNA2T00035007001	Chlorophyll a-b binding protein	KtVKPTGPSGSPWYGSDR
739, 741	GSBRNA2T00155196001	Ribulose bisphosphate	GLAYD**t**SDDQQDITR
813, 1357		carboxylase/oxygenase activase	
