# Supplementary material for: Comparative Proteomics Reveals that Phosphorylation of β Carbonic Anhydrase 1 Might be Important for Adaptation to Drought Stress in Brassica napus
Source: Sci Rep. 2016 Dec 14;6:39024. doi: 10.1038/srep39024 (PMC5155245; doi:10.1038/srep39024)
Supplement: Supplementary Information [file srep39024-s1.doc]

**Supplemental Information**

**Title: Comparative Proteomics Reveals that Phosphorylation of** **β Carbonic Anhydrase 1 is Important for Adaptation to Drought Stress in *Brassica napus***

**Authors:**

**Limin Wang**

1. Oil Crops Research Institute of the Chinese Academy of Agricultural Sciences, Key Laboratory of Biology and Genetic Improvement of Oil Crops, Ministry of Agriculture, Wuhan 430062, China

2. National Key Lab of Crop Genetic Improvement, College of Plant Science and Technology, National Center of Crop Molecular Breeding, National Center of Oil Crop Improvement, Huazhong Agricultural University, Wuhan 430070, China

**Xiang Jin**

Institute of Tropical Biosciences and Biotechnology, Chinese Academy of Tropical Agricultural Sciences, Haikou, Hainan 571101, China

**Qingbin Li**

State Key Laboratory of Agrobiotechnology, College of Biological Sciences, China Agricultural University, Beijing 100193, China

**Xuchu Wang (Corresponding author)**

Institute of Tropical Biosciences and Biotechnology, Chinese Academy of Tropical Agricultural Sciences, Haikou, Hainan 571101, China

E-mail address: [xchwanghainan@163.com](mailto:xchwanghainan@163.com)

**Zaiyun Li (Corresponding author)**

National Key Lab of Crop Genetic Improvement, College of Plant Science and Technology, National Center of Crop Molecular Breeding, National Center of Oil Crop Improvement, Huazhong Agricultural University, Wuhan 430070, China

E-mail address: lizaiyun@mail.hzau.edu.cn

**Xiaoming Wu (Corresponding author)**

Oil Crops Research Institute of the Chinese Academy of Agricultural Sciences, Key Laboratory of Biology and Genetic Improvement of Oil Crops, Ministry of Agriculture, Wuhan 430062, China

E-mail address: [wuxm@oilcrops.cn](mailto:wuxm@oilcrops.cn)

**Supplemental Figures and Tables**

**Supplemental Fig. S1.** Morphological patterns of representative 2AF009, 3DH020, and 2AF410 plants under CK, MD, and SD conditions.

**Supplemental Fig. S2.** Detail information for protein identification with MALDI TOF MS.

**Supplemental Fig. S3.** Classification and functional analysis of the DEPs under drought stress.

**Supplemental Fig. S4.** The main pathways.

**Supplemental Table S1.** Information of the identified differential expression protein spots.

**Supplemental Table S2.** DEPs identified from two to eight DEP spots on 2D-DIGE maps.

**Supplemental Table S3.** Information of PPI networks.

**Supplemental Table S4.** Phosphorylation sites of the eight BCA1 protein spots from 2D gel.

**Supplemental Table S5.** Primer sequences used for qRT-PCR analysis.

**Supplemental Figure S1**

Morphological patterns of representative 2AF009, 3DH020, and 2AF410 plants under CK, MD, and SD conditions.


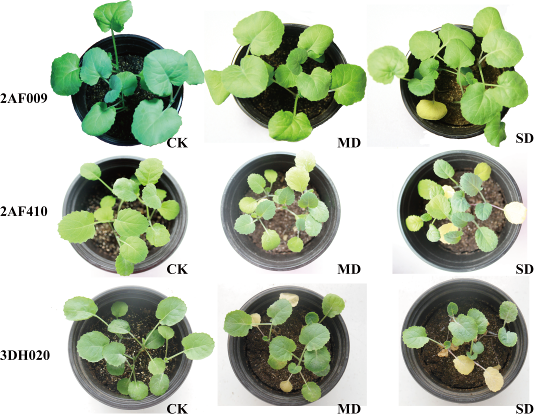


**Supplemental Figure S2**

Supplemental spectra and MALDI TOF/TOF MS/MS identification information for the drought responsive rapeseed proteins

Annotated spectra 138 differential expression proteins on DIGE gels identified by PMF and the combination results of PMF and PFF.

**CID:** collision induced dissociation;

**MALDI TOF**:

matrix assisted laser desorption/ionization time of flight;

**MS**: mass spectrometry;

PMF: peptide mass fingerprinting;

**PFF**: peptide fragment fingerprinting.

Spot No.: **120**

NCBI accession No.: **GSBRNA2T00072539001** Species: *Brassica napus*

PFF score: **107**

Protein name: **Transketolase-1**

Matched peptides No.: **3**  Sequence coverage %: **9**

Calculated Mr: **79864**  Calculated *p*I: **5.90**

Probability Based Mow


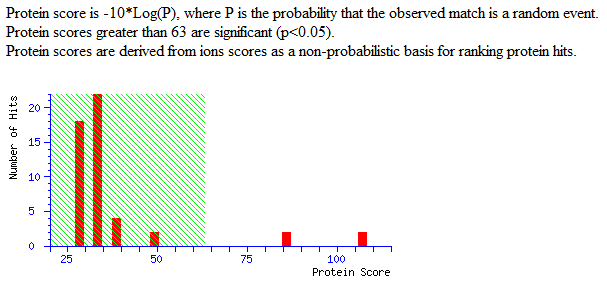


Matched peptide sequences: shown in Bold Red


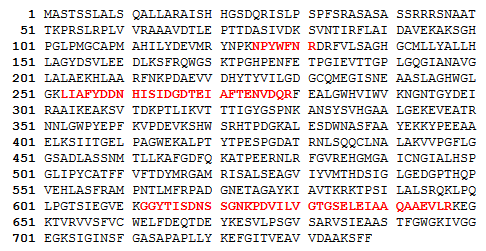


Matched peptides information:


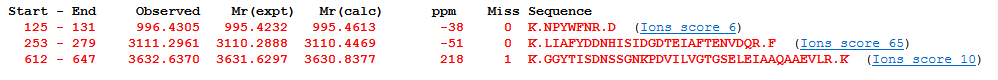


Spot No.: **124**

NCBI accession No.: [**GSBRNA2T00149203001**](http://massboss/mascot/cgi/protein_view.pl?file=../data/20150311/F012636.dat&hit=1) Species: *Brassica napus*

PFF score: **85**

Protein name: **Transketolase-1**

Matched peptides No.: **4**  Sequence coverage %: **11**

Calculated Mr: **79678**  Calculated *p*I: **6.00**

Probability Based Mow


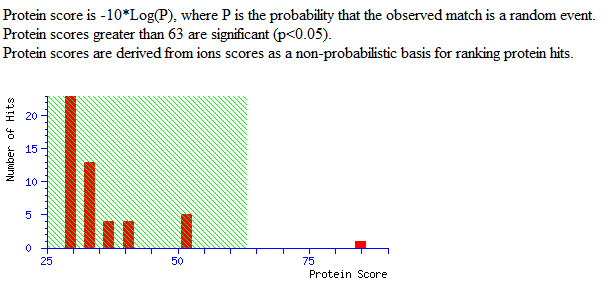


Matched peptide sequences: shown in Bold Red


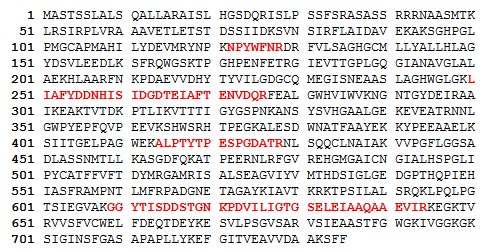


Matched peptides information:


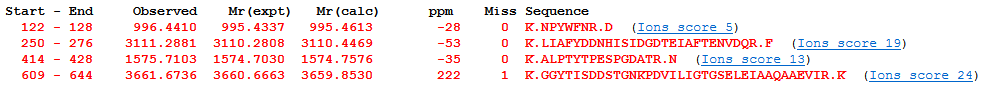


Spot No.: **127**

NCBI accession No.: **GSBRNA2T00050483001** Species: *Brassica napus*

PFF score: **92**

Protein name: **Mediator of RNA polymerase II transcription subunit 37e**

Matched peptides No.: **4** Sequence coverage %: **9**

Calculated Mr: **46492**  Calculated *p*I: **5.59**

Probability Based Mow


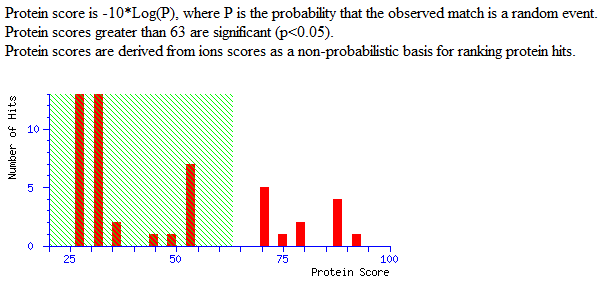


Matched peptide sequences: shown in Bold Red


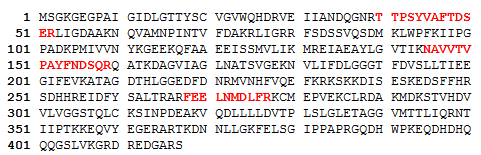


Matched peptides information:


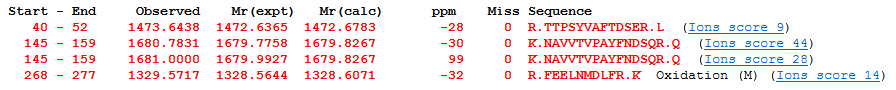


Spot No.: **139**

NCBI accession No.: **[GSBRNA2T00000934001](http://massboss/mascot/cgi/master_results.pl?file=../data/20150311/F012639.dat&REPTYPE=protein#Hit1)**  Species: *Brassica napus*

PFF score: **454**

Protein name: **Heat shock 70 kDa protein 7**

Matched peptides No.: **9** Sequence coverage %: **18**

Calculated Mr: **66871**  Calculated *p*I: **4.85**

Probability Based Mow


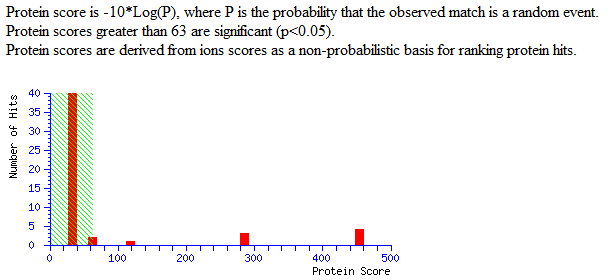


Matched peptide sequences: shown in Bold Red


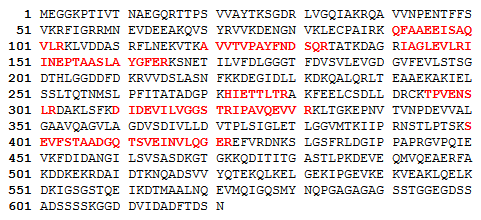


Matched peptides information:


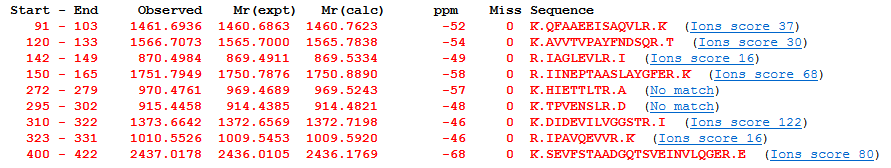


Spot No.: **147**

NCBI accession No.: **[GSBRNA2T00015387001](http://massboss/mascot/cgi/master_results.pl?file=../data/20150324/F016325.dat&REPTYPE=protein#Hit1)** Species: *Brassica napus*

PFF score: **277**

Protein name: **ATP synthase subunit alpha**

Matched peptides No.: **6**  Sequence coverage %: **26**

Calculated Mr: **33793**  Calculated *p*I: **5.09**

Probability Based Mow


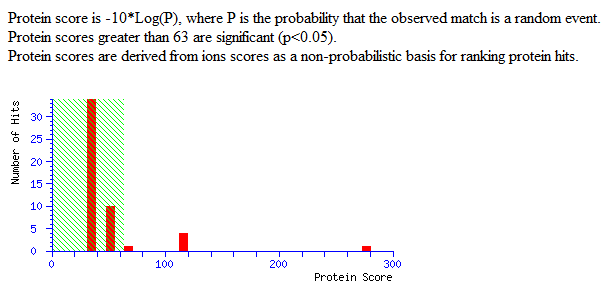


Matched peptide sequences: shown in Bold Red


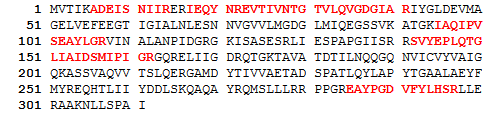


Matched peptides information:


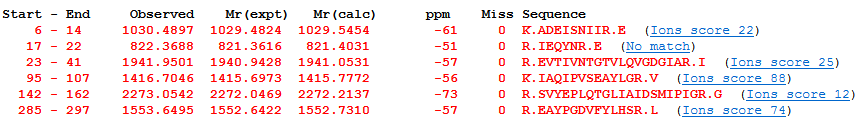


Spot No.: **150**

NCBI accession No.: **[GSBRNA2T00007549001](http://massboss/mascot/cgi/master_results.pl?file=../data/20150328/F016463.dat&REPTYPE=protein#Hit1)** Species: *Brassica napus*

PFF score: **136**

Protein name: **Mediator of RNA polymerase II transcription subunit 37e**

Matched peptides No.: **5** Sequence coverage %: **8**

Calculated Mr: **71432**  Calculated *p*I: **5.14**

Probability Based Mow


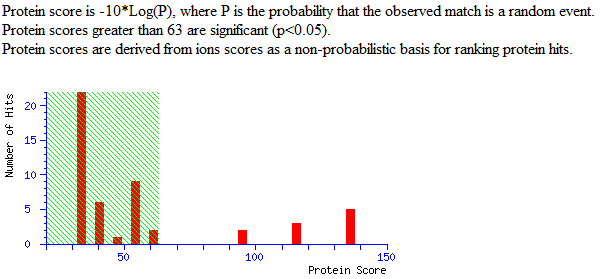


Matched peptide sequences: shown in Bold Red


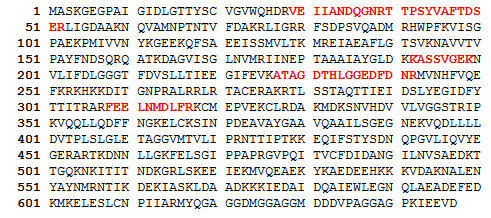


Matched peptides information:


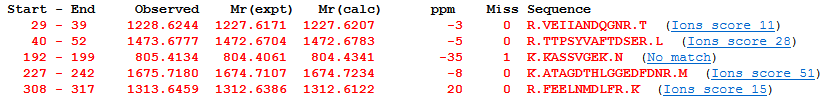


Spot No.: **156**

NCBI accession No.: **[GSBRNA2T00113654001](http://massboss/mascot/cgi/master_results.pl?file=../data/20150324/F016326.dat&REPTYPE=protein#Hit1)** Species: *Brassica napus*

PFF score: **122**

Protein name: **Mediator of RNA polymerase II transcription subunit 37c**

Matched peptides No.: **5**  Sequence coverage %: **15**

Calculated Mr: **20128**  Calculated *p*I: **7.77**

Probability Based Mow


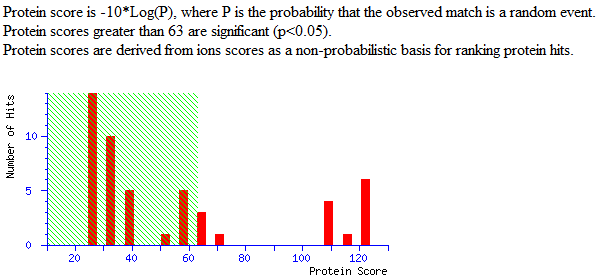


Matched peptide sequences: shown in Bold Red


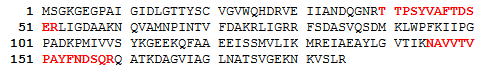


Matched peptides information:


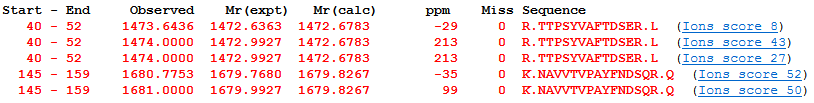


Spot No.: **163**

NCBI accession No.: **[GSBRNA2T00113654001](http://massboss/mascot/cgi/master_results.pl?file=../data/20150311/F012643.dat&REPTYPE=protein#Hit1)** Species: *Brassica napus*

PFF score: **144**

Protein name: **Mediator of RNA polymerase II transcription subunit 37e**

Matched peptides No.: **3** Sequence coverage %: **15**

Calculated Mr: **20128**  Calculated *p*I: **7.77**

Probability Based Mow


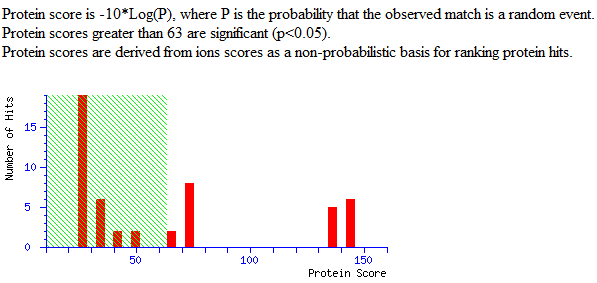


Matched peptide sequences: shown in Bold Red


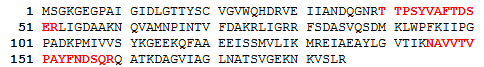


Matched peptides information:


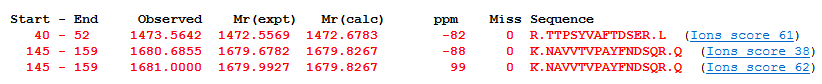


Spot No.: **164**

NCBI accession No.: **[GSBRNA2T00007549001](http://massboss/mascot/cgi/master_results.pl?file=../data/20150311/F012644.dat&REPTYPE=protein#Hit1)** Species: *Brassica napus*

PFF score: **134**

Protein name: **Mediator of RNA polymerase II transcription subunit 37c**

Matched peptides No.: **5**  Sequence coverage %: **7**

Calculated Mr: **71425**  Calculated *p*I: **5.14**

Probability Based Mow


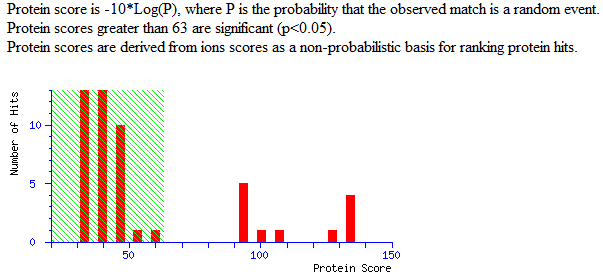


Matched peptide sequences: shown in Bold Red


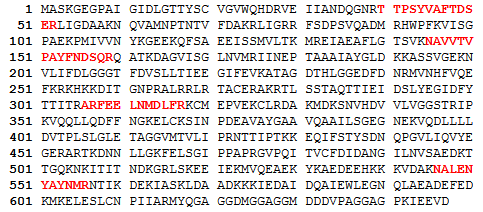


Matched peptides information:


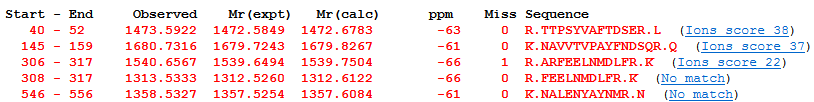


Spot No.: **171**

NCBI accession No.: **[GSBRNA2T00022872001](http://massboss/mascot/cgi/master_results.pl?file=../data/20150311/F012645.dat&REPTYPE=protein#Hit1)**  Species: *Brassica napus*

PFF score: **560**

Protein name: **V-type proton ATPase catalytic subunit A**

Matched peptides No.: **18**  Sequence coverage %: **28**

Calculated Mr: **68969**  Calculated *p*I: **5.16**

Probability Based Mow


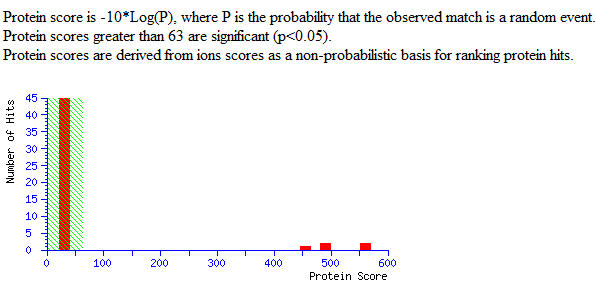


Matched peptide sequences: shown in Bold Red


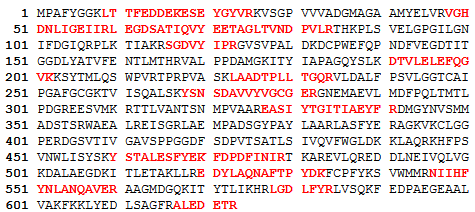


Matched peptides information:


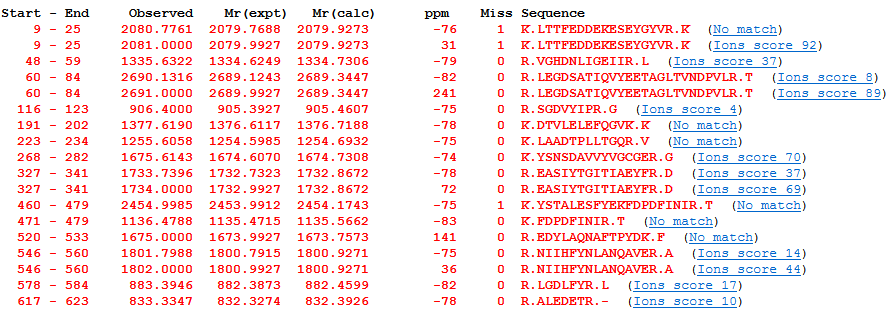


Spot No.: **209**

NCBI accession No.: **[GSBRNA2T00048583001](http://massboss/mascot/cgi/master_results.pl?file=../data/20150328/F016464.dat&REPTYPE=protein#Hit1)**Species: *Brassica napus*

PFF score: **114**

Protein name: **ATP-dependent zinc metalloprotease FTSH 2**

Matched peptides No.: **6** Sequence coverage %: **9**

Calculated Mr: **76637**  Calculated *p*I: **5.74**

Probability Based Mow


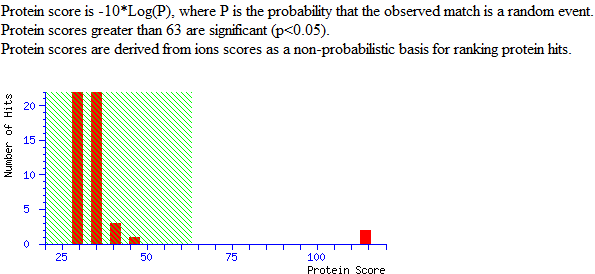


Matched peptide sequences: shown in Bold Red


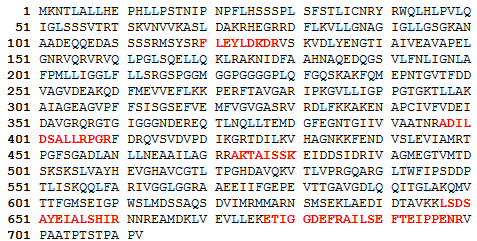


Matched peptides information:


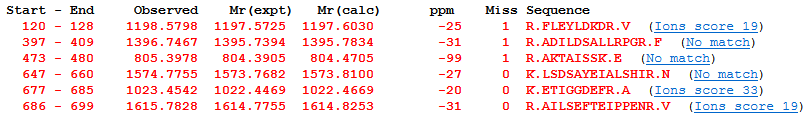


Spot No.: **210**

NCBI accession No.: **[GSBRNA2T00048583001](http://massboss/mascot/cgi/master_results.pl?file=../data/20150311/F012647.dat&REPTYPE=protein#Hit1)**  Species: *Brassica napus*

PFF score: **423**

Protein name: **ATP-dependent zinc metalloprotease FTSH 2**

Matched peptides No.: **7**  Sequence coverage %: **14**

Calculated Mr: **76634**  Calculated *p*I: **5.74**

Probability Based Mow


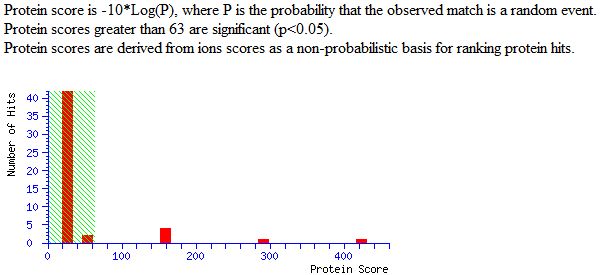


Matched peptide sequences: shown in Bold Red


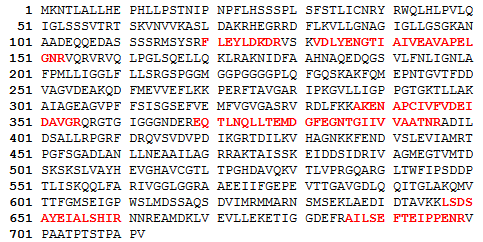


Matched peptides information:


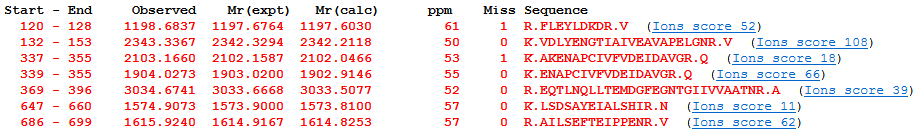


Spot No.: **251**

NCBI accession No.: **[GSBRNA2T00138831001](http://massboss/mascot/cgi/master_results.pl?file=../data/20150311/F012652.dat&REPTYPE=protein#Hit1)** Species: *Brassica napus*

PFF score: **254**

Protein name: **Chaperonin 60 subunit alpha 1**

Matched peptides No.: **6** Sequence coverage %: **13**

Calculated Mr: **61569**  Calculated *p*I: **5.02**

Probability Based Mow


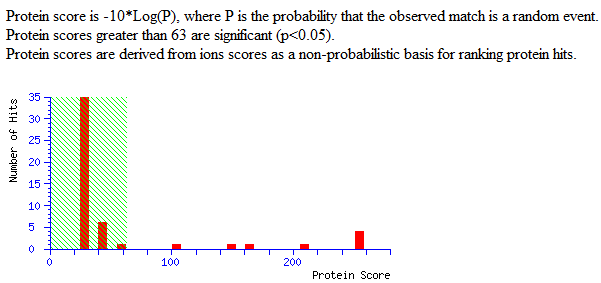


Matched peptide sequences: shown in Bold Red


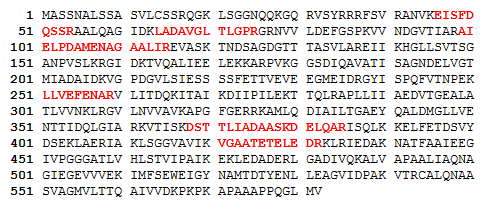


Matched peptides information:


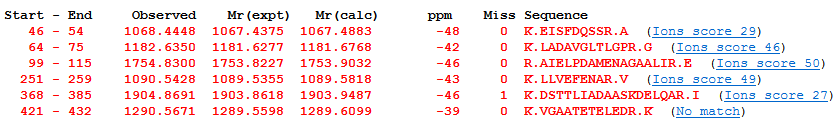


Spot No.: **269**

NCBI accession No.: **[GSBRNA2T00123626001](http://massboss/mascot/cgi/master_results.pl?file=../data/20150328/F016466.dat&REPTYPE=protein#Hit1)** Species: *Brassica napus*

PFF score: **145**

Protein name: **Chaperonin 60 subunit beta 2**

Matched peptides No.: **8**  Sequence coverage %: **16**

Calculated Mr: **63277**  Calculated *p*I: **6.29**

Probability Based Mow


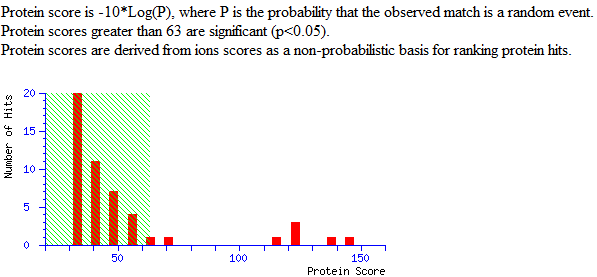


Matched peptide sequences: shown in Bold Red


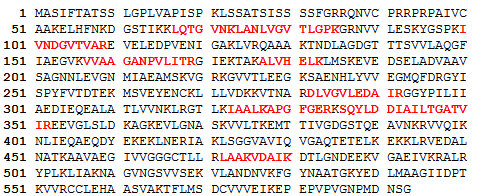


Matched peptides information:


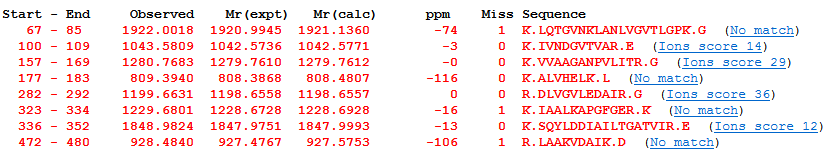


Spot No.: **274**

NCBI accession No.: **[GSBRNA2T00045232001](http://massboss/mascot/cgi/master_results.pl?file=../data/20150328/F016467.dat&REPTYPE=protein#Hit1)** Species: *Brassica napus*

PFF score: **324**

Protein name: **Chaperonin 60 subunit alpha 1**

Matched peptides No.: **9** Sequence coverage %: **18**

Calculated Mr: **61912**  Calculated *p*I: **5.15**

Probability Based Mow


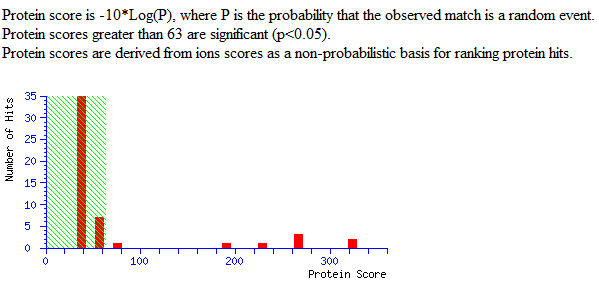


Matched peptide sequences: shown in Bold Red


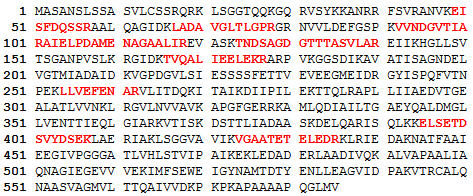


Matched peptides information:


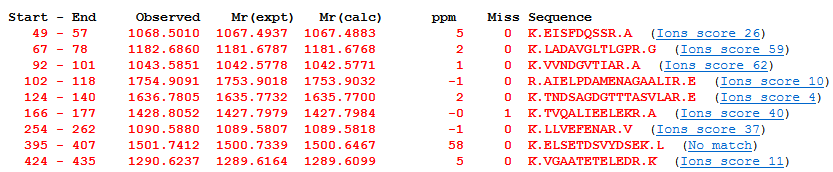


Spot No.: **277**

NCBI accession No.: **[GSBRNA2T00028808001](http://massboss/mascot/cgi/master_results.pl?file=../data/20150311/F012655.dat&REPTYPE=protein#Hit1)**  Species: *Brassica napus*

PFF score: **72**

Protein name: **unknown**

Matched peptides No.: **4**  Sequence coverage %: **15**

Calculated Mr: **39504** Calculated *p*I: **8.30**

Probability Based Mow


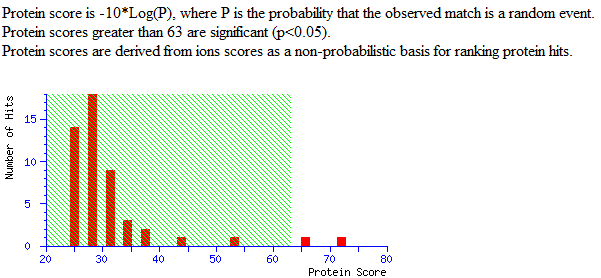


Matched peptide sequences: shown in Bold Red


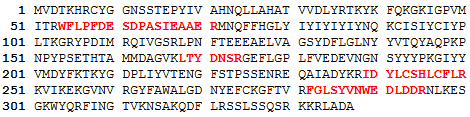


Matched peptides information:


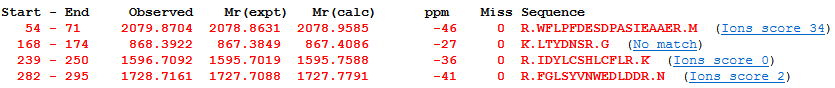


Spot No.: **282**

NCBI accession No.: **[GSBRNA2T00004725001](http://massboss/mascot/cgi/master_results.pl?file=../data/20150324/F016333.dat&REPTYPE=protein#Hit1)** Species: *Brassica napus*

PFF score: **68**

Protein name: **unknown**

Matched peptides No.: **3**   Sequence coverage %: **5**

Calculated Mr: **61515**  Calculated *p*I: **8.86**

Probability Based Mow


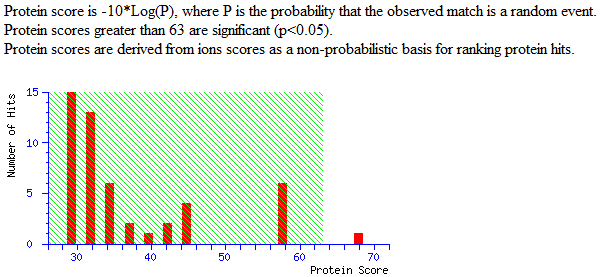


Matched peptide sequences: shown in Bold Red


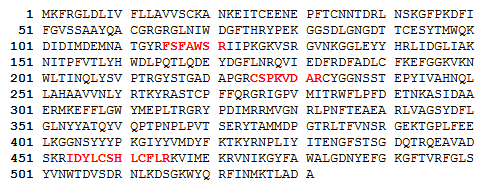


Matched peptides information:


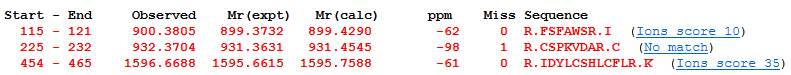


Spot No.: **288**

NCBI accession No.: **[GSBRNA2T00118698001](http://massboss/mascot/cgi/master_results.pl?file=../data/20150311/F012658.dat&REPTYPE=protein#Hit1)** Species: *Brassica napus*

PFF score: **301**

Protein name: **Chaperonin 60 subunit alpha 1**

Matched peptides No.: **6** Sequence coverage %: **12**

Calculated Mr: **63340** Calculated *p*I: **5.06**

Probability Based Mow


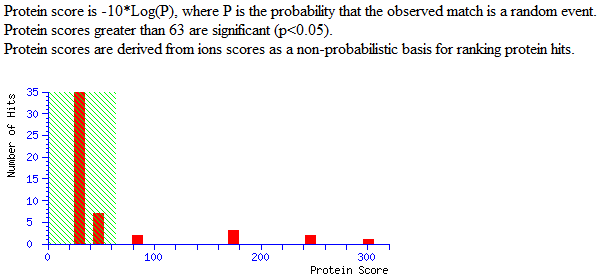


Matched peptide sequences: shown in Bold Red


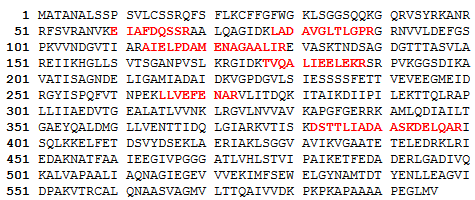


Matched peptides information:


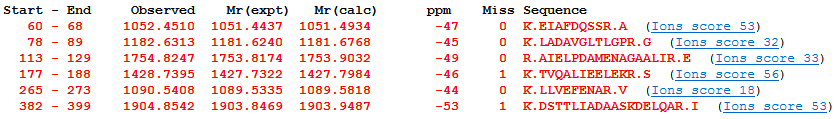


Spot No.: **297**

NCBI accession No.: **[GSBRNA2T00066638001](http://massboss/mascot/cgi/master_results.pl?file=../data/20150311/F012660.dat&REPTYPE=protein#Hit1)** Species: *Brassica napus*

PFF score: **106**

Protein name: **Protein disulfide isomerase-like 1-1**

Matched peptides No.: **4** Sequence coverage %: **6**

Calculated Mr: **55919**  Calculated *p*I: **4.82**

Probability Based Mow


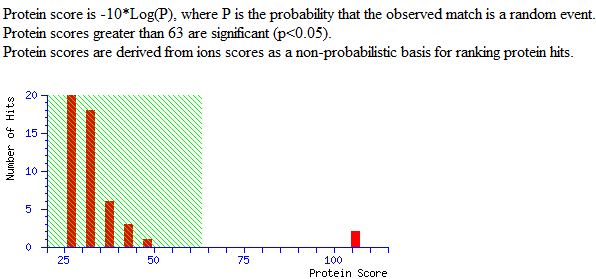


Matched peptide sequences: shown in Bold Red


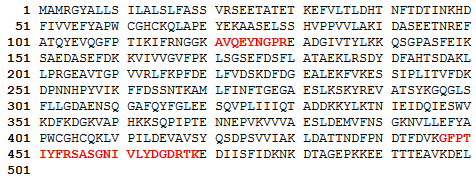


Matched peptides information:


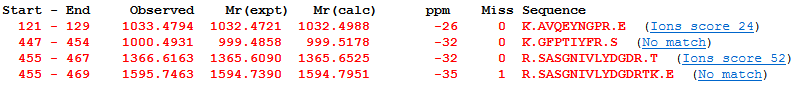


Spot No.:**310**

NCBI accession No.: **[GSBRNA2T00034728001](http://massboss/mascot/cgi/master_results.pl?file=../data/20150328/F016473.dat&REPTYPE=protein#Hit1)**  Species: *Brassica napus*

PFF score: **151**

Protein name: **Chaperonin 60 subunit beta 1**

Matched peptides No.: **7**  Sequence coverage %: **12**

Calculated Mr: **67367**  Calculated *p*I: **6.99**

Probability Based Mow


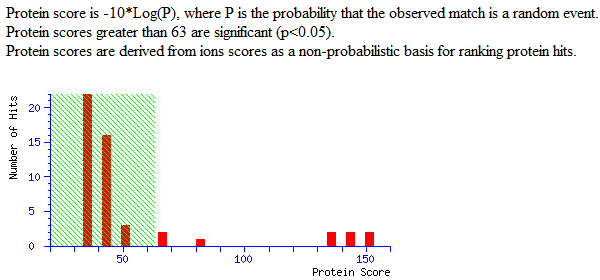


Matched peptide sequences: shown in Bold Red


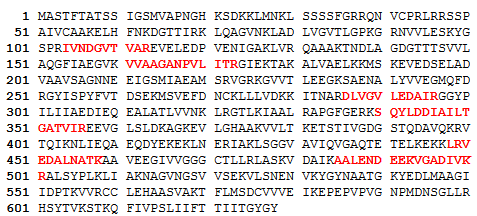


Matched peptides information:


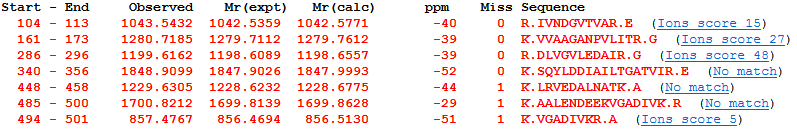


Spot No.:**311**

NCBI accession No.: **[GSBRNA2T00125571001](http://massboss/mascot/cgi/master_results.pl?file=../data/20150311/F012662.dat&REPTYPE=protein#Hit1)**  Species: *Brassica napus*

PFF score: **270**

Protein name: **Chaperonin 60 subunit beta 1**

Matched peptides No.: **7** Sequence coverage %: **9**

Calculated Mr: **64232**  Calculated *p*I: **6.10**

Probability Based Mow


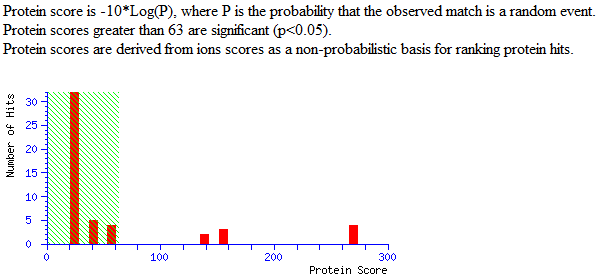


Matched peptide sequences: shown in Bold Red


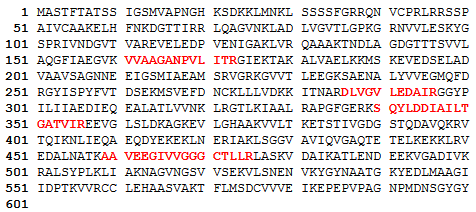


Matched peptides information:


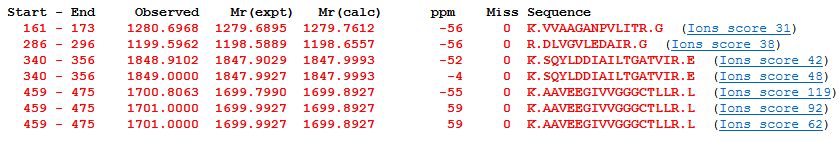


Spot No.: **315**

NCBI accession No.: **[GSBRNA2T00114056001](http://massboss/mascot/cgi/master_results.pl?file=../data/20150311/F012663.dat&REPTYPE=protein#Hit1)** Species: *Brassica napus*

PFF score: **226**

Protein name: **Chaperonin 60 subunit beta 1**

Matched peptides No.: **9** Sequence coverage %: **15**

Calculated Mr: **75353**  Calculated *p*I: **5.54**

Probability Based Mow


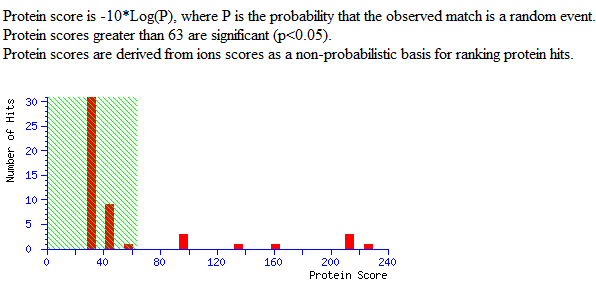


Matched peptide sequences: shown in Bold Red


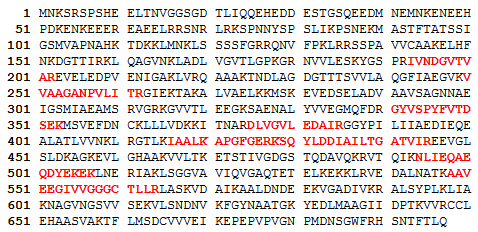


Matched peptides information:


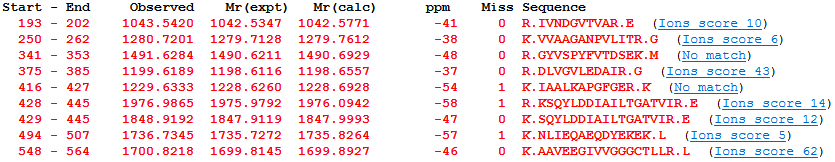


Spot No.: **349**

NCBI accession No.: **[GSBRNA2T00015387001](http://massboss/mascot/cgi/master_results.pl?file=../data/20150311/F012665.dat&REPTYPE=protein#Hit1)** Species: *Brassica napus*

PFF score: **464**

Protein name: **ATP synthase subunit alpha**

Matched peptides No.: **9**  Sequence coverage %: **36**

Calculated Mr: **33793** Calculated *p*I: **5.09**

Probability Based Mow


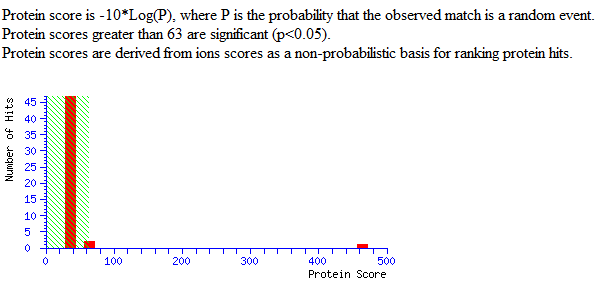


Matched peptide sequences: shown in Bold Red


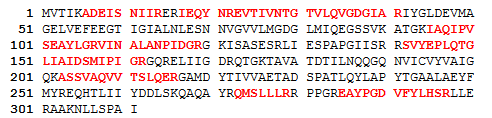


Matched peptides information:


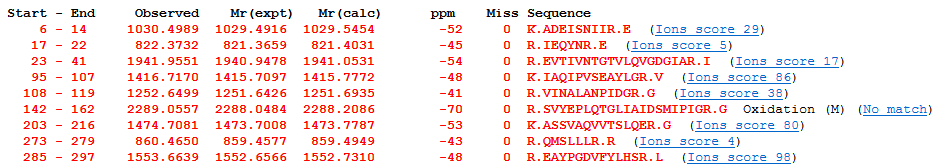


Spot No.:**363**

NCBI accession No.: **[GSBRNA2T00015387001](http://massboss/mascot/cgi/master_results.pl?file=../data/20150311/F012667.dat&REPTYPE=protein#Hit1)** Species: *Brassica napus*

PFF score: **188**

Protein name: **ATP synthase subunit alpha**

Matched peptides No.: **9**  Sequence coverage %: **32**

Calculated Mr: **33793**  Calculated *p*I: **5.09**

Probability Based Mow


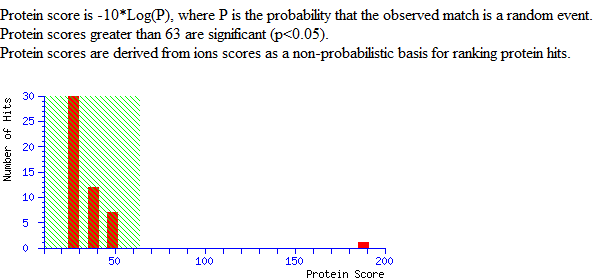


Matched peptide sequences: shown in Bold Red


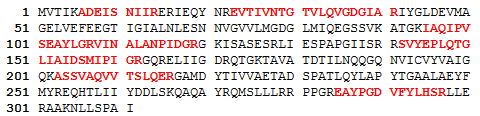


Matched peptides information:


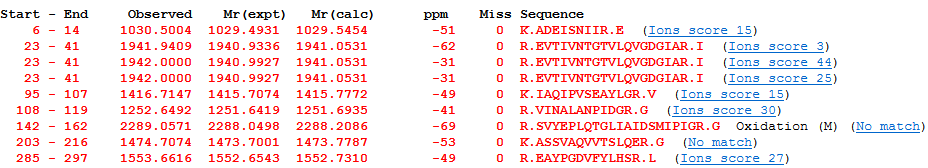


Spot No.: **376**

NCBI accession No.: **[GSBRNA2T00013010001](http://massboss/mascot/cgi/master_results.pl?file=../data/20150311/F012669.dat&REPTYPE=protein#Hit1)** Species: *Brassica napus*

PFF score: **95**

Protein name: **Trigger factor-like protein TIG**

Matched peptides No.: **3**  Sequence coverage %: **6**

Calculated Mr: **61574**  Calculated *p*I: **5.23**

Probability Based Mow


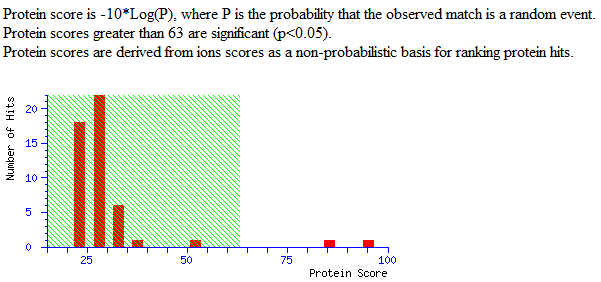


Matched peptide sequences: shown in Bold Red


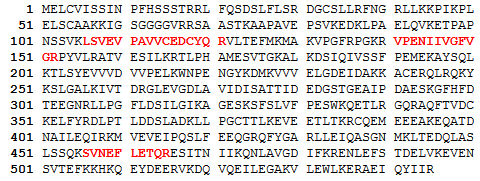


Matched peptides information:


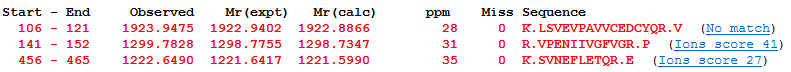


Spot No.: **378**

NCBI accession No.: **[GSBRNA2T00013010001](http://massboss/mascot/cgi/master_results.pl?file=../data/20150311/F012670.dat&REPTYPE=protein#Hit1)** Species: *Brassica napus*

PFF score: **158**

Protein name: **Trigger factor-like protein TIG**

Matched peptides No.: **4** Sequence coverage %: **6**

Calculated Mr: **61574**  Calculated *p*I: **5.23**

Probability Based Mow


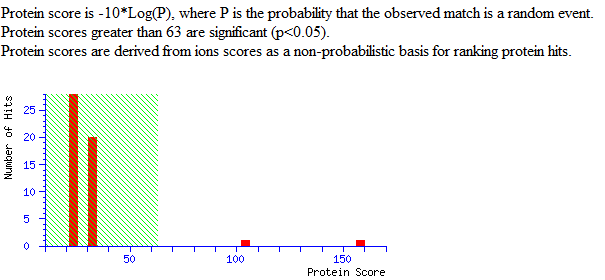


Matched peptide sequences: shown in Bold Red


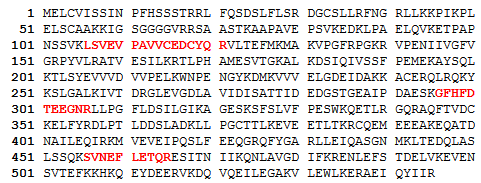


Matched peptides information:


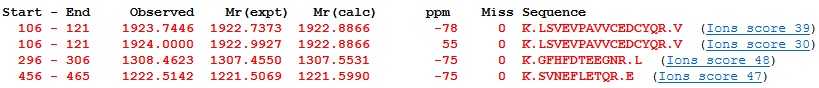


Spot No.: **383**

NCBI accession No.: **[GSBRNA2T00091196001](http://massboss/mascot/cgi/master_results.pl?file=../data/20150311/F012671.dat&REPTYPE=protein#Hit1)**  Species: *Brassica napus*

PFF score: **85**

Protein name: **Rhodanese-like domain-containing protein 4**

Matched peptides No.: **1** Sequence coverage %: **2**

Calculated Mr: **48532**  Calculated *p*I: **5.15**

Probability Based Mow


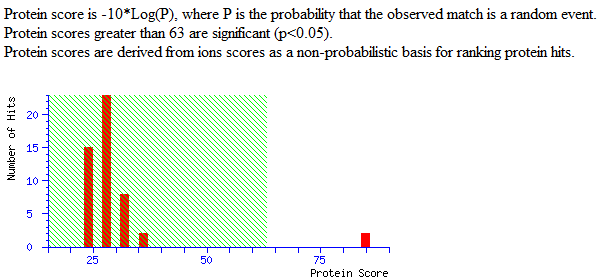


Matched peptide sequences: shown in Bold Red


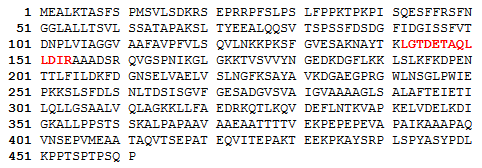


Matched peptides information:


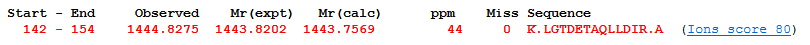


Spot No.: **390**

NCBI accession No.: **[GSBRNA2T0000321600](http://massboss/mascot/cgi/master_results.pl?file=../data/20150311/F012672.dat&REPTYPE=protein#Hit1)**1 Species: *Brassica napus*

PFF score: **558**

Protein name: **V-type proton ATPase subunit B3**

Matched peptides No.: **15** Sequence coverage %: **45**

Calculated Mr: **54141**  Calculated *p*I: **5.03**

Probability Based Mow


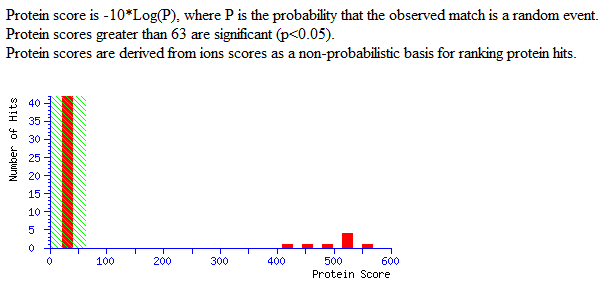


Matched peptide sequences: shown in Bold Red


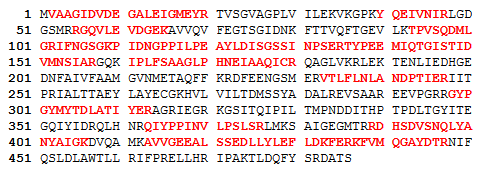


Matched peptides information:


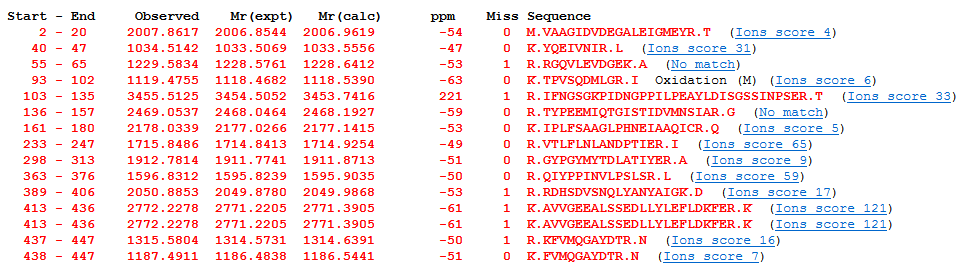


Spot No.: **414**

NCBI accession No.: **[GSBRNA2T00066465001](http://massboss/mascot/cgi/master_results.pl?file=../data/20150311/F012674.dat&REPTYPE=protein#Hit1)**  Species: *Brassica napus*

PFF score: **265**

Protein name: **ATP synthase subunit beta**

Matched peptides No.: **8**  Sequence coverage %: **20**

Calculated Mr: **65232**  Calculated *p*I: **5.17**

Probability Based Mow


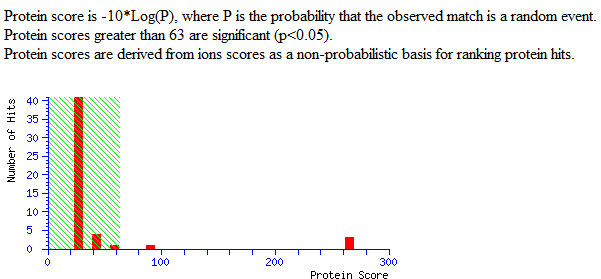


Matched peptide sequences: shown in Bold Red


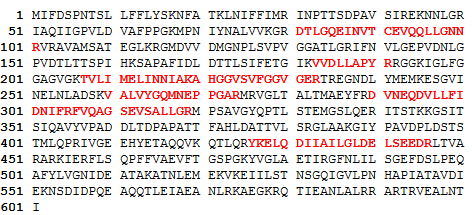


Matched peptides information:


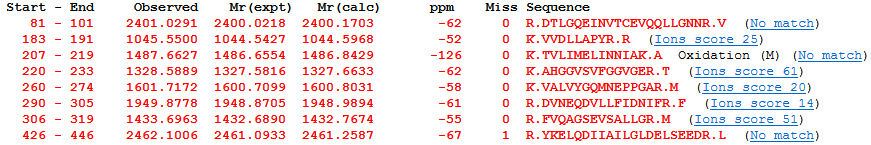


Spot No.: **422**

NCBI accession No.: **[GSBRNA2T00106253001](http://massboss/mascot/cgi/master_results.pl?file=../data/20150328/F016477.dat&REPTYPE=protein#Hit1)** Species: *Brassica napus*

PFF score: **129**

Protein name: **Ribulose bisphosphate carboxylase large chain**

Matched peptides No.: **2** Sequence coverage %: **16**

Calculated Mr: **14683**  Calculated *p*I: **5.01**

Probability Based Mow


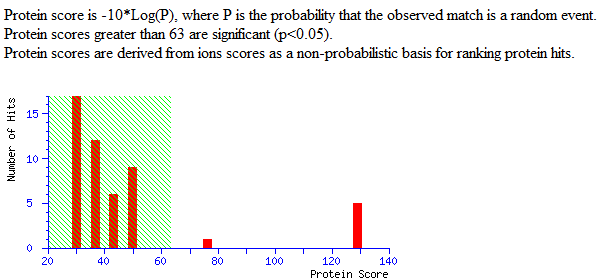


Matched peptide sequences: shown in Bold Red


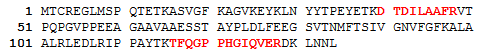


Matched peptides information:


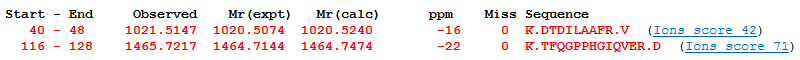


Spot No.: **435**

NCBI accession No.: **[GSBRNA2T00082162001](http://massboss/mascot/cgi/master_results.pl?file=../data/20150311/F012676.dat&REPTYPE=protein#Hit1)** Species: *Brassica napus*

PFF score: **353**

Protein name: **ATP synthase subunit alpha**

Matched peptides No.: **8** Sequence coverage %: **17**

Calculated Mr: **55308**  Calculated *p*I: **6.01**

Probability Based Mow


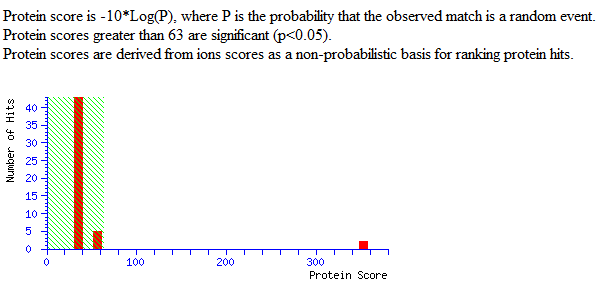


Matched peptide sequences: shown in Bold Red


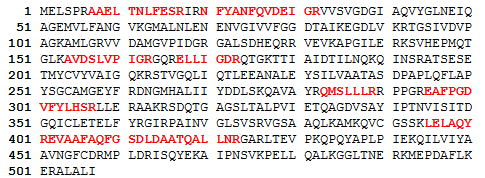


Matched peptides information:


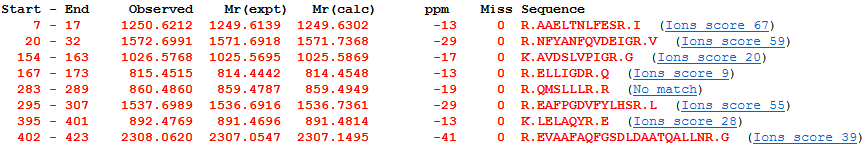


Spot No.: **444**

NCBI accession No.: **[GSBRNA2T00066465001](http://massboss/mascot/cgi/master_results.pl?file=../data/20150328/F016479.dat&REPTYPE=protein#Hit1)** Species: *Brassica napus*

PFF score: **475**

Protein name: **ATP synthase subunit beta**

Matched peptides No.: **8** Sequence coverage %: **16**

Calculated Mr: **65233**  Calculated *p*I: **5.17**

Probability Based Mow


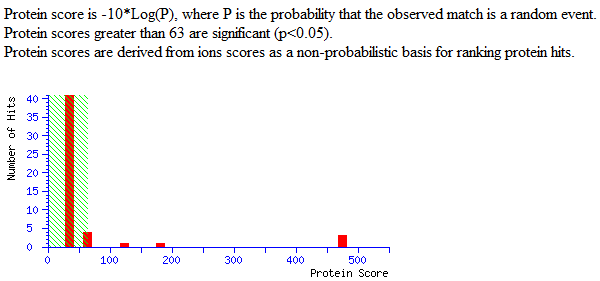


Matched peptide sequences: shown in Bold Red


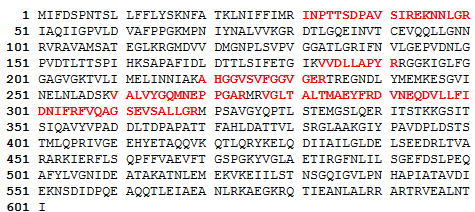


Matched peptides information:


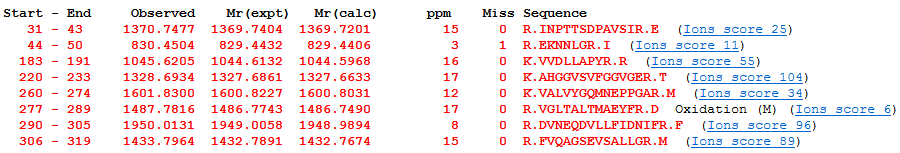


Spot No.: **447**

NCBI accession No.: **[GSBRNA2T00066465001](http://massboss/mascot/cgi/master_results.pl?file=../data/20150311/F012677.dat&REPTYPE=protein#Hit1)** Species: *Brassica napus*

PFF score: **265**

Protein name: **ATP synthase subunit beta**

Matched peptides No.: **8** Sequence coverage %: **20**

Calculated Mr: **65232**  Calculated *p*I: **5.17**

Probability Based Mow


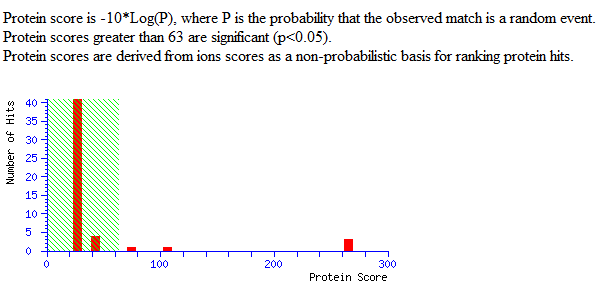


Matched peptide sequences: shown in Bold Red


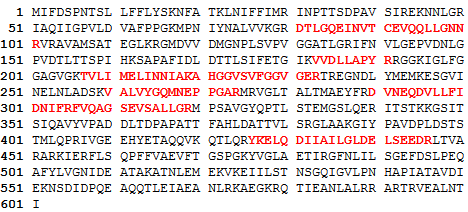


Matched peptides information:


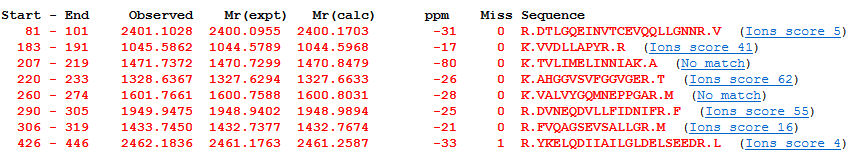


Spot No.: **453**

NCBI accession No.: **GSBRNA2T00066465001** Species: *Brassica napus*

PFF score: **815**

Protein name: **ATP synthase subunit beta**

Matched peptides No.: **11** Sequence coverage %: **29**

Calculated Mr: **65232**  Calculated *p*I: **5.17**

Probability Based Mow

Matched peptide sequences: shown in Bold Red

Matched peptides information:

Spot No.: **464**

NCBI accession No.: **[GSBRNA2T00015407001](http://massboss/mascot/cgi/master_results.pl?file=../data/20150311/F012680.dat&REPTYPE=protein#Hit1)** Species: *Brassica napus*

PFF score: **187**

Protein name: **Ribulose bisphosphate carboxylase large chain**

Matched peptides No.: **7** Sequence coverage %: **16**

Calculated Mr: **47775**  Calculated *p*I: **6.18**

Probability Based Mow

Matched peptide sequences: shown in Bold Red

Matched peptides information:

Spot No.: **474**

NCBI accession No.: **[GSBRNA2T00045232001](http://massboss/mascot/cgi/master_results.pl?file=../data/20150311/F012681.dat&REPTYPE=protein#Hit1)** Species: *Brassica napus*

PFF score: **211**

Protein name: **Chaperonin 60 subunit alpha 1**

Matched peptides No.: **6** Sequence coverage %: **13**

Calculated Mr: **61910**  Calculated *p*I: **5.15**

Probability Based Mow

Matched peptide sequences: shown in Bold Red

Matched peptides information:

Spot No.: **476**

NCBI accession No.: **[GSBRNA2T00059725001](http://massboss/mascot/cgi/master_results.pl?file=../data/20150311/F012682.dat&REPTYPE=protein#Hit1)**  Species: *Brassica napus*

PFF score: **86**

Protein name: **Probable UTP--glucose-1-phosphate uridylyltransferase 2**

Matched peptides No.: **4** Sequence coverage %: **34**

Calculated Mr: **16767**  Calculated *p*I: **5.52**

Probability Based Mow

Matched peptide sequences: shown in Bold Red

Matched peptides information:

Spot No.: **510**

NCBI accession No.: **[GSBRNA2T00005941001](http://massboss/mascot/cgi/master_results.pl?file=../data/20150311/F012683.dat&REPTYPE=protein#Hit1)** Species: *Brassica napus*

PFF score: **92**

Protein name: **26S protease regulatory subunit 6A homolog A**

Matched peptides No.: **5** Sequence coverage %: **16**

Calculated Mr: **47747**  Calculated *p*I: **4.92**

Probability Based Mow

Matched peptide sequences: shown in Bold Red

Matched peptides information:

Spot No.:**516**

NCBI accession No.: **[GSBRNA2T00025997001](http://massboss/mascot/cgi/master_results.pl?file=../data/20150324/F016338.dat&REPTYPE=protein#Hit1)**  Species: *Brassica napus*

PFF score: **105**

Protein name: **Translational initiation factor 4A-1 (EIF4A1)**

Matched peptides No.: **2** Sequence coverage %: **13**

Calculated Mr: **25024**  Calculated *p*I: **5.10**

Probability Based Mow

Matched peptide sequences: shown in Bold Red

Matched peptides information:

Spot No.:**519**

NCBI accession No.: **[GSBRNA2T00025997001](http://massboss/mascot/cgi/master_results.pl?file=../data/20150324/F016339.dat&REPTYPE=protein#Hit1)** Species: *Brassica napus*

PFF score: **150**

Protein name: **Translational initiation factor 4A-1**

Matched peptides No.: **3**  Sequence coverage %: **13**

Calculated Mr: **25024**  Calculated *p*I: **5.10**

Probability Based Mow

Matched peptide sequences: shown in Bold Red

Matched peptides information:

Spot No.: **527**

NCBI accession No.: **[GSBRNA2T00153196001](http://massboss/mascot/cgi/master_results.pl?file=../data/20150328/F016480.dat&REPTYPE=protein#Hit1)** Species: *Brassica napus*

PFF score: **163**

Protein name: **Glutamate--glyoxylate aminotransferase 1**

Matched peptides No.: **6**  Sequence coverage %: **17**

Calculated Mr: **44854**  Calculated *p*I: **5.83**

Probability Based Mow

Matched peptide sequences: shown in Bold Red

Matched peptides information:

Spot No.: **574**

NCBI accession No.: **[GSBRNA2T00137491001](http://massboss/mascot/cgi/master_results.pl?file=../data/20150311/F012688.dat&REPTYPE=protein#Hit1)** Species: *Brassica napus*

PFF score: **87**

Protein name: **S-adenosylmethionine synthase 4**

Matched peptides No.: **5** Sequence coverage %: **13**

Calculated Mr: **43260**  Calculated *p*I: **5.51**

Probability Based Mow

Matched peptide sequences: shown in Bold Red

Matched peptides information:

Spot No.: **585**

NCBI accession No.: **[GSBRNA2T00071053001](http://massboss/mascot/cgi/master_results.pl?file=../data/20150328/F016481.dat&REPTYPE=protein#Hit1)** Species: *Brassica napus*

PFF score: **184**

Protein name: **Fructose-1,6-bisphosphatase**

Matched peptides No.: **4**  Sequence coverage %: **10**

Calculated Mr: **44882**  Calculated *p*I: **5.40**

Probability Based Mow

Matched peptide sequences: shown in Bold Red

Matched peptides information:

Spot No.: **586**

NCBI accession No.: **[GSBRNA2T00028746001](http://massboss/mascot/cgi/master_results.pl?file=../data/20150328/F016482.dat&REPTYPE=protein#Hit1)** Species: *Brassica napus*

PFF score: **193**

Protein name: **Ribulose bisphosphate carboxylase/oxygenase activase**

Matched peptides No.: **5**  Sequence coverage %: **17**

Calculated Mr: **52187**  Calculated *p*I: **5.57**

Probability Based Mow

Matched peptide sequences: shown in Bold Red

Matched peptides information:

Spot No.:**587**

NCBI accession No.: **[GSBRNA2T00138076001](http://massboss/mascot/cgi/master_results.pl?file=../data/20150311/F012689.dat&REPTYPE=protein#Hit1)** Species: *Brassica napus*

PFF score: **522**

Protein name: **Elongation factor Tu, chloroplastic**

Matched peptides No.: **7**  Sequence coverage %: **29**

Calculated Mr: **30851** Calculated *p*I: **6.23**

Probability Based Mow

Matched peptide sequences: shown in Bold Red

Matched peptides information:

Spot No.: **590**

NCBI accession No.: **[GSBRNA2T00028746001](http://massboss/mascot/cgi/master_results.pl?file=../data/20150311/F012690.dat&REPTYPE=protein#Hit1)** Species: *Brassica napus*

PFF score: **507**

Protein name: **Ribulose bisphosphate carboxylase/oxygenase activase**

Matched peptides No.: **10** Sequence coverage %: **28**

Calculated Mr: **52181**  Calculated *p*I: **5.57**

Probability Based Mow

Matched peptide sequences: shown in Bold Red

Matched peptides information:

Spot No.: **596**

NCBI accession No.: **[GSBRNA2T00028746001](http://massboss/mascot/cgi/master_results.pl?file=../data/20150311/F012691.dat&REPTYPE=protein#Hit1)** Species: *Brassica napus*

PFF score: **243**

Protein name: **Ribulose bisphosphate carboxylase/oxygenase activase**

Matched peptides No.: **6** Sequence coverage %: **19**

Calculated Mr: **52181**  Calculated *p*I: **5.57**

Probability Based Mow

Matched peptide sequences: shown in Bold Red

Matched peptides information:

Spot No.: **597**

NCBI accession No.: **[GSBRNA2T00049292001](http://massboss/mascot/cgi/master_results.pl?file=../data/20150311/F012692.dat&REPTYPE=protein#Hit1)** Species: *Brassica napus*

PFF score: **158**

Protein name: **Probable monodehydroascorbate reductase**

Matched peptides No.: **5** Sequence coverage %: **11**

Calculated Mr: **47121**  Calculated *p*I: **5.09**

Probability Based Mow

Matched peptide sequences: shown in Bold Red

Matched peptides information:

Spot No.: **608**

NCBI accession No.: **[GSBRNA2T00012567001](http://massboss/mascot/cgi/master_results.pl?file=../data/20150328/F016483.dat&REPTYPE=protein#Hit1)** Species: *Brassica napus*

PFF score: **149**

Protein name: **Ribulose bisphosphate carboxylase/oxygenase activase**

Matched peptides No.: **4** Sequence coverage %: **14**

Calculated Mr: **51599**  Calculated *p*I: **6.29**

Probability Based Mow

Matched peptide sequences: shown in Bold Red

Matched peptides information:

Spot No.: **628**

NCBI accession No.: **[GSBRNA2T00091532001](http://massboss/mascot/cgi/master_results.pl?file=../data/20150311/F012694.dat&REPTYPE=protein#Hit1)** Species: *Brassica napus*

PFF score: **84**

Protein name: **GDP-mannose 3,5-epimerase**

Matched peptides No.: **4** Sequence coverage %: **10**

Calculated Mr: **57877**  Calculated *p*I: **8.95**

Probability Based Mow

Matched peptide sequences: shown in Bold Red

Matched peptides information:

Spot No.: **647**

NCBI accession No.: **[GSBRNA2T00107591001](http://massboss/mascot/cgi/master_results.pl?file=../data/20150328/F016486.dat&REPTYPE=protein#Hit1)** Species: *Brassica napus*

PFF score: **296**

Protein name: **Actin-11**

Matched peptides No.: **6** Sequence coverage %: **18**

Calculated Mr: **35789**  Calculated *p*I: **5.50**

Probability Based Mow

Matched peptide sequences: shown in Bold Red

Matched peptides information:

Spot No.: **674**

NCBI accession No.: **[GSBRNA2T00030156001](http://massboss/mascot/cgi/master_results.pl?file=../data/20150311/F012696.dat&REPTYPE=protein#Hit1)** Species: *Brassica napus*

PFF score: **110**

Protein name: **Cytosolic isocitrate dehydrogenase [NADP]**

Matched peptides No.: **5**  Sequence coverage %: **13**

Calculated Mr: **46349**  Calculated *p*I: **6.30**

Probability Based Mow

Matched peptide sequences: shown in Bold Red

Matched peptides information:

Spot No.: **686**

NCBI accession No.: **[GSBRNA2T00088488001](http://massboss/mascot/cgi/master_results.pl?file=../data/20150328/F016487.dat&REPTYPE=protein#Hit1)** Species: *Brassica napus*

PFF score: **169**

Protein name: **Glyceraldehyde-3-phosphate dehydrogenase GAPB**

Matched peptides No.: **5** Sequence coverage %: **11**

Calculated Mr: **43069**  Calculated *p*I: **5.59**

Probability Based Mow

Matched peptide sequences: shown in Bold Red

Matched peptides information:

Spot No.: **706**

NCBI accession No.: **[GSBRNA2T00064134001](http://massboss/mascot/cgi/master_results.pl?file=../data/20150328/F016488.dat&REPTYPE=protein#Hit1)** Species: *Brassica napus*

PFF score: **104**

Protein name: **Peptidyl-prolyl cis-trans isomerase CYP38**

Matched peptides No.: **2** Sequence coverage %: **8**

Calculated Mr: **47429**  Calculated *p*I: **5.07**

Probability Based Mow

Matched peptide sequences: shown in Bold Red

Matched peptides information:

Spot No.: **725**

NCBI accession No.: **[GSBRNA2T00028746001](http://massboss/mascot/cgi/master_results.pl?file=../data/20150311/F012699.dat&REPTYPE=protein#Hit1)** Species: *Brassica napus*

PFF score: **787**

Protein name: **Ribulose bisphosphate carboxylase/oxygenase activase**

Matched peptides No.: **9** Sequence coverage %: **23**

Calculated Mr: **52181**  Calculated *p*I: **5.57**

Probability Based Mow

Matched peptide sequences: shown in Bold Red

Matched peptides information:

Spot No.:**728**

NCBI accession No.: **[GSBRNA2T00028746001](http://massboss/mascot/cgi/master_results.pl?file=../data/20150328/F016490.dat&REPTYPE=protein#Hit1)** Species: *Brassica napus*

PFF score: **509**

Protein name: **Glutamine synthetase cytosolic isozyme 1-4**

Matched peptides No.: **9** Sequence coverage %: **22**

Calculated Mr: **52187**  Calculated *p*I: **5.57**

Probability Based Mow

Matched peptide sequences: shown in Bold Red

Matched peptides information:

Spot No.: **729**

NCBI accession No.: **[GSBRNA2T00097384001](http://massboss/mascot/cgi/master_results.pl?file=../data/20150311/F012701.dat&REPTYPE=protein#Hit1)** Species: *Brassica napus*

PFF score: **201**

Protein name: **Phosphoglycerate kinase**

Matched peptides No.: **3**  Sequence coverage %: **13**

Calculated Mr: **42219**  Calculated *p*I: **5.36**

Probability Based Mow

Matched peptide sequences: shown in Bold Red

Matched peptides information:

Spot No.: **734**

NCBI accession No.: **[GSBRNA2T00097384001](http://massboss/mascot/cgi/master_results.pl?file=../data/20150324/F016343.dat&REPTYPE=protein#Hit1)** Species: *Brassica napus*

PFF score: **172**

Protein name: **Phosphoglycerate kinase**

Matched peptides No.: **3**  Sequence coverage %: **10**

Calculated Mr: **42219**  Calculated *p*I: **5.36**

Probability Based Mow

Matched peptide sequences: shown in Bold Red

Matched peptides information:

Spot No.: **738**

NCBI accession No.: **[GSBRNA2T00037717001](http://massboss/mascot/cgi/master_results.pl?file=../data/20150311/F012704.dat&REPTYPE=protein#Hit1)** Species: *Brassica napus*

PFF score: **145**

Protein name: **Glutamate-1-semialdehyde 2,1-aminomutase 1**

Matched peptides No.: **4**  Sequence coverage %: **5**

Calculated Mr: **50600**  Calculated *p*I: **6.43**

Probability Based Mow

Matched peptide sequences: shown in Bold Red

Matched peptides information:

Spot No.: **739**

NCBI accession No.: **[GSBRNA2T00155196001](http://massboss/mascot/cgi/master_results.pl?file=../data/20150328/F016491.dat&REPTYPE=protein#Hit1)** Species: *Brassica napus*

PFF score: **329**

Protein name: **Ribulose bisphosphate carboxylase/oxygenase activase**

Matched peptides No.: **4** Sequence coverage %: **17**

Calculated Mr: **48300**  Calculated *p*I: **6.27**

Probability Based Mow

Matched peptide sequences: shown in Bold Red

Matched peptides information:

Spot No.: **741**

NCBI accession No.: **[GSBRNA2T00155196001](http://massboss/mascot/cgi/master_results.pl?file=../data/20150311/F012705.dat&REPTYPE=protein#Hit1)** Species: *Brassica napus*

PFF score: **565**

Protein name: **Ribulose bisphosphate carboxylase/oxygenase activase**

Matched peptides No.: **9** Sequence coverage %: **24**

Calculated Mr: **48296**  Calculated *p*I: **6.27**

Probability Based Mow

Matched peptide sequences: shown in Bold Red

Matched peptides information:

Spot No.: **790**

NCBI accession No.: **[GSBRNA2T00020049001](http://massboss/mascot/cgi/master_results.pl?file=../data/20150328/F016493.dat&REPTYPE=protein#Hit1)** Species: *Brassica napus*

PFF score: **108**

Protein name: **Probable cinnamyl alcohol dehydrogenase 9**

Matched peptides No.: **4**  Sequence coverage %: **17**

Calculated Mr: **39302**  Calculated *p*I: **6.16**

Probability Based Mow

Matched peptide sequences: shown in Bold Red

Matched peptides information:

Spot No.: **813**

NCBI accession No.: **[GSBRNA2T00155196001](http://massboss/mascot/cgi/master_results.pl?file=../data/20150311/F012708.dat&REPTYPE=protein#Hit1)** Species: *Brassica napus*

PFF score: **328**

Protein name: **Ribulose bisphosphate carboxylase/oxygenase activase**

Matched peptides No.: **8** Sequence coverage %: **18**

Calculated Mr: **48296**  Calculated *p*I: **6.27**

Probability Based Mow

Matched peptide sequences: shown in Bold Red

Matched peptides information:

Spot No.: **842**

NCBI accession No.: **[GSBRNA2T00092896001](http://massboss/mascot/cgi/master_results.pl?file=../data/20150328/F016494.dat&REPTYPE=protein#Hit1)** Species: *Brassica napus*

PFF score: **187**

Protein name: **Glyceraldehyde-3-phosphate dehydrogenase GAPC2**

Matched peptides No.: **4** Sequence coverage %: **13**

Calculated Mr: **36747**  Calculated *p*I: **6.44**

Probability Based Mow

Matched peptide sequences: shown in Bold Red

Matched peptides information:

Spot No.: **887**

NCBI accession No.: **GSBRNA2T00053446001** Species: *Brassica napus*

PFF score: **132**

Protein name: **Plasma membrane-associated cation-binding protein 1**

Matched peptides No.: **2** Sequence coverage %: **15**

Calculated Mr: **26900**  Calculated *p*I: **4.93**

Probability Based Mow

Matched peptide sequences: shown in Bold Red

Matched peptides information:

Spot No.: **901**

NCBI accession No.: **[GSBRNA2T00136999001](http://massboss/mascot/cgi/master_results.pl?file=../data/20150311/F012712.dat&REPTYPE=protein#Hit1)** Species: *Brassica napus*

PFF score: **108**

Protein name: **Probable fructose-bisphosphate aldolase 1**

Matched peptides No.: **3**  Sequence coverage %: **12**

Calculated Mr: **42955**  Calculated *p*I: **6.78**

Probability Based Mow

Matched peptide sequences: shown in Bold Red

Matched peptides information:

Spot No.: **912**

NCBI accession No.: **[GSBRNA2T00015652001](http://massboss/mascot/cgi/master_results.pl?file=../data/20150328/F016498.dat&REPTYPE=protein#Hit1)** Species: *Brassica napus*

PFF score: **60**

Protein name: **ATP synthase gamma chain 1**

Matched peptides No.: **2**  Sequence coverage %: **6**

Calculated Mr: **41304**  Calculated *p*I: **6.78**

Probability Based Mow

Matched peptide sequences: shown in Bold Red

Matched peptides information:

Spot No.: **913**

NCBI accession No.: **[GSBRNA2T00136999001](http://massboss/mascot/cgi/master_results.pl?file=../data/20150328/F016499.dat&REPTYPE=protein#Hit1)** Species: *Brassica napus*

PFF score: **104**

Protein name: **Probable fructose-bisphosphate aldolase 1**

Matched peptides No.: **5** Sequence coverage %: **20**

Calculated Mr: **42958**  Calculated *p*I: **6.78**

Probability Based Mow

Matched peptide sequences: shown in Bold Red

Matched peptides information:

Spot No.: **933**

NCBI accession No.: **[GSBRNA2T00092222001](http://massboss/mascot/cgi/master_results.pl?file=../data/20150328/F016500.dat&REPTYPE=protein#Hit1)** Species: *Brassica napus*

PFF score: **89**

Protein name: **Epithiospecifier protein**

Matched peptides No.: **5** Sequence coverage %: **15**

Calculated Mr: **37880**  Calculated *p*I: **5.95**

Probability Based Mow

Matched peptide sequences: shown in Bold Red

Matched peptides information:

Spot No.: **938**

NCBI accession No.: **[GSBRNA2T00136999001](http://massboss/mascot/cgi/master_results.pl?file=../data/20150311/F012717.dat&REPTYPE=protein#Hit1)** Species: *Brassica napus*

PFF score: **298**

Protein name: **Probable fructose-bisphosphate aldolase 1**

Matched peptides No.: **6** Sequence coverage %: **26**

Calculated Mr: **42955**  Calculated *p*I: **6.78**

Probability Based Mow

Matched peptide sequences: shown in Bold Red

Matched peptides information:

Spot No.: **947**

NCBI accession No.: **[GSBRNA2T00064132001](http://massboss/mascot/cgi/master_results.pl?file=../data/20150311/F012718.dat&REPTYPE=protein#Hit1)** Species: *Brassica napus*

PFF score: **67**

Protein name: **Beta carbonic anhydrase 1**

Matched peptides No.: **2** Sequence coverage %: **9**

Calculated Mr: **38178**  Calculated *p*I: **6.26**

Probability Based Mow

Matched peptide sequences: shown in Bold Red

Matched peptides information:

Spot No.: **976**

NCBI accession No.: **[GSBRNA2T00022009001](http://massboss/mascot/cgi/master_results.pl?file=../data/20150311/F012719.dat&REPTYPE=protein#Hit1)** Species: *Brassica napus*

PFF score: **351**

Protein name: **Cysteine synthase**

Matched peptides No.: **5** Sequence coverage %: **19**

Calculated Mr: **41520**  Calculated *p*I: **7.55**

Probability Based Mow

Matched peptide sequences: shown in Bold Red

Matched peptides information:

Spot No.: **1003**

NCBI accession No.: **[GSBRNA2T00015621001](http://massboss/mascot/cgi/master_results.pl?file=../data/20150311/F012720.dat&REPTYPE=protein#Hit1)** Species: *Brassica napus*

PFF score: **173**

Protein name: **Probable plastid-lipid-associated protein 1**

Matched peptides No.: **3** Sequence coverage %: **14**

Calculated Mr: **34666**  Calculated *p*I: **6.86**

Probability Based Mow

Matched peptide sequences: shown in Bold Red

Matched peptides information:

Spot No.: **1006**

NCBI accession No.: **[GSBRNA2T00015621001](http://massboss/mascot/cgi/master_results.pl?file=../data/20150328/F016502.dat&REPTYPE=protein#Hit1)** Species: *Brassica napus*

PFF score: **110**

Protein name: **Probable plastid-lipid-associated protein 1**

Matched peptides No.: **4** Sequence coverage %: **14**

Calculated Mr: **34667**  Calculated *p*I: **6.86**

Probability Based Mow

Matched peptide sequences: shown in Bold Red

Matched peptides information:

Spot No.: **1045**

NCBI accession No.: **[GSBRNA2T00094536001](http://massboss/mascot/cgi/master_results.pl?file=../data/20150311/F012723.dat&REPTYPE=protein#Hit1)** Species: *Brassica napus*

PFF score: **135**

Protein name: **Probable plastid-lipid-associated protein 1**

Matched peptides No.: **5** Sequence coverage %: **19**

Calculated Mr: **34880**  Calculated *p*I: **5.37**

Probability Based Mow

Matched peptide sequences: shown in Bold Red

Matched peptides information:

Spot No.: **1069**

NCBI accession No.: **[GSBRNA2T00076633001](http://massboss/mascot/cgi/master_results.pl?file=../data/20150311/F012724.dat&REPTYPE=protein#Hit1)** Species: *Brassica napus*

PFF score: **162**

Protein name: **Ferredoxin--NADP reductase, leaf isozyme 2**

Matched peptides No.: **3**  Sequence coverage %: **11**

Calculated Mr: **40847**  Calculated *p*I: **8.79**

Probability Based Mow

Matched peptide sequences: shown in Bold Red

Matched peptides information:

Spot No.: **1085**

NCBI accession No.: **[GSBRNA2T00146509001](http://massboss/mascot/cgi/master_results.pl?file=../data/20150311/F012726.dat&REPTYPE=protein#Hit1)** Species: *Brassica napus*

PFF score: **109**

Protein name: **L-ascorbate peroxidase T**

Matched peptides No.: **2** Sequence coverage %: **6**

Calculated Mr: **47611**  Calculated *p*I: **7.70**

Probability Based Mow

Matched peptide sequences: shown in Bold Red

Matched peptides information:

Spot No.: **1086**

NCBI accession No.: **[GSBRNA2T00028746001](http://massboss/mascot/cgi/master_results.pl?file=../data/20150311/F012727.dat&REPTYPE=protein#Hit1)** Species: *Brassica napus*

PFF score: **105**

Protein name: **Ribulose bisphosphate carboxylase/oxygenase activase**

Matched peptides No.: **8** Sequence coverage %: **23**

Calculated Mr: **52181** Calculated pI: **5.57**

Probability Based Mow

Matched peptide sequences: shown in Bold Red

Matched peptides information:

Spot No.: **1117**

NCBI accession No.: **[GSBRNA2T00005668001](http://massboss/mascot/cgi/master_results.pl?file=../data/20150328/F016462.dat&REPTYPE=protein#Hit1)** Species: *Brassica napus*

PFF score: **73**

Protein name: **Oxygen-evolving enhancer protein 1-1**

Matched peptides No.: **3** Sequence coverage %: **10**

Calculated Mr: **35357**  Calculated *p*I: **5.55**

Probability Based Mow

Matched peptide sequences: shown in Bold Red

Matched peptides information:

Spot No.: **1125**

NCBI accession No.: **[GSBRNA2T00005668001](http://massboss/mascot/cgi/master_results.pl?file=../data/20150329/F016511.dat&REPTYPE=protein#Hit1)** Species: *Brassica napus*

PFF score: **342**

Protein name: **Oxygen-evolving enhancer protein 1-1**

Matched peptides No.: **6** Sequence coverage %: **18**

Calculated Mr: **35357**  Calculated *p*I: **5.55**

Probability Based Mow

Matched peptide sequences: shown in Bold Red

Matched peptides information:

Spot No.: **1129**

NCBI accession No.: **[GSBRNA2T00112035001](http://massboss/mascot/cgi/master_results.pl?file=../data/20150311/F012730.dat&REPTYPE=protein#Hit1)** Species: *Brassica napus*

PFF score: **276**

Protein name: **14-3-3-like protein GF14 nu**

Matched peptides No.: **5** Sequence coverage %: **22**

Calculated Mr: **29535**  Calculated *p*I: **4.69**

Probability Based Mow

Matched peptide sequences: shown in Bold Red

Matched peptides information:

Spot No.: **1138**

NCBI accession No.: **[GSBRNA2T00015407001](http://massboss/mascot/cgi/master_results.pl?file=../data/20150324/F016351.dat&REPTYPE=protein#Hit1)** Species: *Brassica napus*

PFF score: **86**

Protein name: **Ribulose bisphosphate carboxylase large chain**

Matched peptides No.: **4** Sequence coverage %: **12**

Calculated Mr: **47775**  Calculated *p*I: **6.18**

Probability Based Mow

Matched peptide sequences: shown in Bold Red

Matched peptides information:

Spot No.: **1160**

NCBI accession No.: **[GSBRNA2T00134966001](http://massboss/mascot/cgi/master_results.pl?file=../data/20150311/F012733.dat&REPTYPE=protein#Hit1)** Species: *Brassica napus*

PFF score: **537**

Protein name: **Oxygen-evolving enhancer protein 1-1**

Matched peptides No.: **6** Sequence coverage %: **26**

Calculated Mr: **35394**  Calculated *p*I: **5.77**

Probability Based Mow

Matched peptide sequences: shown in Bold Red

Matched peptides information:

Spot No.: **1165**

NCBI accession No.: **[GSBRNA2T00013874001](http://massboss/mascot/cgi/master_results.pl?file=../data/20150311/F012734.dat&REPTYPE=protein#Hit1)** Species: *Brassica napus*

PFF score: **138**

Protein name: **14-3-3-like protein GF14 psi**

Matched peptides No.: **2** Sequence coverage %: **11**

Calculated Mr: **28987** Calculated *p*I: **4.76**

Probability Based Mow

Matched peptide sequences: shown in Bold Red

Matched peptides information:

Spot No.:**1183**

NCBI accession No.: **[GSBRNA2T00066463001](http://massboss/mascot/cgi/master_results.pl?file=../data/20150311/F012736.dat&REPTYPE=protein#Hit1)** Species: *Brassica napus*

PFF score: **74**

Protein name: **Ribulose bisphosphate carboxylase large chain**

Matched peptides No.: **3** Sequence coverage %: **7**

Calculated Mr: **29992**  Calculated *p*I: **6.00**

Probability Based Mow

Matched peptide sequences: shown in Bold Red

Matched peptides information:

Spot No.: **1203**

NCBI accession No.: **[GSBRNA2T00103728001](http://massboss/mascot/cgi/master_results.pl?file=../data/20150311/F012738.dat&REPTYPE=protein#Hit1)** Species: *Brassica napus*

PFF score: **200**

Protein name: **Thioredoxin-like protein CDSP32**

Matched peptides No.: **5** Sequence coverage %: **24**

Calculated Mr: **34351**  Calculated *p*I: **8.40**

Probability Based Mow

Matched peptide sequences: shown in Bold Red

Matched peptides information:

Spot No.: **1212**

NCBI accession No.: **[GSBRNA2T00125328001](http://massboss/mascot/cgi/master_results.pl?file=../data/20150311/F012739.dat&REPTYPE=protein#Hit1)** Species: *Brassica napus*

PFF score: **159**

Protein name: **Haloacid dehalogenase-like hydrolase domain-containing protein**

Matched peptides No.: **4** Sequence coverage %: **18**

Calculated Mr: **34775**  Calculated *p*I: **8.49**

Probability Based Mow

Matched peptide sequences: shown in Bold Red

Matched peptides information:

Spot No.: **1214**

NCBI accession No.: **GSBRNA2T00068136001** Species: *Brassica napus*

PFF score: **62**

Protein name: **Haloacid dehalogenase-like hydrolase domain-containing protein**

Matched peptides No.: **4** Sequence coverage %: **10**

Calculated Mr: **34899**  Calculated *p*I: **8.3**

Probability Based Mow

Matched peptide sequences: shown in Bold Red

Matched peptides information:

Spot No.: **1234**

NCBI accession No.: **GSBRNA2T00032385001** Species: *Brassica napus*

PFF score: **85**

Protein name: **Uncharacterized protein At5g02240**

Matched peptides No.: **2** Sequence coverage %: **8**

Calculated Mr: **27221**  Calculated *p*I: **6.19**

Probability Based Mow

Matched peptide sequences: shown in Bold Red

Matched peptides information:

Spot No.: **1255**

NCBI accession No.: **[GSBRNA2T00096646001](http://massboss/mascot/cgi/master_results.pl?file=../data/20150311/F012741.dat&REPTYPE=protein#Hit1)** Species: *Brassica napus*

PFF score: **278**

Protein name: **Proteasome** **subunit alpha type-6-B**

Matched peptides No.: **7** Sequence coverage %: **24**

Calculated Mr: **27269** Calculated *p*I: **5.48**

Probability Based Mow

Matched peptide sequences: shown in Bold Red

Matched peptides information:

Spot No.: **1267**

NCBI accession No.: **[GSBRNA2T00119786001](http://massboss/mascot/cgi/master_results.pl?file=../data/20150311/F012744.dat&REPTYPE=protein#Hit1)**  Species: *Brassica napus*

PFF score: **149**

Protein name: **Thylakoid lumenal 29 kDa protein**

Matched peptides No.: **4** Sequence coverage %: **8**

Calculated Mr: **38124** Calculated *p*I: **8.59**

Probability Based Mow

Matched peptide sequences: shown in Bold Red

Matched peptides information:

Spot No.: **1271**

NCBI accession No.: **[GSBRNA2T00100654001](http://massboss/mascot/cgi/master_results.pl?file=../data/20150324/F016323.dat&REPTYPE=protein#Hit1)** Species: *Brassica napus*

PFF score: **397**

Protein name: **L-ascorbate peroxidase 1**

Matched peptides No.: **6** Sequence coverage %: **34**

Calculated Mr: **27879**  Calculated *p*I: **5.58**

Probability Based Mow

Matched peptide sequences: shown in Bold Red

Matched peptides information:

Spot No.: **1272**

NCBI accession No.: **[GSBRNA2T00122207001](http://massboss/mascot/cgi/master_results.pl?file=../data/20150311/F012746.dat&REPTYPE=protein#Hit1)** Species: *Brassica napus*

PFF score: **432**

Protein name: **L-ascorbate peroxidase 1**

Matched peptides No.: **8**  Sequence coverage %: **36**

Calculated Mr: **28258**  Calculated *p*I: **5.86**

Probability Based Mow

Matched peptide sequences: shown in Bold Red

Matched peptides information:

Spot No.: **1279**

NCBI accession No.: **[GSBRNA2T00090276001](http://massboss/mascot/cgi/master_results.pl?file=../data/20150311/F012747.dat&REPTYPE=protein#Hit1)** Species: *Brassica napus*

PFF score: **281**

Protein name: **Triosephosphate isomerase**

Matched peptides No.: **4** Sequence coverage %: **21**

Calculated Mr: **33451**  Calculated *p*I: **6.90**

Probability Based Mow

Matched peptide sequences: shown in Bold Red

Matched peptides information:

Spot No.: **1285**

NCBI accession No.: **[GSBRNA2T00013748001](http://massboss/mascot/cgi/master_results.pl?file=../data/20150311/F012748.dat&REPTYPE=protein#Hit1)**  Species: *Brassica napus*

PFF score: **79**

Protein name: **Putative uncharacterized protein T16L4.100**

Matched peptides No.: **1** Sequence coverage %: **5**

Calculated Mr: **34614**  Calculated *p*I: **6.13**

Probability Based Mow

Matched peptide sequences: shown in Bold Red

Matched peptides information:

Spot No.: **1286**

NCBI accession No.: **[GSBRNA2T00100654001](http://massboss/mascot/cgi/master_results.pl?file=../data/20150311/F012749.dat&REPTYPE=protein#Hit1)** Species: *Brassica napus*

PFF score: **464**

Protein name: **L-ascorbate peroxidase 1**

Matched peptides No.: **9** Sequence coverage %: **50**

Calculated Mr: **27879**  Calculated *p*I: **5.58**

Probability Based Mow

Matched peptide sequences: shown in Bold Red

Matched peptides information:

Spot No.: **1294**

NCBI accession No.: **[GSBRNA2T00035007001](http://massboss/mascot/cgi/master_results.pl?file=../data/20150311/F012750.dat&REPTYPE=protein#Hit1)** Species: *Brassica napus*

PFF score: **111**

Protein name: **Chlorophyll a-b binding protein 1**

Matched peptides No.: **1** Sequence coverage %: **4**

Calculated Mr: **28422**  Calculated *p*I: **5.33**

Probability Based Mow

Matched peptide sequences: shown in Bold Red

Matched peptides information:

Spot No.: **1304**

NCBI accession No.: **[GSBRNA2T00018693001](http://massboss/mascot/cgi/master_results.pl?file=../data/20150324/F016353.dat&REPTYPE=protein#Hit1)** Species: *Brassica napus*

PFF score: **95**

Protein name: **Proteasome subunit alpha type-2-A**

Matched peptides No.: **4**  Sequence coverage %: **8**

Calculated Mr: **25685**  Calculated *p*I: **5.53**

Probability Based Mow

Matched peptide sequences: shown in Bold Red

Matched peptides information:

Spot No.: **1309**

NCBI accession No.: **[GSBRNA2T00119583001](http://massboss/mascot/cgi/master_results.pl?file=../data/20150311/F012753.dat&REPTYPE=protein#Hit1)** Species: *Brassica napus*

PFF score: **149**

Protein name: **Glutathione S-transferase L3**

Matched peptides No.: **5**  Sequence coverage %: **25**

Calculated Mr: **31999**  Calculated *p*I: **5.13**

Probability Based Mow

Matched peptide sequences: shown in Bold Red

Matched peptides information:

Spot No.: **1319**

NCBI accession No.: **[GSBRNA2T00064132001](http://massboss/mascot/cgi/master_results.pl?file=../data/20150311/F012754.dat&REPTYPE=protein#Hit1)** Species: *Brassica napus*

PFF score: **317**

Protein name: **Beta carbonic anhydrase 1**

Matched peptides No.: **4** Sequence coverage %: **17**

Calculated Mr: **38178**  Calculated *p*I: **6.26**

Probability Based Mow

Matched peptide sequences: shown in Bold Red

Matched peptides information:

Spot No.: **1323**

NCBI accession No.: **[GSBRNA2T00115210001](http://massboss/mascot/cgi/master_results.pl?file=../data/20150311/F012756.dat&REPTYPE=protein#Hit1)** Species: *Brassica napus*

PFF score: **108**

Protein name: **Triosephosphate isomerase**

Matched peptides No.: **3**  Sequence coverage %: **5**

Calculated Mr: **27229**  Calculated *p*I: **5.73**

Probability Based Mow

Matched peptide sequences: shown in Bold Red

Matched peptides information:

Spot No.: **1327**

NCBI accession No.: **gi|297787439** Species: *Brassica napus*

PFF score: **159**

Protein name: **Beta carbonic anhydrase 1**

Matched peptides No.: **2** Sequence coverage %: **7**

Calculated Mr: **36127**  Calculated *p*I: **5.47**

Probability Based Mow

Matched peptide sequences: shown in Bold Red

Matched peptides information:

Spot No.: **1328**

NCBI accession No.: **gi|297787439** Species: *Brassica napus*

PFF score: **586**

Protein name: **Beta carbonic anhydrase 1**

Matched peptides No.: **7** Sequence coverage %: **26**

Calculated Mr: **36127** Calculated *p*I: **5.47**

Probability Based Mow

Matched peptide sequences: shown in Bold Red

Matched peptides information:

Spot No.: **1339**

NCBI accession No.: **gi|297787439** Species: *Brassica napus*

PFF score: **6135**

Protein name: **Beta carbonic anhydrase 1, chloroplastic**

Matched peptides No.: **15** Sequence coverage %: **63**

Calculated Mr: **36127** Calculated *p*I: **5.47**

Probability Based Mow

Matched peptide sequences: shown in Bold Red

Matched peptides information:

Spot No.: **1340**

NCBI accession No.: [**GSBRNA2T00041298001**](http://massboss/mascot/cgi/protein_view.pl?file=../data/20150311/F012758.dat&hit=1) Species: *Brassica napus*

PFF score: **188**

Protein name: **Beta carbonic anhydrase 1**

Matched peptides No.: **4**  Sequence coverage %: **17**

Calculated Mr: **37546**  Calculated *p*I:**6.47**

Probability Based Mow

Matched peptide sequences: shown in Bold Red

Matched peptides information:

Spot No.: **1343**

NCBI accession No.: **gi|297787439** Species: *Brassica napus*

PFF score: **275**

Protein name: **Beta carbonic anhydrase 1**

Matched peptides No.: **3** Sequence coverage %: **15**

Calculated Mr: **36127**  Calculated *p*I: **5.47**

Probability Based Mow

Matched peptide sequences: shown in Bold Red

Matched peptides information:

Spot No.: **1351**

NCBI accession No.: **[GSBRNA2T00043017001](http://massboss/mascot/cgi/master_results.pl?file=../data/20150311/F012760.dat&REPTYPE=protein#Hit1)** Species: *Brassica napus*

PFF score: **200**

Protein name: **Glutathione S-transferase DHAR1**

Matched peptides No.: **2** Sequence coverage %: **12**

Calculated Mr: **23647**  Calculated *p*I: **5.76**

Probability Based Mow

Matched peptide sequences: shown in Bold Red

Matched peptides information:

Spot No.: **1352**

NCBI accession No.: **[GSBRNA2T00084355001](http://massboss/mascot/cgi/master_results.pl?file=../data/20150311/F012761.dat&REPTYPE=protein#Hit1)** Species: *Brassica napus*

PFF score: **100**

Protein name: **Glutathione S-transferase U19**

Matched peptides No.: **2** Sequence coverage %: **7**

Calculated Mr: **25909**  Calculated *p*I: **5.23**

Probability Based Mow

Matched peptide sequences: shown in Bold Red

Matched peptides information:

Spot No.: **1355**

NCBI accession No.: **[GSBRNA2T00049208001](http://massboss/mascot/cgi/master_results.pl?file=../data/20150329/F016518.dat&REPTYPE=protein#Hit1)** Species: *Brassica napus*

PFF score: **171**

Protein name: **Ferritin-1**

Matched peptides No.: **4** Sequence coverage %: **20**

Calculated Mr: **28223**  Calculated *p*I: **5.61**

Probability Based Mow

Matched peptide sequences: shown in Bold Red

Matched peptides information:

Spot No.:**1357**

NCBI accession No.: **[GSBRNA2T00155196001](http://massboss/mascot/cgi/master_results.pl?file=../data/20150329/F016519.dat&REPTYPE=protein#Hit1)** Species: *Brassica napus*

PFF score: **100**

Protein name: **Ribulose bisphosphate carboxylase/oxygenase activase**

Matched peptides No.: **4** Sequence coverage %: **17**

Calculated Mr: **48300**  Calculated *p*I: **6.27**

Probability Based Mow

Matched peptide sequences: shown in Bold Red

Matched peptides information:

Spot No.: **1360**

NCBI accession No.: **gi|297787439** Species: *Brassica napus*

PFF score: **630**

Protein name: **Beta carbonic anhydrase 1**

Matched peptides No.: **14** Sequence coverage %: **55**

Calculated Mr: **36127**  Calculated *p*I: **5.47**

Probability Based Mow

Matched peptide sequences: shown in Bold Red

Matched peptides information:

Spot No.: **1361**

NCBI accession No.: **gi|297787439** Species: *Brassica napus*

PFF score: **489**

Protein name: **Beta carbonic anhydrase 1**

Matched peptides No.: **5** Sequence coverage %:**23**

Calculated Mr: **36127**  Calculated *p*I: **5.47**

Probability Based Mow

Matched peptide sequences: shown in Bold Red

Matched peptides information:

Spot No.: **1370**

NCBI accession No.: **gi|297787439** Species: *Brassica napus*

PFF score: **218**

Protein name: **Beta carbonic anhydrase 1**

Matched peptides No.: **7** Sequence coverage %: **15**

Calculated Mr: **36127**  Calculated *p*I: **5.47**

Probability Based Mow

Matched peptide sequences: shown in Bold Red

Matched peptides information:

Spot No.: **1372**

NCBI accession No.: **[GSBRNA2T00025200001](http://massboss/mascot/cgi/master_results.pl?file=../data/20150311/F012766.dat&REPTYPE=protein#Hit1)** Species: *Brassica napus*

PFF score: **149**

Protein name: **Glutathione S-transferase U5**

Matched peptides No.: **5** Sequence coverage %: **17**

Calculated Mr: **25802**  Calculated *p*I: **6.04**

Probability Based Mow

Matched peptide sequences: shown in Bold Red

Matched peptides information:

Spot No.: **1379**

NCBI accession No.: **[GSBRNA2T00099235001](http://massboss/mascot/cgi/master_results.pl?file=../data/20150311/F012768.dat&REPTYPE=protein#Hit1)** Species: *Brassica napus*

PFF score: **105**

Protein name: **Glutathione S-transferase U19**

Matched peptides No.: **3** Sequence coverage %: **15**

Calculated Mr: **25784**   Calculated *p*I:**6.37**

Probability Based Mow

Matched peptide sequences: shown in Bold Red

Matched peptides information:

Spot No.: **1415**

NCBI accession No.: **[GSBRNA2T00043389001](http://massboss/mascot/cgi/master_results.pl?file=../data/20150324/F016359.dat&REPTYPE=protein#Hit1)** Species: *Brassica napus*

PFF score: **113**

Protein name: **Glutathione S-transferase F9**

Matched peptides No.: **3**  Sequence coverage %: **10**

Calculated Mr: **24229**  Calculated *p*I: **5.98**

Probability Based Mow

Matched peptide sequences: shown in Bold Red

Matched peptides information:

Spot No.: **1423**

NCBI accession No.: **GSBRNA2T00101634001** Species: *Brassica napus*

PFF score: **67**

Protein name: **Glutathione S-transferase F8**

Matched peptides No.: **2** Sequence coverage %: **7**

Calculated Mr: **24427**  Calculated *p*I: **5.99**

Probability Based Mow

Matched peptide sequences: shown in Bold Red

Matched peptides information:

Spot No.:**1426**

NCBI accession No.: **[GSBRNA2T00044206001](http://massboss/mascot/cgi/master_results.pl?file=../data/20150324/F016360.dat&REPTYPE=protein#Hit1)** Species: *Brassica napus*

PFF score: **116**

Protein name: **Similar to SOUL Protein**

Matched peptides No.: **2**  Sequence coverage %: **19**

Calculated Mr: **25703** Calculated *p*I: **4.73**

Probability Based Mow

Matched peptide sequences: shown in Bold Red

Matched peptides information:

Spot No.: **1430**

NCBI accession No.: **[GSBRNA2T00066856001](http://massboss/mascot/cgi/master_results.pl?file=../data/20150311/F012775.dat&REPTYPE=protein#Hit1)** Species: *Brassica napus*

PFF score: **66**

Protein name: **2-Cys peroxiredoxin BAS1-like**

Matched peptides No.: **2** Sequence coverage %: **15**

Calculated Mr: **27507** Calculated *p*I: **5.00**

Probability Based Mow

Matched peptide sequences: shown in Bold Red

Matched peptides information:

Spot No.: **1478**

NCBI accession No.: **[GSBRNA2T00002240001](http://massboss/mascot/cgi/master_results.pl?file=../data/20150311/F012778.dat&REPTYPE=protein#Hit1)** Species: *Brassica napus*

PFF score: **175**

Protein name: **Uncharacterized protein**

Matched peptides No.: **6** Sequence coverage %: **21**

Calculated Mr: **31657** Calculated *p*I: **6.00**

Probability Based Mow

Matched peptide sequences: shown in Bold Red

Matched peptides information:

Spot No.: **1492**

NCBI accession No.: **GSBRNA2T00004321001** Species: *Brassica napus*

PFF score: **60**

Protein name: **Germin-like protein subfamily 3 member 3**

Matched peptides No.: **1** Sequence coverage %: **10**

Calculated Mr: **22013** Calculated *p*I: **6.40**

Probability Based Mow

Matched peptide sequences: shown in Bold Red

Matched peptides information:

Spot No.:**1502**

NCBI accession No.: **[GSBRNA2T00140846001](http://massboss/mascot/cgi/master_results.pl?file=../data/20150324/F016365.dat&REPTYPE=protein#Hit1)** Species: *Brassica napus*

PFF score: **76**

Protein name: **Germin-like protein subfamily 3 member 3**

Matched peptides No.: **1** Sequence coverage %: **10**

Calculated Mr: **22072** Calculated *p*I: **6.89**

Probability Based Mow

Matched peptide sequences: shown in Bold Red

Matched peptides information:

Spot No.: **1508**

NCBI accession No.: **[GSBRNA2T00051546001](http://massboss/mascot/cgi/master_results.pl?file=../data/20150311/F012783.dat&REPTYPE=protein#Hit1)** Species: *Brassica napus*

PFF score: **123**

Protein name: **Uncharacterized protein**

Matched peptides No.: **4**  Sequence coverage %: **27**

Calculated Mr: **18849** Calculated *p*I: **5.34**

Probability Based Mow

Matched peptide sequences: shown in Bold Red

Matched peptides information:

Spot No.: **1517**

NCBI accession No.: **[GSBRNA2T00115210001](http://massboss/mascot/cgi/master_results.pl?file=../data/20150329/F016527.dat&REPTYPE=protein#Hit1)** Species: *Brassica napus*

PFF score: **65**

Protein name: **Triosephosphate isomerase**

Matched peptides No.: **2**  Sequence coverage %: **10**

Calculated Mr: **27233** Calculated *p*I: **5.73**

Probability Based Mow

Matched peptide sequences: shown in Bold Red

Matched peptides information:

Spot No.: **1537**

NCBI accession No.: **[GSBRNA2T00133275001](http://massboss/mascot/cgi/master_results.pl?file=../data/20150311/F012785.dat&REPTYPE=protein#Hit1)** Species: *Brassica napus*

PFF score: **113**

Protein name: **Peptidyl-prolyl cis-trans isomerase CYP20-3**

Matched peptides No.: **1**  Sequence coverage %: **10**

Calculated Mr: **14533** Calculated *p*I: **9.44**

Probability Based Mow

Matched peptide sequences: shown in Bold Red

Matched peptides information:

Spot No.: **1580**

NCBI accession No.: **[GSBRNA2T00034112001](http://massboss/mascot/cgi/master_results.pl?file=../data/20150311/F012786.dat&REPTYPE=protein#Hit1)** Species: *Brassica napus*

PFF score: **229**

Protein name: **Eukaryotic translation initiation factor 5A-2**

Matched peptides No.: **5**  Sequence coverage %: **40**

Calculated Mr: **17316** Calculated *p*I:**5.56**

Probability Based Mow

Matched peptide sequences: shown in Bold Red

Matched peptides information:

Spot No.: **1588**

NCBI accession No.: **[GSBRNA2T00008050001](http://massboss/mascot/cgi/master_results.pl?file=../data/20150329/F016528.dat&REPTYPE=protein#Hit1)**  Species: *Brassica napus*

PFF score: **69**

Protein name: **unknown**

Matched peptides No.: **7**  Sequence coverage %: **9**

Calculated Mr: **68106** Calculated *p*I:**8.85**

Probability Based Mow

Matched peptide sequences: shown in Bold Red

Matched peptides information:

Spot No.: **1606**

NCBI accession No.: **[GSBRNA2T00053218001](http://massboss/mascot/cgi/master_results.pl?file=../data/20150311/F012788.dat&REPTYPE=protein#Hit1)**  Species: *Brassica napus*

PFF score: **245**

Protein name: **Uncharacterized protein**

Matched peptides No.: **4** Sequence coverage %: **8**

Calculated Mr: **24099** Calculated *p*I: **6.11**

Probability Based Mow

Matched peptide sequences: shown in Bold Red

Matched peptides information:

Spot No.: **1706**

NCBI accession No.: **[GSBRNA2T00074656001](http://massboss/mascot/cgi/master_results.pl?file=../data/20150311/F012792.dat&REPTYPE=protein#Hit1)** Species: *Brassica napus*

PFF score: **243**

Protein name: **Nucleoside diphosphate kinase 1**

Matched peptides No.: **5** Sequence coverage %: **31**

Calculated Mr: **16498** Calculated *p*I: **6.29**

Probability Based Mow

Matched peptide sequences: shown in Bold Red

Matched peptides information:

Spot No.: **1716**

NCBI accession No.: **[GSBRNA2T00007060001](http://massboss/mascot/cgi/master_results.pl?file=../data/20150311/F012793.dat&REPTYPE=protein#Hit1)** Species: *Brassica napus*

PFF score: **249**

Protein name: **Ribulose bisphosphate carboxylase small chain 1B**

Matched peptides No.: **5** Sequence coverage %: **27**

Calculated Mr: **20430** Calculated *p*I:**8.23**

Probability Based Mow

Matched peptide sequences: shown in Bold Red

Matched peptides information:

Spot No.: **1719**

NCBI accession No.: **[GSBRNA2T00007060001](http://massboss/mascot/cgi/master_results.pl?file=../data/20150311/F012794.dat&REPTYPE=protein#Hit1)** Species: *Brassica napus*

PFF score: **319**

Protein name: **Ribulose bisphosphate carboxylase small chain 1B**

Matched peptides No.: **6**  Sequence coverage %: **36**

Calculated Mr: **20430** Calculated *p*I: **8.23**

Probability Based Mow

Matched peptide sequences: shown in Bold Red

Matched peptides information:

Spot No.: **1727**

NCBI accession No.: **[GSBRNA2T00011400001](http://massboss/mascot/cgi/master_results.pl?file=../data/20150311/F012795.dat&REPTYPE=protein#Hit1)** Species: *Brassica napus*

PFF score: **608**

Protein name: **Ribulose bisphosphate carboxylase small chain 1B**

Matched peptides No.: **7**  Sequence coverage %: **45**

Calculated Mr: **20455** Calculated *p*I:**8.23**

Probability Based Mow

Matched peptide sequences: shown in Bold Red

Matched peptides information:

Spot No.: **1733**

NCBI accession No.: **[GSBRNA2T00007060001](http://massboss/mascot/cgi/master_results.pl?file=../data/20150311/F012796.dat&REPTYPE=protein#Hit1)** Species: *Brassica napus*

PFF score: **342**

Protein name: **Ribulose bisphosphate carboxylase small chain 1B**

Matched peptides No.: **7** Sequence coverage %: **23**

Calculated Mr: **20430** Calculated *p*I: **8.23**

Probability Based Mow

Matched peptide sequences: shown in Bold Red

Matched peptides information:

Spot No.: **1735**

NCBI accession No.: **[GSBRNA2T00011400001](http://massboss/mascot/cgi/master_results.pl?file=../data/20150311/F012797.dat&REPTYPE=protein#Hit1)** Species: *Brassica napus*

PFF score: **391**

Protein name: **Ribulose bisphosphate carboxylase small chain 1B**

Matched peptides No.: **7** Sequence coverage %: **45**

Calculated Mr: **20455**  Calculated *p*I: **8.23**

Probability Based Mow

Matched peptide sequences: shown in Bold Red

Matched peptides information:

Spot No.: **1740**

NCBI accession No.: **[GSBRNA2T00011400001](http://massboss/mascot/cgi/master_results.pl?file=../data/20150311/F012801.dat&REPTYPE=protein#Hit1)** Species: *Brassica napus*

PFF score: **609**

Protein name: **Ribulose bisphosphate carboxylase small chain 1B**

Matched peptides No.: **6**  Sequence coverage %: **38**

Calculated Mr: **20455** Calculated *p*I: **8.23**

Probability Based Mow

Matched peptide sequences: shown in Bold Red

Matched peptides information:

Spot No.: **1749**

NCBI accession No.: **[GSBRNA2T00007060001](http://massboss/mascot/cgi/master_results.pl?file=../data/20150311/F012798.dat&REPTYPE=protein#Hit1)** Species: *Brassica napus*

PFF score: **368**

Protein name: **Ribulose bisphosphate carboxylase small chain 1B**

Matched peptides No.: **6** Sequence coverage %: **37**

Calculated Mr: **20430** Calculated *p*I:**8.23**

Probability Based Mow

Matched peptide sequences: shown in Bold Red

Matched peptides information:

Spot No.: **1753**

NCBI accession No.: **[GSBRNA2T00007060001](http://massboss/mascot/cgi/master_results.pl?file=../data/20150311/F012800.dat&REPTYPE=protein#Hit1)** Species: *Brassica napus*

PFF score: **173**

Protein name: **Ribulose bisphosphate carboxylase small chain 1B**

Matched peptides No.: **4** Sequence coverage %: **19**

Calculated Mr: **20430** Calculated *p*I:**8.23**

Probability Based Mow

Matched peptide sequences: shown in Bold Red

Matched peptides information:

**Supplemental Figure S3**

**Classification and functional analysis of the DEPs under drought stress.** The theoretical and experimental ratios of M*r* and *p*I were determined and presented in radial chart as radial and annular radar axis labels respectively (A). Then, venn diagram illustrating differences in DEPs between mild drought treatment and severe drought treatment of *Brassica napus* leaves (B). Each protein was functionally classified by COG (C). The proportion of each functional category was the sum of the proportion of all identities. The subcellular locations of the identified 119 proteins were presented (D).

**Supplemental Figure S4**: **the main pathways.** KEGG pathway analysis was performed to determine their molecular interaction and reaction networks using KEGG database. (A) Enzymes exhibiting differential expression during drought stress are marked in red color. (B) Enzymes exhibiting phosphorylation and dephosphorylation during drought stress are marked in red color.

**(A) The main pathways for DEPs**

Carbon fixation in photosynthetic organisms (ath00710): 15 proteins

Glutathione metabolism (ath00480): 11 proteins

Ascorbate and aldarate metabolism (ath00053): 6 proteins

Glyoxylate and dicarboxylate metabolism (ath00630): 7 proteins

Photosynthesis (ath00195): 6 proteins

**(B) The main pathways for drought-responsive phosphorylated proteins**

Glycolysis/Gluconegenesis (ath00010): 10 proteins

Carbon fixation in photosynthetic organisms (ath00710): 7 proteins

Pentose phosphate pathway (ath00030): 6 proteins

Starch and sucrose metabolism (ath00500): 11 proteins

**Supplemental Table S1. Informations of differential expression protein spots.**

|  | **MD/CK** | |  | **Exp.** | **The.** |  |  |  | **Accession No.** | **Uniprot** | | **protein name** | **Gene product and** |  |
| --- | --- | --- | --- | --- | --- | --- | --- | --- | --- | --- | --- | --- | --- | --- |
| **NO.** | **SD/CK** | | **1-ANOVA** | **pI /Mr** | **pI /Mr** | **SC%** | **MP** | **M.S.** | **in genome** | **accessions** | | **Abbreviation** | **protein name** | **COG** |
| 120 | 1.02/-1.32 | | 1.30E-03 | 5.25/65.1 | 5.9/79.8 | 9 | 3 | 107 | GSBRNA2T00072539001 | Q8RWV0 | | TKL-1 | Transketolase-1, chloroplastic | G |
| 124 | 1.41/-1.07 | | 1.40E-05 | 5.62/65 | 6/79.6 | 11 | 4 | 85 | GSBRNA2T00149203001 | Q8RWV0 | | TKL-1 | Transketolase-1, chloroplastic | G |
| 127 | -1.15/-1.22 | | 8.40E-06 | 4.9/64.8 | 5.59/46.4 | 9 | 4 | 92 | GSBRNA2T00050483001 | P22953 | | MED37E | mediator of RNA polymerase II transcription subunit 37e | O |
| 139 | 1.19/1.32 | | 7.30E-06 | 4.6/64.4 | 4.84/66.8 | 18 | 9 | 454 | GSBRNA2T00000934001 | Q9LTX9 | | HSP70-7 | Heat shock 70 kDa protein 7, chloroplastic | O |
| 147 | 1.2/1.77 | | 3.90E-07 | 5.05/63.7 | 5.09/33.7 | 26 | 6 | 277 | GSBRNA2T00015387001 | Q8S7T5 | |  | ATP synthase subunit alpha | C |
| 150 | 1.12/1.38 | | 1.50E-02 | 4.98/63.7 | 7.77/20.1 | 15 | 5 | 122 | GSBRNA2T00113654001 | P22953 | | MED37E | mediator of RNA polymerase II transcription subunit 37e | O |
| 156 | -1.33/-1.61 | | 2.60E-06 | 4.86/63.5 | 5.14/71.4 | 8 | 5 | 136 | GSBRNA2T00007549001 | Q9LHA8 | | MED37C | mediator of RNA polymerase II transcription subunit 37c | O |
| 163 | -1.24/-1.29 | | 2.20E-05 | 4.92/63.4 | 7.77/20.1 | 15 | 3 | 144 | GSBRNA2T00113654001 | P22953 | | MED37E | mediator of RNA polymerase II transcription subunit 37e | O |
| 164 | -1.25/-1.01 | | 3.90E-05 | 4.96/63.3 | 5.14/71.4 | 7 | 5 | 134 | GSBRNA2T00007549001 | Q9LHA8 | | MED37C | mediator of RNA polymerase II transcription subunit 37c | O |
| 171 | -1/1.36 | | 5.90E-03 | 5.16/62.9 | 5.16/68.9 | 28 | 18 | 560 | GSBRNA2T00022872001 | O23654 | | VHA-A | V-type proton ATPase catalytic subunit A | C |
| 209 | -1.29/-1.28 | | 1.10E-02 | 4.92/61.9 | 5.74/76.6 | 9 | 6 | 114 | GSBRNA2T00048583001 | O80860 | | FTSH2 | ATP-dependent zinc metalloprotease FTSH 2 | O |
| 210 | -1.27/-1.08 | | 7.40E-03 | 4.97/61.9 | 5.74/76.6 | 14 | 7 | 423 | GSBRNA2T00048583001 | O80860 | | FTSH2 | ATP-dependent zinc metalloprotease FTSH 2 | O |
| 251 | -1.37/-1.22 | | 2.70E-03 | 4.58/60 | 5.02/61.5 | 13 | 6 | 254 | GSBRNA2T00138831001 | P21238 | | CPN60A1 | Chaperonin 60 subunit alpha 1, chloroplastic | O |
| 269 | -1.26/1.07 | | 7.20E-03 | 5.24/59.7 | 6.29/63.2 | 16 | 8 | 145 | GSBRNA2T00123626001 | Q9LJE4 | | CPN60B2 | Chaperonin 60 subunit beta 2, chloroplastic | O |
| 274 | -1.43/-1.12 | | 3.70E-04 | 4.62/59.7 | 5.15/61.9 | 18 | 9 | 324 | GSBRNA2T00045232001 | P21238 | | CPN60A1 | Chaperonin 60 subunit alpha 1, chloroplastic | O |
| 277 | 1.17/1.47 | | 7.30E-05 | 6.29/59.7 | 8.3/39.5 | 15 | 4 | 72 | GSBRNA2T00028808001 | NO | | NO | NO | NO |
| 282 | 1.28/2.01 | | 2.20E-09 | 6.44/59.5 | 8.86/61.5 | 5 | 3 | 68 | GSBRNA2T00004725001 | NO | | NO | NO | NO |
| 288 | -1.46/-1.2 | | 3.50E-05 | 4.56/59.4 | 5.06/63.3 |  | 6 | 301 | GSBRNA2T00118698001 | P21238 | | CPN60A1 | Chaperonin 60 subunit alpha 1, chloroplastic | O |
| 297 | -1.37/-1.24 | | 1.60E-05 | 4.52/59.3 | 4.82/55.9 | 6 | 4 | 106 | GSBRNA2T00066638001 | Q9XI01 | | PDIL1-1 | Protein disulfide isomerase-like 1-1 | O |
| 310 | -1.74/-1.51 | | 1.30E-06 | 5.29/59 | 6.99/67.3 | 12 | 7 | 151 | GSBRNA2T00034728001 | P21240 | | CPN60B1 | Chaperonin 60 subunit beta 1, chloroplastic | O |
| 311 | -1.69/-1.31 | | 4.90E-10 | 5.32/59 | 6.1/64.2 | 9 | 7 | 270 | GSBRNA2T00125571001 | P21240 | | CPN60B1 | Chaperonin 60 subunit beta 1, chloroplastic | O |
| 315 | -1.71/-1.16 | | 1.80E-08 | 5.38/58.9 | 5.54/75.3 | 15 | 9 | 226 | GSBRNA2T00114056001 | P21240 | | CPN60B1 | Chaperonin 60 subunit beta 1, chloroplastic | O |
| 349 | -1.28/-1 | | 1.40E-05 | 5.11/57.6 | 5.09/33.7 | 36 | 9 | 464 | GSBRNA2T00015387001 | NO | | NO | ATP synthase subunit alpha | C |
| 363 | 1.03/-1.31 | | 5.30E-06 | 4.87/57.5 | 5.09/33.7 | 32 | 9 | 188 | GSBRNA2T00015387001 | NO | | NO | ATP synthase subunit alpha | C |
| 376 | -1.28/-1.56 | | 4.70E-04 | 4.73/56.7 | 5.23/61.5 | 6 | 3 | 95 | GSBRNA2T00013010001 | Q8S9L5 | | TIG | Trigger factor-like protein TIG, Chloroplastic | NO |
| 378 | -1.29/-1.29 | | 5.10E-05 | 4.77/56.7 | 5.23/61.5 | 6 | 4 | 158 | GSBRNA2T00013010001 | Q8S9L5 | | TIG | Trigger factor-like protein TIG, Chloroplastic | NO |
| 383 | -1.81/1.07 | | 3.40E-07 | 4.56/56.4 | 5.15/48.5 | 2 | 1 | 85 | GSBRNA2T00091196001 | Q9M158 | | STR4 | Rhodanese-like domain-containing protein 4 | R |
| 390 | 1.11/1.47 | | 5.90E-03 | 4.93/56 | 5.03/54.1 | 45 | 15 | 558 | GSBRNA2T00003216001 | Q8W4E2 | | VHA-B3 | V-type proton ATPase subunit B3 | C |
| 414 | -1.04/-1.44 | | 6.30E-09 | 5.52/55.5 | 5.17/65.2 | 20 | 8 | 265 | GSBRNA2T00066465001 | NO | | ATPB | ATP synthase subunit beta, chloroplastic | C |
| 422 | -1.05/-1.35 | | 5.20E-06 | 5.56/55.3 | 5.01/14.6 | 16 | 2 | 129 | GSBRNA2T00106253001 | Q37247 | |  | Ribulose bisphosphate carboxylase large chain | C |
| 435 | 1.11/1.38 | | 9.40E-03 | 6.38/55.1 | 6.01/55.3 | 17 | 8 | 353 | GSBRNA2T00082162001 | F4IMB5 | | At2g07698 | ATP synthase subunit alpha | C |
| 444 | -1/1.22 | | 1.80E-07 | 5.15/54.5 | 5.17/65.2 | 16 | 8 | 475 | GSBRNA2T00066465001 | P0C2Z7 | | ATPB | ATP synthase subunit beta, chloroplastic | C |
| 447 | 1.11/-1.41 | | 6.80E-05 | 4.91/54.4 | 5.17/65.2 | 20 | 8 | 265 | GSBRNA2T00066465001 | P0C2Z7 | | ATPB | ATP synthase subunit beta, chloroplastic | C |
| 453 | 1.06/-1.3 | | 6.20E-05 | 4.97/54.3 | 5.17/65.2 | 29 | 11 | 815 | GSBRNA2T00066465001 | P0C2Z7 | | ATPB | ATP synthase subunit beta, chloroplastic | C |
| 464 | -1.23/-1.26 | | 3.70E-04 | 6.24/54 | 6.18/47.7 | 16 | 7 | 187 | GSBRNA2T00015407001 | NO | |  | Ribulose bisphosphate carboxylase large chain | C |
| 474 | -1.28/-1.03 | | 7.30E-02 | 5.36/53.5 | 5.15/61.9 | 13 | 6 | 211 | GSBRNA2T00045232001 | P21238 | | CPN60A1 | Chaperonin 60 subunit alpha 1, chloroplastic | O |
| 476 | -1.4/-1.04 | | 1.40E-07 | 5.5/53.8 | 5.52/16.7 | 34 | 4 | 86 | GSBRNA2T00059725001 | Q9M9P3 | | At3g03250 | UTP--glucose-1-phosphate uridylyltransferase 2 | G |
| 510 | 1.23/1.15 | | 6.30E-06 | 4.85/52.2 | 4.92/47.7 | 16 | 5 | 92 | GSBRNA2T00005941001 |  | | RPT5A | 26S protease regulatory subunit 6A homolog A | O |
| 516 | -1.41/-1.04 | | 2.70E-06 | 5.34/52 | 5.1/25 | 13 | 2 | 105 | GSBRNA2T00025997001 | F4JEL4 | | EIF4A1 | Translational initiation factor 4A-1 | J |
| 519 | 1.23/1.44 | | 2.40E-07 | 5.51/52 | 5.1/25 | 13 | 3 | 150 | GSBRNA2T00025997001 | F4JEL5 | | EIF4A2 | Translational initiation factor 4A-1 | J |
| 527 | -1.23/-1.05 | | 1.90E-05 | 6.33/52 | 5.83/44.8 | 17 | 6 | 163 | GSBRNA2T00153196001 | Q9LR30 | | GGAT1 | Glutamate--glyoxylate aminotransferase 1 | NO |
| 574 | -1.06/1.25 | | 2.50E-08 | 5.64/50.4 | 5.51/43.2 | 13 | 5 | 87 | GSBRNA2T00137491001 | Q9LUT2 | | METK4 | S-adenosylmethionine synthase 4 | H |
| 585 | -2.06/-2.19 | | 2.10E-09 | 4.62/50.2 | 5.4/44.8 | 10 | 4 | 184 | GSBRNA2T00071053001 | P25851 | | FBP | Fructose-1,6-bisphosphatase, chloroplastic | G |
| 586 | -1.94/-1.75 | | 6.10E-08 | 4.69/50.1 | 5.57/52.1 | 17 | 5 | 193 | GSBRNA2T00028746001 | P10896 | | RCA | Ribulose bisphosphate carboxylase/oxygenase activase | O |
| 587 | -1.1/-1.23 | | 2.10E-03 | 5.31/50 | 6.23/30.8 | 29 | 7 | 522 | GSBRNA2T00138076001 | P17745 | | TUFA | Elongation factor Tu, chloroplastic | J |
| 590 | -1.95/-1.64 | | 1.00E-10 | 4.76/50 | 5.57/52.1 | 28 | 10 | 507 | GSBRNA2T00028746001 | P10896 | | RCA | Ribulose bisphosphate carboxylase/oxygenase activase | O |
| 596 | -1.11/1.94 | | 1.10E-06 | 4.83/49.8 | 5.57/52.1 | 19 | 6 | 243 | GSBRNA2T00028746001 | P10896 | | RCA | Ribulose bisphosphate carboxylase/oxygenase activase | O |
| 597 | 1.21/1.33 | | 4.70E-04 | 4.92/49.8 | 5.09/47.1 | 11 | 5 | 158 | GSBRNA2T00049292001 | Q93WJ8 | | At5g03630 | monodehydroascorbate reductase, cytoplasmic isoform 4 | R |
| 608 | 1.07/1.22 | | 3.60E-07 | 5.12/49.3 | 6.29/51.5 | 14 | 4 | 149 | GSBRNA2T00012567001 |  | | RCA | Ribulose bisphosphate carboxylase/oxygenase activase | O |
| 628 | -1.6/-1.29 | | 5.90E-05 | 6.34/48.9 | 8.95/57.8 | 10 | 4 | 84 | GSBRNA2T00091532001 | Q93VR3 | | At5g28840 | GDP-mannose 3,5-epimerase | M |
| 647 | -1.33/1.01 | | 6.30E-07 | 5.33/48.3 | 5.5/35.7 | 18 | 6 | 296 | GSBRNA2T00107591001 | P53496 | | ACT11 | Actin-11 | Z |
| 674 | -1.24/-1.08 | | 5.40E-02 | 6.56/47.8 | 6.3/46.3 | 13 | 5 | 110 | GSBRNA2T00030156001 | Q9SRZ6 | | CICDH | Cytosolic isocitrate dehydrogenase [NADP] | C |
| 686 | -1.07/1.31 | | 1.10E-10 | 6.05/47.3 | 5.59/43 | 11 | 5 | 169 | GSBRNA2T00088488001 | P25857 | | GAPB | Glyceraldehyde-3-phosphate dehydrogenase GAPB | G |
| 706 | 1.35/1.72 | | 1.20E-06 | 4.59/46.2 | 5.07/47.4 | 8 | 2 | 104 | GSBRNA2T00064134001 | Q9SSA5 | | CYP38 | Peptidyl-prolyl cis-trans isomerase CYP38 | O |
| 725 | -1.63/-1.2 | | 1.60E-11 | 5.11/45.8 | 5.57/52.1 | 23 | 9 | 787 | GSBRNA2T00028746001 | P10896 | | RCA | Ribulose bisphosphate carboxylase/oxygenase activase | O |
| 728 | -1.35/-1.05 | | 1.00E-09 | 5.24/45.9 | 5.31/39.1 | 4 | 9 | 509 | GSBRNA2T00065379001 | Q9FMD9 | | GLN1-4 | Glutamine synthetase cytosolic isozyme 1-4 | E |
| 729 | 1.04/1.32 | | 3.10E-05 | 5.56/45.9 | 5.36/42.2 | 13 | 3 | 201 | GSBRNA2T00097384001 | Q9SAJ4 | | T8K14.3 | Phosphoglycerate kinase | G |
| 734 | -1.32/1.14 | | 1.10E-09 | 5.4/45.8 | 5.36/42.2 | 10 | 3 | 172 | GSBRNA2T00097384001 | Q9SAJ4 | | T8K14.3 | Phosphoglycerate kinase | G |
| 738 | 1.08/1.7 | | 5.00E-07 | 5.86/45.9 | 6.43/50.6 | 5 | 4 | 145 | GSBRNA2T00037717001 | P42799 | | GSA1 | Glutamate-1-semialdehyde 2,1-aminomutase 1 | H |
| 739 | -1.6/-1.59 | | 1.30E-06 | 4.9/45.6 | 6.27/48.3 | 17 | 4 | 329 | GSBRNA2T00155196001 | P10896 | | RCA | Ribulose bisphosphate carboxylase/oxygenase activase | O |
| 741 | -1.72/-1.42 | | 5.60E-08 | 5/45.7 | 6.27/48.2 | 24 | 9 | 565 | GSBRNA2T00155196001 | P10896 | | RCA | Ribulose bisphosphate carboxylase/oxygenase activase | O |
| 790 | -1.52/-1.32 | | 2.70E-01 | 6.39/44.1 | 6.16/39.3 | 17 | 4 | 108 | GSBRNA2T00020049001 | P42734 | | CAD9 | Probable cinnamyl alcohol dehydrogenase 9 | R |
| 813 | -1.09/-1.21 | | 8.70E-02 | 5.58/43.8 | 6.27/48.2 | 18 | 8 | 328 | GSBRNA2T00155196001 | P10896 | | RCA | Ribulose bisphosphate carboxylase/oxygenase activase | O |
| 842 | -1.37/-1.39 | | 2.00E-04 | 6.43/42.9 | 6.44/36.7 | 13 | 4 | 187 | GSBRNA2T00092896001 | Q9FX54 | | GAPC2 | Glyceraldehyde-3-phosphate dehydrogenase GAPC2 | G |
| 887 | 1.78/2.25 | | 7.20E-06 | 4.59/41.8 | 4.93/26.9 |  |  |  | GSBRNA2T00053446001 |  | | PCAP1 | Plasma membrane-associated cation-binding protein 1 | R |
| 901 | 1.29/1.58 | | 4.90E-06 | 5.07/41.8 | 6.78/42.9 | 12 | 3 | 108 | GSBRNA2T00136999001 | Q9SJU4 | | FBA1 | Probable fructose-bisphosphate aldolase 1 | G |
| 912 | 1.32/1.64 | | 6.80E-05 | 4.87/41.4 | 6.78/41.3 | 2 | 6 | 60 | GSBRNA2T00015652001 | Q01908 | | ATPC1 | ATP synthase gamma chain 1, chloroplastic | C |
| 913 | 1.35/1.88 | | 5.80E-08 | 4.97/41.5 | 6.78/42.9 | 20 | 5 | 104 | GSBRNA2T00136999001 | Q9SJU4 | | FBA1 | Probable fructose-bisphosphate aldolase 1 | G |
| 933 | 1.05/1.24 | | 1.20E-03 | 6.46/41 | 5.95/37.8 | 15 | 5 | 89 | GSBRNA2T00092222001 |  | | ESP | Epithiospecifier protein | R |
| 938 | 1.16/1.3 | | 2.00E-04 | 5.5/40.9 | 6.78/42.9 | 26 | 6 | 298 | GSBRNA2T00136999001 | Q9SJU4 | | FBA1 | Probable fructose-bisphosphate aldolase 1 | G |
| 947 | 1.2/1.46 | | 6.50E-05 | 5.98/40.5 | 6.26/38.1 | 9 | 2 | 67 | GSBRNA2T00064132001 | P27140 | | BCA1 | Beta carbonic anhydrase 1, chloroplastic | P |
| 976 | -1.36/-1.35 | | 2.50E-07 | 5.68/39.3 | 7.55/41.5 | 19 | 5 | 351 | GSBRNA2T00022009001 | P47999 | | OASB | Cysteine synthase, chloroplastic/chromoplastic | E |
| 1003 | 1.39/1.56 | | 2.00E-04 | 4.47/38.6 | 6.86/34.6 | 14 | 3 | 173 | GSBRNA2T00015621001 | O81439 | | PAP1 | Probable plastid-lipid-associated protein 1 | S |
| 1006 | 1.18/1.42 | | 6.20E-04 | 4.54/38.5 | 6.86/34.6 | 14 | 4 | 110 | GSBRNA2T00015621001 | O81439 | | PAP1 | Probable plastid-lipid-associated protein 1 | S |
| 1045 | 1.19/1.35 | | 2.10E-02 | 4.5/37 | 5.37/34.8 | 19 | 5 | 135 | GSBRNA2T00094536001 | O81439 | | PAP1 | Probable plastid-lipid-associated protein 1 | S |
| 1069 | 1.23/1.28 | | 2.70E-02 | 6.67/36.3 | 8.79/40.8 | 11 | 3 | 162 | GSBRNA2T00076633001 | Q8W493 | | LFNR2 | Ferredoxin--NADP reductase, leaf isozyme 2 | P |
| 1085 | 1.96/1.88 | | 2.40E-10 | 5.66/35.8 | 7.7/47.6 | 6 | 2 | 109 | GSBRNA2T00146509001 | Q42593 | | APXT | L-ascorbate peroxidase T, chloroplastic | P |
| 1086 | 1.29/1.26 | | 4.70E-08 | 6.08/35.8 | 5.57/52.1 | 22 | 8 | 468 | GSBRNA2T00028746001 | P10896 | | RCA | Ribulose bisphosphate carboxylase/oxygenase activase | O |
| 1117 | -1.16/-1.49 | | 8.20E-04 | 4.66/34.5 | 5.55/35.3 | 10 | 3 | 73 | GSBRNA2T00005668001 | P23321 | | PSBO1 | Oxygen-evolving enhancer protein 1-1 | NO |
| 1125 | 1.26/1.01 | | 7.20E-06 | 4.72/34.4 | 5.55/35.3 | 18 | 6 | 342 | GSBRNA2T00005668001 | P23321 | | PSBO1 | Oxygen-evolving enhancer protein 1-1 | NO |
| 1129 | -1.31/1.03 | | 7.50E-03 | 4.44/34.3 | 4.69/29.5 | 22 | 5 | 276 | GSBRNA2T00112035001 | Q96300 | | GRF7 | 14-3-3-like protein GF14 nu | T |
| 1138 | -1.35/-1.56 | | 1.30E-04 | 6.31/34.2 | 6.18/47.7 | 12 | 4 | 86 | GSBRNA2T00015407001 | NO | | NO | Ribulose bisphosphate carboxylase large chain | C |
| 1160 | 1.23/1.46 | | 2.30E-07 | 4.94/33.4 | 5.77/35.3 | 26 | 6 | 537 | GSBRNA2T00134966001 | P23321 | | PSBO1 | Oxygen-evolving enhancer protein 1-1 | NO |
| 1165 | -1.22/1.04 | | 1.20E-04 | 4.49/33.5 | 4.76/28.9 | 11 | 2 | 138 | GSBRNA2T00013874001 | P42644 | | GRF3 | 14-3-3-like protein GF14 psi | T |
| 1183 | -1.07/-1.62 | | 9.80E-08 | 6.13/33.2 | 6/29.9 | 7 | 3 | 74 | GSBRNA2T00066463001 |  | | RBCL | Ribulose bisphosphate carboxylase large chain | C |
| 1203 | -1.45/-1.14 | | 1.80E-02 | 5.71/32.6 | 8.4/34.3 | 24 | 5 | 200 | GSBRNA2T00103728001 | Q9SGS4 | | CDSP32 | Thioredoxin-like protein CDSP32, chloroplastic | O |
| 1212 | -1.13/-1.21 | | 3.20E-01 | 5.45/32.4 | 8.49/34.7 | 18 | 4 | 159 | GSBRNA2T00125328001 | Q94K71 | | At3g48420 | Haloacid dehalogenase-like hydrolase domain-containing protein | NO |
| 1214 | 1.08/1.26 | | 1.40E-05 | 4.95/32.4 | 8.3/34.8 | 10 | 4 | 62 | GSBRNA2T00068136001 | Q94K71 | | At3g48420 | Haloacid dehalogenase-like hydrolase domain-containing protein | NO |
| 1255 | 1.05/1.23 | | 1.60E-04 | 5.73/30.8 | 5.48/27.2 | 24 | 7 | 278 | GSBRNA2T00096646001 | O81147 | | PAA2 | Proteasome subunit alpha type-6-B | M |
| 1267 | 1.48/1.4 | | 2.50E-09 | 6.03/30.3 | 8.59/38.1 | 8 | 4 | 149 | GSBRNA2T00119786001 | P82281 | | TL29 | Thylakoid lumenal 29 kDa protein | O |
| 1271 | 1.12/1.32 | | 7.90E-06 | 5.66/29.9 | 5.58/27.8 | 34 | 6 | 397 | GSBRNA2T00100654001 | Q05431 | | APX1 | L-ascorbate peroxidase 1, cytosolic | V |
| 1272 | 1.25/1.21 | | 1.10E-04 | 5.69/30.1 | 5.86/28.2 | 36 | 8 | 432 | GSBRNA2T00122207001 | Q05431 | | APX1 | L-ascorbate peroxidase 1, cytosolic | P |
| 1279 | 1.1/1.29 | | 4.00E-05 | 5.3/29.9 | 6.9/33.4 | 21 | 4 | 281 | GSBRNA2T00090276001 | Q9SKP6 | | TIM | Triosephosphate isomerase, chloroplastic | P |
| 1285 | -1.22/-1.25 | | 4.10E-05 | 4.8/29.7 | 6.13/34.6 | 5 | 1 | 79 | GSBRNA2T00013748001 | Q9SU88 | | T16L4.100 | Putative uncharacterized protein T16L4.100 | G |
| 1286 | 1.44/2 | | 3.90E-10 | 5.82/29.7 | 5.58/27.8 | 50 | 9 | 464 | GSBRNA2T00100654001 | Q05431 | | APX1 | L-ascorbate peroxidase 1, cytosolic | NO |
| 1294 | -1.2/-1.07 | | 8.50E-06 | 4.87/29.6 | 5.33/28.4 | 4 | 1 | 111 | GSBRNA2T00035007001 | P04778 | | LHCB1.3 | Chlorophyll a-b binding protein 1 | P |
| 1304 | -1.55/-1.19 | | 5.00E-06 | 5.54/29.3 | 5.53/25.6 | 8 | 4 | 95 | GSBRNA2T00018693001 | O23708 | | PAB1 | Proteasome subunit alpha type-2-A | P |
| 1309 | -1.29/-1.32 | | 3.30E-05 | 4.95/29.3 | 5.13/31.9 | 25 | 5 | 149 | GSBRNA2T00119583001 | Q9LZ06 | | GSTL3 | Glutathione S-transferase L3 | O |
| 1319 | 1.74/1.31 | | 5.40E-05 | 4.41/29 | 6.26/38.1 | 17 | 4 | 317 | GSBRNA2T00064132001 | P27140 | | BCA1 | Beta carbonic anhydrase 1, chloroplastic | O |
| 1323 | -1.2/-1.1 | | 1.50E-06 | 6.05/28.8 | 5.73/27.2 | 5 | 3 | 108 | GSBRNA2T00115210001 | P48491 | | CTIMC | Triosephosphate isomerase, cytosolic | P |
| 1339 | -1.07/-1.25 | | 1.40E-05 | 5.47/36.1 | 5.47/36.1 | 63 | 15 | 6135 | gi|297787439 | P27140 | | BCA1 | Beta carbonic anhydrase 1, chloroplastic | G |
| 1340 | -1.24/-1.1 | | 4.30E-08 | 6.32/28.4 | 6.47/37.5 | 17 | 4 | 188 | GSBRNA2T00041298001 | P27140 | | BCA1 | Beta carbonic anhydrase 1, chloroplastic | P |
| 1351 | 1.03/1.23 | | 3.00E-03 | 5.72/28.1 | 5.76/23.6 | 12 | 2 | 200 | GSBRNA2T00043017001 | Q9FWR4 | | DHAR1 | Glutathione S-transferase DHAR1 | P |
| 1352 | 1.22/1.16 | | 1.70E-02 | 5.05/28 | 5.23/25.9 | 7 | 2 | 100 | GSBRNA2T00084355001 |  | | GSTU19 | Glutathione S-transferase U19 | P |
| 1355 | 4.62/5.46 | | 4.00E-13 | 4.93/28 | 5.61/28.2 | 20 | 4 | 171 | GSBRNA2T00049208001 | Q39101 | | FER1 | Ferritin-1, chloroplastic | P |
| 1357 | 1.37/1.34 | | 2.40E-03 | 4.53/28 | 6.27/48.3 | 17 | 4 | 100 | GSBRNA2T00155196001 | P10896 | | RCA | Ribulose bisphosphate carboxylase/oxygenase activase | P |
| 1360 | 1.5/1.17 | | 1.70E-09 | 5.9/27.9 | 5.47/36.1 | 55 | 14 | 630 | gi|297787439 | P27140 | | BCA1 | Beta carbonic anhydrase 1, chloroplastic | P |
| 1370 | 1.25/1.32 | | 2.50E-07 | 6.06/27.6 | 5.47/36.1 | 15 | 7 | 218 | gi|297787439 | P27140 | | BCA1 | Beta carbonic anhydrase 1, chloroplastic | P |
| 1372 | 1.11/1.39 | | 4.40E-07 | 6.1/27.5 | 6.04/25.8 | 17 | 5 | 149 | GSBRNA2T00025200001 | P46421 | | GSTU5 | Glutathione S-transferase U5 | O |
| 1379 | 1.11/1.29 | | 4.50E-07 | 6.34/27.4 | 6.37/25.7 | 15 | 3 | 105 | GSBRNA2T00099235001 | Q9ZRW8 | | GSTU19 | Glutathione S-transferase U19 | P |
| 1415 | -1.36/-1.5 | | 0.00E+00 | 6.43/26.6 | 5.98/24.2 | 10 | 3 | 113 | GSBRNA2T00043389001 | O80852 | | GSTF9 | Glutathione S-transferase F9 | O |
| 1426 | 1.24/1.16 | | 1.00E-02 | 4.64/26.2 | 4.73/25.7 | 19 | 2 | 116 | GSBRNA2T00044206001 | Q9SHG8 | | GSTF9 | Glutathione S-transferase F9 | P |
| 1430 | 1.6/1.45 | | 3.00E-04 | 4.54/26 | 5/27.5 | 15 | 2 | 66 | GSBRNA2T00066856001 | Q9C5R8 | | At5g06290 | 2-Cys peroxiredoxin BAS1-like, chloroplastic | P |
| 1478 | 1.54/1.32 | | 2.50E-08 | 5.43/24.5 | 6/31.6 | 21 | 6 | 175 | GSBRNA2T00002240001 | F4IT21 | | At2g43945 | Uncharacterized protein | P |
| 1492 | 1.15/1.38 | | 1.30E-04 | 6.08/23.4 | 6.4/22 | 10 | 1 | 60 | GSBRNA2T00004321001 | P94072 | | GER3 | Germin-like protein subfamily 3 member 3 | O |
| 1502 | 1.06/1.24 | | 8.00E-03 | 6.66/23.1 | 6.89/22 | 10 | 1 | 76 | GSBRNA2T00140846001 | P94072 | | GER3 | Germin-like protein subfamily 3 member 3 | O |
| 1508 | -1.66/-1.46 | | 3.50E-05 | 5.39/23 | 5.34/18.8 | 27 | 4 | 123 | GSBRNA2T00051546001 | Q9ZPZ4 | | T31J12.3 | Uncharacterized protein | O |
| 1517 | 1.07/1.29 | | 2.50E-02 | 6.08/22.5 | 5.73/27.2 | 10 | 2 | 65 | GSBRNA2T00115210001 | P48491 | | CTIMC | Triosephosphate isomerase, cytosolic | O |
| 1537 | 1.19/1.39 | | 4.10E-01 | 5.49/21 | 9.44/14.5 | 10 | 1 | 113 | GSBRNA2T00133275001 | P34791 | | CYP20-3 | Peptidyl-prolyl cis-trans isomerase CYP20-3 | S |
| 1580 | -1.39/-1.13 | | 4.30E-07 | 5.37/19.3 | 5.56/17.3 | 40 | 5 | 229 | GSBRNA2T00034112001 | Q93VP3 | | ELF5A-2 | Eukaryotic translation initiation factor 5A-2 | O |
| 1588 | 2.03/1.3 | | 1.40E-01 | 5.73/18.8 | 8.85/68.1 | 9 | 7 | 69 | GSBRNA2T00008050001 | NO | | NO | NO | NO |
| 1606 | -1.33/-1.34 | | 2.60E-03 | 5.05/18 | 6.77/24 | 31 | 4 | 245 | GSBRNA2T00053218001 | Q9LVM3 | | At5g58250 | Uncharacterized protein | NO |
| 1706 | -1.39/-1.34 | | 1.10E-10 | 6.43/14.4 | 6.29/16.4 | 31 | 5 | 243 | GSBRNA2T00074656001 | P39207 | | NDK1 | Nucleoside diphosphate kinase 1 | NO |
| 1716 | -1.05/-1.68 | | 4.20E-08 | 5.56/13.8 | 8.23/20.4 | 27 | 5 | 249 | GSBRNA2T00007060001 | P10796 | | RBCS-1B | Ribulose bisphosphate carboxylase small chain 1B | S |
| 1719 | -1.11/-1.75 | | 5.80E-07 | 5.61/13.7 | 8.23/20.4 | 36 | 6 | 319 | GSBRNA2T00007060001 | P10796 | | RBCS-1B | Ribulose bisphosphate carboxylase small chain 1B | G |
| 1727 | -1.11/-1.63 | | 3.50E-07 | 5.52/13.7 | 8.23/20.4 | 45 | 7 | 608 | GSBRNA2T00011400001 | P10796 | | RBCS-1B | Ribulose bisphosphate carboxylase small chain 1B | O |
| 1733 | -1.12/-1.67 | | 6.00E-05 | 6.34/13.5 | 8.23/20.4 | 23 | 7 | 342 | GSBRNA2T00007060001 | P10796 | | RBCS-1B | Ribulose bisphosphate carboxylase small chain 1B | J |
| 1735 | -1.11/-1.64 | | 4.90E-05 | 6.23/13.4 | 8.23/20.4 | 45 | 7 | 391 | GSBRNA2T00011400001 | P10796 | | RBCS-1B | Ribulose bisphosphate carboxylase small chain 1B | NO |
| 1749 | -1.01/-2 | | 1.50E-10 | 5.06/12.4 | 8.23/20.4 | 37 | 6 | 368 | GSBRNA2T00007060001 | P10796 | | RBCS-1B | Ribulose bisphosphate carboxylase small chain 1B | NO |
| 1753 | 1.11/-1.86 | | 6.00E-07 | 5.03/12.3 | 8.23/20.4 | 19 | 4 | 173 | GSBRNA2T00007060001 | P10796 | | RBCS-1B | Ribulose bisphosphate carboxylase small chain 1B | F |
| 1740 | -1.01/-1.37 | | 8.70E-03 | 6.51/13.4 | 8.23/20.4 | 38 | 6 | 609 | GSBRNA2T00011400001 | P10796 | | RBCS-1B | Ribulose bisphosphate carboxylase small chain 1B | C |
| 1423 | -1.07/1.23 | | 6.50E-09 | 6.16/26.3 | 5.99/24.4 | 7 | 2 | 67 | GSBRNA2T00101634001 | Q96266 | | GSTF8 | Glutathione S-transferase F8, chloroplastic | C |
| 1234 | -1.08/1.32 | | 9.30E-04 | 6.08/31.4 | 6.19/27.2 | 8 | 2 | 85 | GSBRNA2T00032385001 | Q94EG6 | | At5g02240 | Uncharacterized protein At5g02240 | C |
| 1331 | 1.08/-1.16 | | 5.30E-04 | 5.36/27.9 | 5.47/36.1 | 27 | 6 | 493 | gi|297787439 | P27140 | | BCA1 | Beta carbonic anhydrase 1, chloroplastic | C |
| 1328 | 1.21/1.01 | | 1.00E-06 | 5.59/27.9 | 5.47/36.1 | 26 | 7 | 586 | gi|297787439 | P27140 | | BCA1 | Beta carbonic anhydrase 1, chloroplastic | C |
| 1343 | -1.06/-1.16 | | 1.20E-04 | 5.68/27.9 | 5.47/36.1 | 15 | 3 | 275 | gi|297787439 | P27140 | | BCA1 | Beta carbonic anhydrase 1, chloroplastic | C |
| 1361 | -1.23/-1.11 | | 5.70E-09 | 5.92/27.9 | 5.47/36.1 | 23 | 5 | 489 | gi|297787439 | P27140 | | BCA1 | Beta carbonic anhydrase 1, chloroplastic | C |
| 1327 | 1.14/-1.28 | | 2.70E-03 | 5.32/27.9 | 5.47/36.1 | 7 | 2 | 159 | gi|297787439 | P27140 | | BCA1 | Beta carbonic anhydrase 1, chloroplastic | C |
|  | | Note: | | | | | | | | |  | | | |
|  | | NO.: Assigned spot number as indicated in Fig. 2 | | | | | | | | |  | | | |
|  | | The.*p*I /M*r*: The theoretical *p*I and mass (kDa) of the identified proteins | | | | | | | | |  | | | |
|  | | Exp.*p*I /M*r*: The experimental *p*I and mass (kDa) of the identified proteins | | | | | | | | |  | | | |
|  | | SC: The amino acid sequence coverage for the identified proteins | | | | | | | | |  | | | |
|  | | MP: Number of the matched peptides | | | | | | | | |  | | | |
|  | | M.S.: The Mascot searched score against the database of *Brassica napus* genome. | | | | | | | | |  | | | |

**Supplemental Table S2. DEPs identified from two to eight DEP spots on 2D-DIGE maps.**

| Protein  NO. | Description | DEP spot  IDs | Experimental  pI | Experimental  Mr |
| --- | --- | --- | --- | --- |
| GSBRNA2T00015387001 | ATP synthase subunit alpha | 147/349/363 | 5.05/5.11/4.87 | 63.7/57.6/57.5 |
| GSBRNA2T00066465001 | ATP synthase subunit beta | 414/444/447/453 | 5.52/5.15/4.91/4.97 | 55.4/54.5/54.4/54.4 |
| GSBRNA2T00048583001 | ATP-dependent zinc metalloprotease FTSH 2, | 209/210 | 4.92/4.97 | 61.9/61.9 |
| gi|297789439 | Beta carbonic anhydrase 1, | 1327/1331/1328/  1339/1343/1360/  1361/1371 | 5.31/5.36/5.39/  5.47/5.68/5.90/  5.92/6.06 | 27.9/27.9/27.9/  27.9/27.9/27.9/  27.9/27.7 |
| GSBRNA2T00045232001 | Chaperonin 60 subunit alpha 1 | 274/474 | 4.62/5.36 | 59.7/53.6 |
| GSBRNA2T00100654001 | L-ascorbate peroxidase 1 | 1271/1286 | 5.66/5.82 | 29.9/29.8 |
| GSBRNA2T00005668001 | Oxygen-evolving enhancer protein 1-1, | 1117/1125 | 4.66/4.72 | 34.5/34.4 |
| GSBRNA2T00097384001 | Phosphoglycerate kinase | 729/734 | 5.56/5.4 | 45.9/45.8 |
| GSBRNA2T00136999001 | Probable fructose-bisphosphate aldolase 1 | 901/913/938 | 5.07/4.97/5.5 | 41.8/41.5/41.0 |
| GSBRNA2T00007549001 | Probable mediator of RNA polymerase II transcription subunit 37c | 150/164 | 4.86/4.96 | 63.4/63.3 |
| GSBRNA2T00113654001 | Probable mediator of RNA polymerase II transcription subunit 37e | 156/163 | 4.98/4.92 | 63.7/63.4 |
| GSBRNA2T00015621001 | plastid-lipid-associated protein | 1003/1006 | 4.47/4.54 | 38.6/38.5 |
| GSBRNA2T00015407001 | Ribulose bisphosphate carboxylase large chain | 464/1138 | 6.24/6.31 | 54.0/34.2 |
| GSBRNA2T00007060001 | Ribulose bisphosphate carboxylase small chain 1B | 1716/1719/1733/1749/1753 | 5.56/5.61/6.34/5.06/5.03 | 13.8/13.8/13.5/12.5/12.3 |
| GSBRNA2T00011400001 | Ribulose bisphosphate carboxylase small chain 1B | 1727/1735/1740 | 5.52/6.23/6.51 | 13.7/13.5/13.4 |
| GSBRNA2T00028746001 | Ribulose bisphosphate carboxylase/oxygenase activase | 586/590/596/725 | 4.69/4.76/4.83/5.11 | 50.2/50.0/50.0/45.9 |
| GSBRNA2T00155196001 | Ribulose bisphosphate carboxylase/oxygenase activase | 739/741/813/1357 | 4.9/5.0/5.58/4.53 | 45.7/45.7/43.9/28.0 |
| GSBRNA2T00025997001 | Translational initiation factor 4A-1 | 516/519 | 5.34/5.51 | 52.0/52.0 |
| GSBRNA2T00013010001 | Trigger factor-like protein TIG | 376/378 | 4.73/4.77 | 56.7/56.7 |
| GSBRNA2T00115210001 | Triosephosphate isomerase | 1323/1517 | 6.05/6.08 | 28.8/22.6 |

**Supplemental Table S3. Information of PPI networks.**

| Protein name | COG number | COG |
| --- | --- | --- |
| Transketolase | COG0021 | G |
| Cysteine synthase | COG0031 | E |
| Glyceraldehyde-3-phosphate dehydrogenase/erythrose-4-phosphate dehydrogenase | COG0057 | G |
| 3-phosphoglycerate kinase | COG0126 | G |
| Triosephosphate isomerase | COG0149 | G |
| Glutamine synthetase | COG0174 | E |
| S-adenosylmethionine synthetase | COG0192 | H |
| F_0_F_1_-type ATP synthase, gamma subunit | COG0224 | C |
| Carbonic anhydrase | COG0288 | P |
| Catalase (peroxidase I) | COG0376 | P |
| Aspartate/tyrosine/aromatic aminotransferase | COG0436 | NO |
| Molecular chaperone | COG0443 | O |
| Uncharacterized NAD(FAD)-dependent dehydrogenases | COG0446 | C |
| Chaperonin GroEL (HSP60 family) | COG0459 | O |
| ATP-dependent Zn proteases | COG0465 | O |
| Thiol-disulfide isomerase and thioredoxins | COG0526 | O |
| Isocitrate dehydrogenases | COG0538 | C |
| Glutathione S-transferase | COG0625 | O |
| Predicted phosphatase/phosphohexomutase | COG0637 | NO |
| Archaeal/vacuolar-type H^+^-ATPase subunit A | COG1155 | C |
| Archaeal/vacuolar-type H^+^-ATPase subunit B | COG1156 | C |
| ATP-dependent 26S proteasome regulatory subunit | COG1222 | O |
| Beta-glucosidase/6-phospho-beta-glucosidase/beta-galactosidase | COG2723 | G |
| Fructose-1,6-bisphosphate aldolase | COG3588 | G |
| Ribulose bisphosphate carboxylase small subunit | COG4451 | C |
| 14-3-3 family protein | COG5040 | T |
| Actin and related proteins | COG5277 | Z |
| non supervised orthologous group | NOG00936 | NO |
| non supervised orthologous group | NOG07766 | NO |
| non supervised orthologous group | NOG125096 | NO |
| non supervised orthologous group | NOG52505 | NO |
| Archaeal/vacuolar-type H^+^-ATPase subunit I | COG1269 | C |
| Archaeal/vacuolar-type H^+^-ATPase subunit D | COG1394 | C |
| F_0_F_1_-type ATP synthase, alpha subunit | COG0056 | C |
| F_0_F_1_-type ATP synthase, epsilon subunit (mitochondrial delta subunit) | COG0355 | C |
| F_0_F_1_-type ATP synthase, beta subunit | COG0055 | C |
| F_0_F_1_-type ATP synthase, delta subunit (mitochondrial oligomycin sensitivity protein) | COG0712 | C |
| Archaeal/vacuolar-type H^+^-ATPase subunit C | COG1527 | C |
| Archaeal/vacuolar-type H^+^-ATPase subunit E | COG1390 | C |
| F_0_F_1_-type ATP synthase, subunit a | COG0356 | C |
| Ribulose 1,5-bisphosphate carboxylase, large subunit | COG1850 | G |

| **Supplemental Table S4. Phosphorylation sites of the eight BCA1 protein spots from 2D gel.** | | | | | |
| --- | --- | --- | --- | --- | --- |
| **Spot NO.** | **Protein accession NO.** | | **phosphorylated peptide and site** | **Modifications** | **Conf** |
| 1327 | | gi|297787539 | VCPSHVLNFQPGEAFVVR | Phospho(S)@4 | 96.7 |
|  | |  | VENIVVIGHSA | Phospho(S)@10 | 98.6 |
| 1328 | | gi|297787539 | PVENIKQGFITFKKEKYETNPALYGELAK | Phospho(T)@11 | 96.2 |
|  | |  | EKYETNPALYGELAK | Phospho(T)@5 | 97.9 |
|  | |  | EKYETNPALYGELAKGQSPKYMVFA | Phospho(S)@18 | 98.4 |
|  | |  | VCPSHVLNFQPGEAFVVR | Phospho(S)@4 | 98.7 |
|  | |  | YGGVGAAIEYAVLHLK | Phospho(Y)@10 | 99 |
|  | |  | VENIVVIGHSACGGIK | Phospho(S)@10 | 99 |
|  | |  | GLMSFALDGNNSTDFIEDWVK | Phospho(S)@4 | 99 |
| 1331 | | gi|297787539 | PVENIKQGFITFKKEKYETNPALYGELAK | Phospho(T)@11 | 98 |
|  | |  | VENIVVIGHSACGGIK | Phospho(S)@10 | 99 |
|  | |  | GLMSFPLDGNNSTDFIEDWVK | Phospho(S)@12 | 95.5 |
|  | |  | GLMSFALDGNNSTDFIEDWVK | Phospho(S)@4 | 99 |
| 1339 | | gi|297787539 | YGGVGAAIEYAVLHLK | Phospho(Y)@10 | 99 |
|  | |  | GDSAFEDQCGRCE | Phospho(S)@3 | 96.7 |
| 1343 | | gi|297787539 | QGFITFKKEKYETNPALYGELAK | Phospho(T)@5 | 99 |
|  | |  | YMVFACSDSR | Phospho(S)@7 | 98 |
|  | |  | VENIVVIGHSACGGIK | Phospho(S)@10 | 96.2 |
|  | |  | GLMSFPLDGNNSTDFIEDWVK | Phospho(S)@12 | 98.9 |
| 1360 | | gi|297787539 | VCPSHVLNFQPGEAFVVR | Phospho(S)@4 | 98.7 |
|  | |  | GDSAFEDQCGRCE | Phospho(S)@3 | 96.7 |
|  | |  | VISELGDSAFEDQCGRCEREAVNVSLANLLTYPFVREGLVK | Phospho(S)@25 | 99 |
| 1361 | | gi|297787539 | ATAALQTGTSSDKK | Phospho(T)@2 | 99 |
|  | |  | VEQATAALQTGTSSDKK | Phospho(T)@10 | 99 |
|  | |  | PVENIKQGFITFKKEKYETNPALYGELAK | Phospho(T)@11 | 99 |
|  | |  | PSHVLNFQPGEAFVVR | Phospho(S)@2 | 97.2 |
|  | |  | YGGVGAAIEYAVLHLK | Phospho(Y)@10 | 99 |
|  | |  | VENIVVIGHSA | Phospho(S)@10 | 96.8 |
|  | |  | IKGLMSFPLDGNNSTDFIEDWVK | Phospho(S)@6 | 99 |
|  | |  | VISELGDSAFEDQCGRCEREAVNVSLANLLTYPFVREGLVK | Phospho(S)@25 | 99 |
|  | |  | VISELGDSAFEDQCGRCEREAVNVSLANLLT | Phospho(T)@31 | 97.9 |
|  | |  | GGYYDFIK | Phospho(Y)@3 | 95.6 |

**Supplemental Table S5. Primer sequences used for qRT-PCR analysis.**

| **DEP spot No.** | **Proteins Name Abbreviation** | **Protein gi No.** | **Sense primer (5'-3')** | **Antisense primer (5'-3')** |
| --- | --- | --- | --- | --- |
|  |  |  |  |  |
| 124 | TKL-1 | GSBRNA2T00072539001 | CAAGACACCTGGACATCCTGAG | GCTGCTTCGTTTGAAATACCCT |
| 376 | TIG | GSBRNA2T00013010001 | CTTCAAGGTGGTGCATCCG | GGCCAACGTGCTCAGTTCA |
| 596 | RCA | GSBRNA2T00028746001 | GTCTTCTTTGACAGCAACCACTTT | AGTCTCCTCATCCGTCAACCA |
| 1355 | FER1 | GSBRNA2T00049208001 | TTGCGAGGCTGCCATTAAC | AGATAGGGGAGACGATAGGGTG |
| 1360 | BCA1 | GSBRNA2T00064132001 | AAACAATGTGAAAGAACAAGAGCAC | ACGAGGATAATGAAAGCGATGG |
| 1508 | Uncharacterized protein | GSBRNA2T00051546001 | GCCCAATGGCTTACTACCGT | CTTGTGGGTGATGCTCTGTTTC |
